# Genome wide analysis of protein production load in *Trichoderma reesei*

**DOI:** 10.1186/s13068-016-0547-5

**Published:** 2016-06-28

**Authors:** Tiina M. Pakula, Heli Nygren, Dorothee Barth, Markus Heinonen, Sandra Castillo, Merja Penttilä, Mikko Arvas

**Affiliations:** VTT Technical Research Centre of Finland, Tietotie 2, P.O. Box FI-1000, 02044 Espoo, Finland; Department of Information and Computer Science, Aalto University, PO Box 15400, 00076 Espoo, Finland; Helsinki Institute for Information Technology HIIT, Espoo, Finland

**Keywords:** Protein production, Transcriptomics, *Trichoderma reesei*, Hypocrea jecorina, Flux balance analysis, Metabolic modelling, Stoichiometric model, RNA sequencing

## Abstract

**Background:**

The filamentous fungus *Trichoderma reesei* (teleomorph *Hypocrea jecorina*) is a widely used industrial host organism for protein production. In industrial cultivations, it can produce over 100 g/l of extracellular protein, mostly constituting of cellulases and hemicellulases. In order to improve protein production of *T. reesei* the transcriptional regulation of cellulases and secretory pathway factors have been extensively studied. However, the metabolism of *T. reesei* under protein production conditions has not received much attention.

**Results:**

To understand the physiology and metabolism of *T. reesei* under protein production conditions we carried out a well-controlled bioreactor experiment with extensive analysis. We used minimal media to make the data amenable for modelling and three strain pairs to cover different protein production levels. With RNA-sequencing transcriptomics we detected the concentration of the carbon source as the most important determinant of the transcriptome. As the major transcriptional response concomitant to protein production we detected the induction of selected genes that were putatively regulated by *xyr1* and were related to protein transport, amino acid metabolism and transcriptional regulation. We found novel metabolic responses such as production of glycerol and a cellotriose-like compound. We then used this cultivation data for flux balance analysis of *T. reesei* metabolism and demonstrate for the first time the use of genome wide stoichiometric metabolic modelling for *T. reesei*. We show that our model can predict protein production rate and provides novel insight into the metabolism of protein production. We also provide this unprecedented cultivation and transcriptomics data set for future modelling efforts.

**Conclusions:**

The use of stoichiometric modelling can open a novel path for the improvement of protein production in *T. reesei*. Based on this we propose sulphur assimilation as a major limiting factor of protein production. As an organism with exceptional protein production capabilities modelling of *T. reesei* can provide novel insight also to other less productive organisms.

**Electronic supplementary material:**

The online version of this article (doi:10.1186/s13068-016-0547-5) contains supplementary material, which is available to authorized users.

## Background

The filamentous fungus *Trichoderma reesei* (teleomorph *Hypocrea jecorina*) is a widely used industrial host organism for protein production. In industrial cultivations, it can produce over 100 g/l concentrations of extracellular protein [1]. Interestingly, it exhibits a low growth rate protein production phenotype [2], i.e. the highest rates of cellulase and hemicellulase secretions are observed at low growth rate [3–6].

In industrial environments such conditions can exist for a prolonged period during the feeding stage of a fed-batch cultivation. However, many industrial processes are batch cultivations with constantly changing growth rates. In both conditions *T. reesei* efficiently produces cellulases and also heterologous proteins under cellulase promoters, given a cellulase expression inducing carbon source. Cellulase expression is well induced by sophorose (for review see [7, 8]), but also by cellobiose [9], lactose [10] and cellobiono-1,5-lactone [11]. Recent transcriptome analyses of cellulase producing conditions, have revealed various responses to protein production: partial induction of the secretion system, induction and carbon source specific modulation of cellulases, but also complex metabolic responses whose importance and regulation remains to be discovered [6, 12–18]. The major regulators of cellulases are transcription factors *cre1* [19] and *xyr1* [20] (for review see [8]), but also *ace1* [21], *ace2* [22], *ace3* [15], *clr2* [23] and other Zn2Cys6 zinc cluster transcription factors [15], GCN5-like histone acetyl transferases [6] and histone methyltransferases [24] have been implicated.

The level of endoplasmic reticulum (ER) stress in protein producing conditions is typically unclear in published *T. reesei* transcriptome analysis. Induction of the unfolded protein response (UPR), the conserved eukaryotic stress response to high ER load, can be assessed by non-conventional splicing of the *hac1*-intron [25] and hence is not detectable from microarray analysis. Major factors like foldase *bip1* [26] and *pdi1* [27] are typically induced. However, in *Saccharomyces cerevisiae*, the expression of *BIP1* and *PDI1* has been shown to be a poor indicator of UPR [28].

With the secretion pathway factors (for review see [29]) and cellulase transcription factors extensively studied, new approaches for improving protein production in *T. reesei* are needed. Genome scale stoichiometric metabolic modelling has been very successfully applied to improvement of production of a single metabolite (for review see [30]). In this field, the stoichiometric matrix, i.e. a mathematical description of most biochemical reactions of the cell, is analyzed to select enzyme genes to be removed, regulated or added, or to optimize cultivation strategies in order to increase metabolite production. In contrast, successful application of stoichiometric modelling to improve protein production has been rare, although various other strategies for engineering metabolite supply for protein production have been used (for review see [31]). A notable exception is analysis of super oxide dismutase production with stoichiometric modelling in *Komagataella phaffii (Pichia pastoris)* [32]. Filamentous fungi, such as *T. reesei*, can reach far higher production levels and are also in many other respect distinct organism from yeasts. Stoichiometric modelling of *T. reesei* has not been, to our knowledge, reported previously. Also, successful stoichiometric modelling typically requires growth conditions rarely used in published *T. reesei* work i.e bioreactor cultivations, extensive culture monitoring and a defined carbon source.

In this paper we carry out a well controlled bioreactor experiment to study the effect of variation of protein production load to the physiology of *T. reesei*. Our RNA-sequencing transcriptomics detects the concentration of carbon source as the most important determinant of transcriptome. We extensively analyze the growth medium during cultivation and find novel metabolic responses such as production of glycerol and a cellotriose-like compound. We then use this cultivation data for flux balance analysis of the metabolism of *T. reesei* and demonstrate for the first time the use of genome wide stoichiometric metabolic modelling for *T. reesei*. We show that our model can predict protein production rate and provides novel insight into the metabolism of protein production. We also provide this unprecedented cultivation and transcriptomics data set for future modelling efforts

## Results

The effect of protein production load on the physiology of *T. reesei* was studied by comparing sixstrains (three production strains and their three controls) that were modified for their protein production properties.

**Table 1:** Description of strains

Abbreviation	Strain	Extracellular proteins produced
**Cel4d**	$\Delta$ *cbh1,* $\Delta$ *cbh2*, $\Delta$ *egl1*, $\Delta$ *egl2*, $\Delta$ *mus53*	Endogenous hydrolases, lacking CBHI, CBHII, EGI, EGII
**Cel4dCt**	$\Delta$ *mus53*	Endogenous hydrolases
**CutCBHd**	oe *Cut*, $\Delta$ *cbh1*,	Endogenous hydrolases, lacking CBHI. Heterologous protein (cutinase, cut)
**CutCBHdCt**	$\Delta$ *cbh1*	Endogenous hydrolases, lacking CBHI
**LipPr4d**	oe Lip, $\Delta$ *mus53*	Endogenous hydrolases. Heterologous protein (lipase, Lip). Deletion of four native proteases
**LipPr4dCt**	$\Delta$ *mus53*	Endogenous hydrolases. Deletion of four native proteases

The selected strains included *T. reesei* (**Cel4d**) from which the four main cellulase genes (*chb1*, *cbh2*, *egl1* and *egl2*) have been deleted as well as *T. reesei* (**Cel4dCt**) producing the wild type pattern of cellulases. Under typical production conditions, the four major cellulases may account for over 90  % of the extracellular proteins produced by the hypercellulolytic strains under typical production conditions. The cellobiohydrolases have been reported to account for up to 64–84  % and the major endoglucanases up to 4–36  % of the extracellular protein produced [33–35]. Thus, the deletion of the cellulase genes is expected to have a major impact on the protein mixture produced. For analysing the effects of producing heterologous proteins with different properties and at different amounts, strains producing either a lipase from *Dipodascus capitatus* (**LipPr4d**) or a cutinase from *Coprinus cinerea* (**CutCBH**) were included in the study together with their control strains. The corresponding control strains had similar genetic modifications as the recombinant protein producers except for the gene encoding the heterologous product (Table 1). *D. capitatus* lipase is readily produced by *T. reesei*, reaching almost 20 % of the proteins produced at early stages of cultivations in the study (see below), whereas production of the cutinase of *C. cinerea* was hardly detectable in the cultures. The lipase is apparently sensitive to *T. reesei* proteases under the conditions studied and therefore a production strain (**LipPr4d**) with four protease-encoding genes deleted was used. The corresponding control strain (**LipPr4dCt**) had the same protease deletions. In the cutinase producing strain, the *cbh1* locus was used for expression and thus, both the cutinase producing strain (**CutCBHd**) and its control strain (**CutCBHdCt**) lack the open reading frame encoding cellobiohydrolase I (CBHI). CBHI alone may constitute 60 % of the total protein [36]. Furthermore, the strain with wild type cellulase gene pattern (**Cel4dCt**), the strain deleted for the four cellulase genes (**Cel4d**) as well as the lipase producing strain (**LipPr4d**) and its control strain (**LipPr4dCt**) had a deletion in the gene *mus53*. The *mus53* modification was originally done in order to help construction of modified strains by enhancing homologous recombination in the strain construction process [37]. The abbreviations used for the strains, the genetic modifications in the strains as well as their major properties are shown in Table 1.

**Fig. 1:**
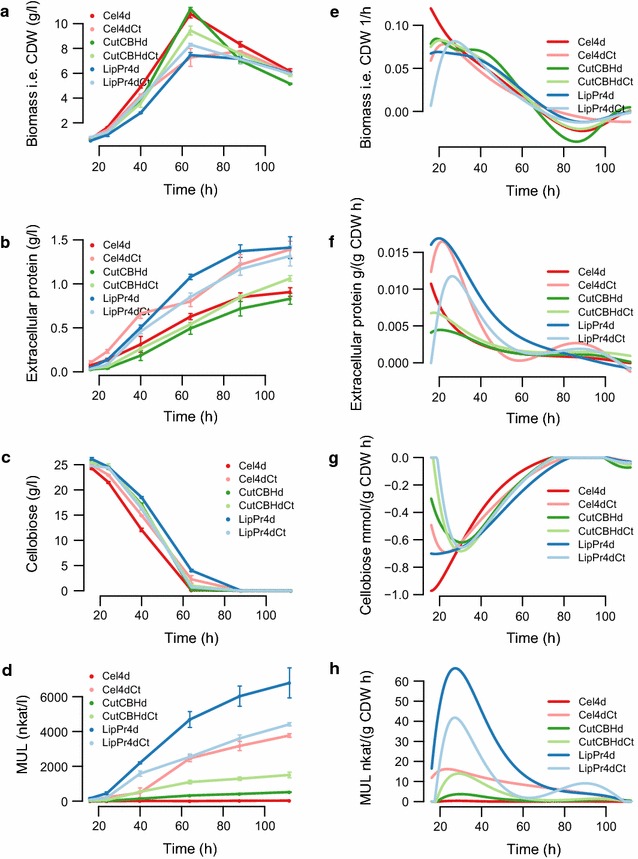
Parameters measured from the bioreactor cultures. The volumetric amount of fungal biomass, extracellular protein, cellobiose and activity against MUL substrate is shown in the panels on the* left* (**a**–**d**) and the corresponding specific rates (per biomass amount and time) in the panels on the* right* (**e**–**h**). The* error bars* indicate the standard error of the mean (SEM). Futher parameters are shown in Fig. 2

### Bioreactor batch fermentations

**Fig. 2:**
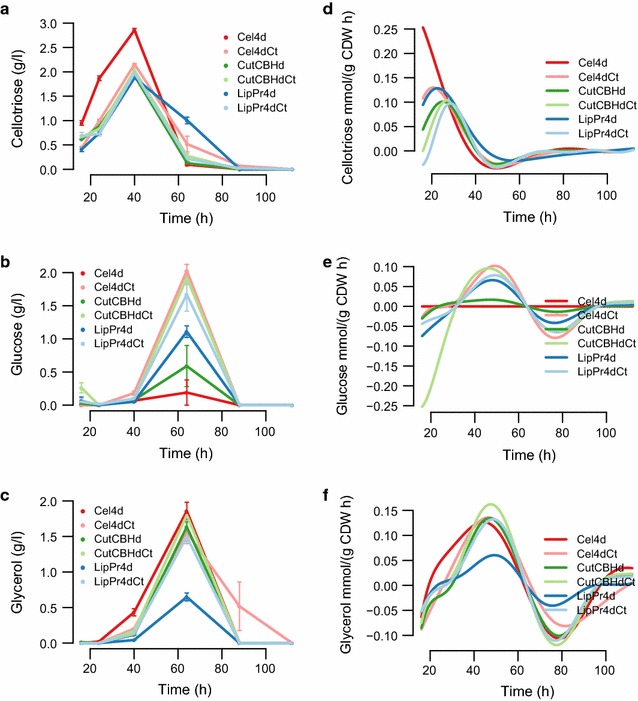
Further bioreactor batch cultivation parameters. The volumetric amount of cellotriose-like compound, glucose and glycerol shown in the panels on the* left* (**a**–**c**) and the corresponding specific rates (per biomass amount and time) in the* panels* on the* right* (**d**–**f**). The exact identity of the cellotriose-like (**a**, **d**) compound is not known. The* error bars* indicate the standard error of the mean (SEM)

The six strains (Table 1) were cultivated in bioreactors each as triplicate. Minimal medium with defined composition was used in the cultivation to enable accurate measurement of the nutrients taken up and the compounds produced as needed for stoichiometric modelling. Cellobiose was used as an inducing carbon source [9] and ammonium sulfate as the source of nitrogen in the cultures. The cultures were extensively monitored both off- and on-line (Figs. 1, 2). CO$_{2}$ and O$_{2}$ were measured on-line and the cultures were sampled at 16, 24, 40, 64, 88 and 112  h for analysis of biomass, extracellular protein, amino acid composition (only at 24, 40 and 64 h) and a multitude of sugars, alcohols and carboxylic acids.

**Table 2:** Counts of genes significantly correlated with various cultivation parameters

Cultivation parameter	Count of correlated genes
Biomass i.e. CDW g/l	1666
Cellobiose g/l/CDW	1633
Glycerol g/l/CDW	1612
OUR mol/(g CDW h)	1132
CER mol/(g CDW h)	1060
Cellotriose g/l/CDW	787
Extracellular protein g/(g CDW h)	272
Cellotriose mmol/(g CDW h)	184
Glycerol mmol/(g CDW h)	149
MUL nkat/(g CDW h)	30
Biomass i.e. CDW 1/h	25
Glucose g/l/CDW	13
Glucose mmol/(g CDW h)	12
MUL nkat/l/CDW	9
Extracellular protein g/l/CDW	3
Ethanol g/l/CDW	0
Lipase g/l/CDW	0

In order to estimate the consumption and production rates, a heteroscedastic Gaussian processes [38] was used to model the rates on the measured data (see "Methods" section for details). The raw cultivation data is presented in Additional file 2: Table S1 and data averaged over triplicates and CDW (cell dry weight) normalised in Additional file 2: Table S2.

The In order to dissect the effect of protein production load, variation in the specific extracellular protein production rate (*g*/*gCDWh*) in the dataset is of importance. The specific production rate of extracellular protein (per the amount fungal biomass) (Fig. 1f) was highest at the early stages of growth around 24  h. The difference in accumulation of total extracellular protein between the strains starts to increase from 24  h onwards, being the largest at 112  h (Fig. 1b). Accordingly the highest variation in the specific production rate of extracellular protein (Fig. 1f) was detected at the 24  h time point. At that time the strains (**Cel4dCt**, **LipPr4d** and **LipPr4dCt**) exhibited a high protein production rate and the strains (**Cel4d**, **CutCBHd** and **CutCBHdCt**) exhibited a low production rate.

Accumulation of cellulase activity, measured as activity against MUL substrate, paralleled the accumulation of total extracellular protein and was the highest in lipase producing strain **LipPr4d**. The detected difference in extracellular protein accumulation appears as a trade-off between biomass accumulation (Fig. 1a) and protein production (Fig. 1b). The strains producing extracellular proteins the most (**Cel4dCt**, **LipPr4d** and **LipPr4dCt**) reached much lower level of biomass during the cultivation as compared to the strains producing less extracellular protein. After 60  h biomass starts to degrade but extracellular protein accumulates still. The increase in the total extracellular level at these late stages of cultivation could be due to either actual production or release of the protein from the lysed cells. The produced lipase forms close to 20 % of total extracellular protein at 40  h but is completely degraded soon after that (Additional file 1: Figure S1). Production of cutinase activity by the strain **CutCBHd** was not measurable.

Apart from the main carbon source also other small molecules were detected by HPLC (and verified as necessary with other methods) in the growth media. In the HPLC analysis, a compound eluting similarly to the cellotriose standard was detected. A further LC-MS analysis showed that the compound is cellotriose-like trisaccharide but the precise structure could not be assigned based on mass spectra. The cellotriose-like compound started to accumulate at 16  h and is completely consumed at 88  h (Fig. 2a, d). Strain **Cel4d** lacking the main cellulases accumulated uniquely large amounts of the cellotriose-like compound. The specific rate of the cellotriose-like compound (mmol/l/gCDW h) paralleled closely the specific growth rate (1/h), however it was consumed faster than the growth decelerated. Glucose started to accumulate at the same time point (40 h) when the level of the cellotriose-like compound reached its maximum and started to decline at 64  h. Glucose was completely consumed at 88  h (Fig. 2b, e). Glycerol accumulation paralleled closely the accumulation of glucose (Fig. 2c, f). Uniquely, the best protein producer strain, in terms of accumulation and highest detected specific protein production rate **LipPr4d**, produced the least amount of glycerol. In addition, accumulation of ethanol was detected at 16 and 24  h in the **CutCBHdCt** and **CutCBHd** strains (Additional file 1: Figure S2). Although ethanol was detectable in all three repeats of **CutCBHdCt** at 24  h the variation of the measurement value is quite large possibly due to evaporation.

**Fig. 3:**
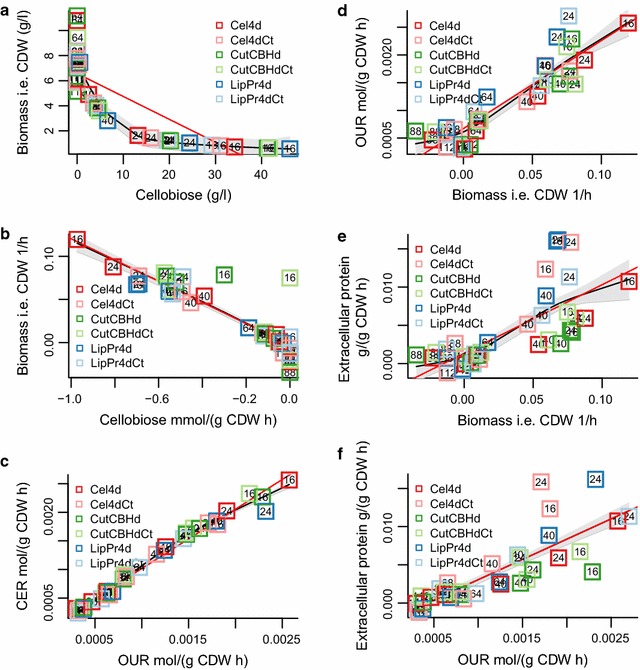
Dependencies between key cultivation parameters. Each number signifies the sampling time (h) during cultivations of a strain. Strains are specified with *colored boxes*. The *red line* is a linear regression model and the *black line* a generalized additive model, surrounded by a *grey region* of one standard deviation. Panels (**a**–**f**) show dependencies between **a** biomass (g/l) and cellobiose (g/l), **b** biomass (1/h) and cellobiose [mmol/(g CDW h)], **c** CER [mol/(g CDW h)] and OUR [mol/(g CDW h)], **d** OUR [mol/(g CDW h)] and biomass (1/h), **e** extracellular protein [g/(g CDW h)] and biomass (1/h) and **f** extracellular protein [g/(g CDW h] and OUR [mol/(g CDW h)]

In order to better understand the dependencies between the cultivation variables, we calculated the correlation between selected pairs of variables (Fig. 3). Biomass concentration correlated strongly with carbon source concentration (Fig. 3a), but comparison of specific rates (Fig. 3b) reveals that at 16  h **CutCBHdCt** and **CutCBHd** strains take up the carbon source at a higher rate than would be expected by their growth rate. Specific CO$_{2}$ exchange rate (CER) and O$_{2}$ consumption rate (OUR) correlated strongly with each other, with the exception that at the highest point of specific extracellular protein production rate in the whole experiment (**LipPr4d** at 24  h) CO$_{2}$ was produced at a slightly smaller rate than expected based on OUR. Unfortunately there is no OUR or CER data for the **LipPr4d** 16  h sample. Specific extracellular protein production rate, specific OUR and specific growth rate all correlated significantly linearly (OUR and growth rate, $r = 0.81, p < 2e{-}13$ Fig. 3d; protein and growth rate, $r = 0.51, p < 6e{-}7$, Fig. 3e; protein and OUR, $r = 0.66, p < 3e{-}9$ Fig. 3f). The five sampling points with the highest specific extracellular protein production rate in descending order were **LipPr4d** 24 and 16  h, **Cel4dCt** 24 and 16  h and **LipPr4dCt** 24  h. Excluding lacking data for **LipPr4d** 16  h, these sampling points had a higher specific extracellular protein production rate than expected by specific growth rate or by specific OUR. An opposite phenomena was visible for the 16, 24 and 40 h sampling points of **CutCBHd** and **CutCBHdCt** and **Cel4dCt** strains.

In addition to the extracellular variables, we measured the concentration of intracellular free amino acids from the 24, 40 and 64 h sampling time points (Additional file 1: Figure S3). Overall, they exhibited a decreasing trend during cultivation. Exceptions to this trend were often associated with larger deviation between repeats or with small concentrations. Over 1000 ${\upmu}\rm{ mol/g CDW}$ concentrations were detected for glutamine, glutamate, arginine and alanine and all of these exhibit a systematic downward trend. The **LipPr4d** strain had a higher alanine concentration than its control strain **LipPr4dCt** and all other strains in the three measured sampling points. Otherwise, the variability in amino acid concentrations between strains was best explained not by differential protein production or growth rates, as was the case for extracellular variables, but rather by strain background.

### Transcription profiling with RNA sequencing

In order to access the intracellular genome wide effect of variable protein production load, we carried out Illumina 100 bp pair-end RNA sequencing from the cultivation time points of 16, 24, 40 and 64  h for all the strains. The read data has been deposited to NCBI-SRA [39] with BioProject number PRJNA293671 and results of subsequent genome wide analysis at gene level are presented in Additional file 2: Table S5. The median FPKM (fragments per kilobase of exon per million fragments) over the whole experiment was 20. 6332 genes of our gene list of 9441 genes reached this expression in at least one sample and only 34 genes had a FPKM of zero in all the samples.

#### Correlation of gene expression with cultivation parameters

In order to count correlations between cultivation parameters and gene expression values, we normalized concentration parameters (rate parameters already take biomass into account) with biomass i.e. CDW (g/l). This was to take into account the fact that the compounds are produced by a certain amount of cells. We did not apply this for the carbon source cellobiose, but of the genes correlating with cellobiose concentration with biomass normalization, 85 % also correlated without the normalisation. After this normalization OUR (mol/gCDW h), CER (mol/gCDW h), cellotriose concentration (g/l/CDW), cellobiose concentration (g/l/CDW), glycerol concentration (g/l/CDW) have a similar decreasing shape, which correlates strongly negatively with the biomass i.e. CDW (g/l) (Fig. 1a).

As gene expression data we used rlog values from DESeq2 [40] (see below “Analysis of significantly changing genes”). The rlog behaves similarly to log2 transformation but shrinks variability for genes with low read counts to control measurement error in them. We averaged the rlog gene expression data over the triplicate cultivations to derive 24 data points (six strains and fourtime points) for each gene. For each gene we calculated its correlation to all the cultivation parameters individually. For every parameter we then filtered a list of correlated genes with $FDR \le 0.00005$ which corresponds to approximately to $absolute(r) \ge 0.79$ (Table 2). Additional file 1: Figure S5 shows a sample of scatter plots of individual gene—arameter pairs at this FDR cut-off. Parameters mentioned above which correlated with biomass i.e. CDW (g/l) have from 800–1600 correlated genes in contrast to growth rate 1/h which has only 25 correlated genes. Extracellular protein rate (g/gCDW h) has 272 correlated genes in contrast to extracellular protein concentration (g/l/CDW) which has 3 correlated genes.

**Table 3:** Enrichment of annotation terms in groups of genes significantly correlated cultivation parameters

Type	Cultivation parameter	Term	Count of genes	*P* value
GO	Biomass i.e. CDW (g/l)	GO:0009987 cellular process BP	426	0.00006
GO	Biomass i.e. CDW (g/l)	GO:0071704 organic substance metabolic process BP	423	0.00137
GO	Biomass i.e. CDW (g/l)	GO:0044238 primary metabolic process BP	415	0.00115
GO	Biomass i.e. CDW (g/l)	GO:0044237 cellular metabolic process BP	387	0.00000
GO	Biomass i.e. CDW (g/l)	GO:0044260 cellular macromolecule metabolic process BP	276	0.00082
GO	Biomass i.e. CDW (g/l)	GO:0009058 biosynthetic process BP	255	0.00000
GO	Biomass i.e. CDW (g/l)	GO:1901576 organic substance biosynthetic process BP	247	0.00000
GO	Biomass i.e. CDW (g/l)	GO:0044249 cellular biosynthetic process BP	246	0.00000
GO	Biomass i.e. CDW (g/l)	GO:0019538 protein metabolic process BP	224	0.00000
GO	Biomass i.e. CDW (g/l)	GO:0044267 cellular protein metabolic process BP	193	0.00000
Class	Cellotriose mmol/(g CDW h)	Transport	17	0.00034
GO	Cellotriose mmol/(g CDW h)	GO:0071702 organic substance transport BP	13	0.00561
Class	Cellotriose mmol/(g CDW h)	Glycoside hydrolase	10	0.00235
Class	Cellotriose mmol/(g CDW h)	Major facilitator superfamily	9	0.00064
Cazy	Cellotriose mmol/(g CDW h)	Glycoside hydrolase	9	0.00204
GO	Cellotriose mmol/(g CDW h)	GO:0008643 carbohydrate transport BP	8	0.00008
Class	Cellotriose mmol/(g CDW h)	Cell cycle	6	0.00514
Class	Cellotriose mmol/(g CDW h)	Cell growth and death	6	0.00595
Interpro	Cellotriose mmol/(g CDW h)	IPR002198: Short-chain dehydrogenase/reductase SDR	6	0.00784
Interpro	Cellotriose mmol/(g CDW h)	IPR010730: Heterokaryon incompatibility	5	0.00014
Class	Extracellular protein g/(g CDW h)	Transport	20	0.00226
GO	Extracellular protein g/(g CDW h)	GO:0005975 carbohydrate metabolic process BP	18	0.00018
Interpro	Extracellular protein g/(g CDW h)	IPR011701: Major facilitator superfamily	12	0.00083
Class	Extracellular protein g/(g CDW h)	Amino acid metabolism	10	0.00171
Class	Extracellular protein g/(g CDW h)	Secreted	10	0.00313
Interpro	Extracellular protein g/(g CDW h)	IPR010730: Heterokaryon incompatibility	6	0.00015
Interpro	Extracellular protein g/(g CDW h)	IPR003663: Sugar/inositol transporter	5	0.00189
Interpro	Extracellular protein g/(g CDW h)	IPR005828: Major facilitator, sugar transporter-like	5	0.00551
Class	Glycerol mmol/(g CDW h)	Protein transport	7	0.00224
Class	Glycerol mmol/(g CDW h)	Cell signalling	5	0.00140

Subsequently for each list of correlated genes we carried out an enrichement analysis (Table 3; Additional file 2: Table S3 for further details). Glycerol (g/l/CDW), biomass i.e. CDW (g/l), cellobiose (g/l/CDW), OUR (mol/gCDW h), CER (mol/gCDW h), cellotriose (g/l/CDW) correlated genes are essentially enriched with similar categories, hence we show only enrichment of genes correlated with biomass i.e. CDW (g/l) in Table 3. These genes belong to primary cellular bioprocesses like primary metabolism, biosynthesis, gene expression and translation. Their expression correlates negatively with biomass and positively with the other cultivation parameters with similar shape i.e. they have a decreasing trend throughout the experiment.

Cellotriose rate mmol/(gCDW h) (Fig. 2d) and extracellular protein rate g/gCDW h (Fig. 1f) have similar shapes and hence share enrichment categories of correlated genes such as transport i.e. major facilitator superfamily (MFS) and other transporters, short-chain dehydrogenases, MFS transporters specifically and various secreted proteins. The class “Secreted” refers to genes of which nothing else is known. The GO:0005975 “carbohydrate metabolic process” refers in this case to various glycoside hydrolases which expression follows protein production. Genes correlated with extracellular protein rate *g*/*gCDWh* are also enriched in genes related to amino acid metabolism. These include cysteine metabolism genes (120176, EC:1.13.11.20, cysteine dioxygenase; 56350, EC: 2.5.1.47, cysteine synthase; 68036, EC:2.5.1.48, cystathionine gamma-synthase; 3823, EC: 2.1.1.14 methionine synthase; shown also on Fig. 9) and tryptophan biosynthesis genes (67003, ECs: 4.1.3.27, 4.1.1.48, indole-3-glycerol-phosphate synthase; 75414, EC: 2.4.2.18, anthranilate phosphoribosyltransferase). These two enzymes catalyse three of the five reactions from chorismate to tryptophan.

Genes correlated to glycerol rate *mmol*/*gCDWh* are enriched in genes related to protein transport. In particular these genes include *snc1* (53601), *ftt1* (121028), YPT7 (60331), SFB3 (77570), SNF7 (81214) and MNN4 (67907). The enriched cell signaling related genes include various GTPase or related genes. This set of genes overlaps significantly with genes expressed at higher level in strains producing high amounts of protein at 64  h than in low producing strains (Fig. 5b).

#### Analysis of significantly changing genes

Based on differences seen in the fermentation parameter (Figs. 1, 2, 3) data, we chose to test time point wise differences between strains producing high (**LipPr4d**, **LipPr4dCt** and **Cel4dCt**) and low (**Cel4d**, **CutCBHd** and **CutCBHdCt**) levels of protein. We then applied DESeq2 [40] to the RNA-seq read count data to detect significantly changing genes in response to differential protein production load. All together 1081 genes with a false discovery rate $\le 0.0001\,\%$ and minimum log2 fold change of 0.5 were detected as differentially regulated.

**Fig. 4:**
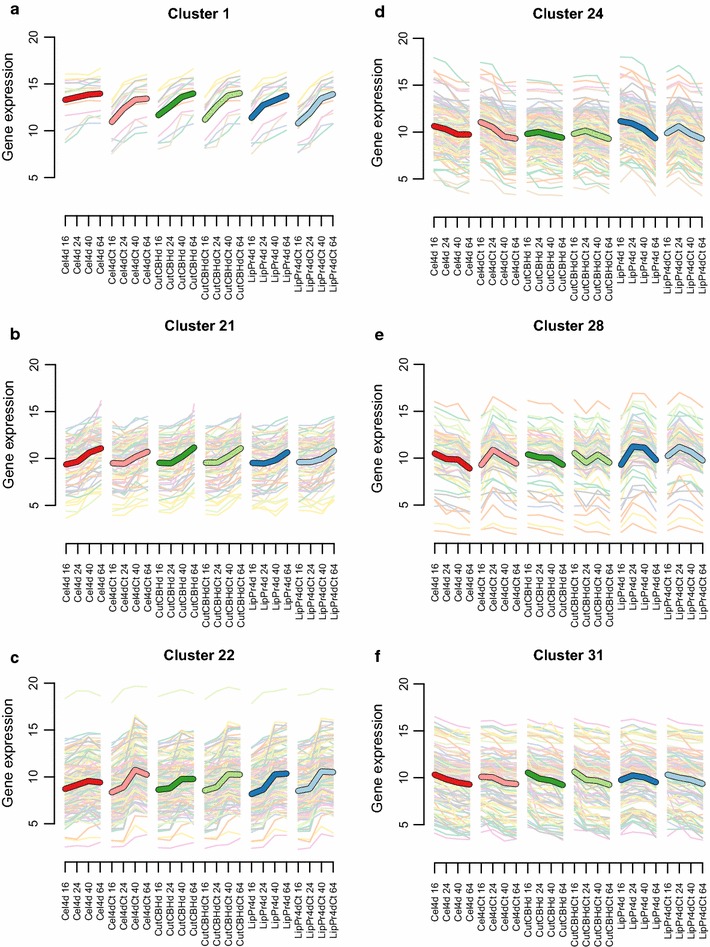
Selected gene expression clusters. On y-axes the average gene expression level expressed as rlog2 which is log2 like transformation of normalized counts calculated by DESeq2 [40].* Thick line* is cluster average and* thin lines* individual genes. On x-axes time points (16–64 h) from the cultivations of the six strains

**Table 4:** Enrichment of annotation terms in gene expression clusters

Type	Cluster	Term	Count of genes	*P* value
Interpro	1	IPR007274: Ctr copper transporter	4	0.00000
GO	1	GO:0006825 copper ion transport	4	0.00000
Class	1	Transport	4	0.00015
Class	5	Mitochondrial	6	0.00800
Class	5	Transmembrane	4	0.00208
Class	20	Metabolism	6	0.00511
Class	20	Transport	4	0.00544
Class	21	Amino acid metabolism	5	0.00019
Class	21	Carbohydrate metabolism	4	0.00050
Class	22	Glycoside hydrolase	9	0.00070
Class	22	Unknown	8	0.00070
Cazy	22	Glycosyltransferase	5	0.00159
GO	22	GO:0006629 lipid metabolic process lipid metabolic process	5	0.00830
Class	24	Metabolism	18	0.00141
GO	24	GO:0005975 carbohydrate metabolic process	11	0.00146
Class	24	Protein transport	8	0.00011
Class	24	Amino acid metabolism	5	0.00299
Class	24	Nucleasome	4	0.00016
Interpro	24	IPR013149: Alcohol dehydrogenase, C-terminal	4	0.00038
Interpro	24	IPR013154: Alcohol dehydrogenase, N-terminal	4	0.00046
GO	26	GO:0006629 lipid metabolic process	4	0.00143
GO	28	GO:0006810 transport	17	0.00003
GO	28	GO:0051234 establishment of localization	17	0.00003
Class	28	Metabolism	10	0.00937
GO	28	GO:0006820 anion transport	9	0.00000
GO	28	GO:0006865 amino acid transport	7	0.00000
GO	28	GO:0050790 regulation of catalytic activity	5	0.00028
Class	28	Amino acid metabolism	5	0.00036
Class	28	Major facilitator superfamily	5	0.00058
GO	28	GO:0051341 regulation of oxidoreductase activity	4	0.00067
Class	29	Transport	6	0.00040
GO	31	GO:0009058 biosynthetic process	27	0.00392
GO	31	GO:0044249 cellular biosynthetic process	26	0.00398
GO	31	GO:1901564 organonitrogen compound metabolic process	16	0.00008
GO	31	GO:0044281 small molecule metabolic process	15	0.00102
GO	31	GO:1901566 organonitrogen compound biosynthetic process	13	0.00002
GO	31	GO:0006520 cellular amino acid metabolic process	12	0.00005
GO	31	GO:0008652 cellular amino acid biosynthetic process	9	0.00001
Class	31	Translation	7	0.00151
Class	31	Ribosome	6	0.00085
GO	31	GO:1901605 alpha-amino acid metabolic process	6	0.00238
Class	31	Transcription	6	0.00519
GO	31	GO:1901607 alpha-amino acid biosynthetic process	5	0.00273
Class	31	Transcription factor	5	0.00983
Interpro	31	IPR012335: Thioredoxin_fold	4	0.00072
GO	31	GO:0006865 amino acid transport	4	0.00612
GO	31	GO:0015711 organic anion transport	4	0.00612
GO	31	GO:0015849 organic acid transport	4	0.00612
GO	31	GO:0046942 carboxylic acid transport	4	0.00612

**Fig. 5:**
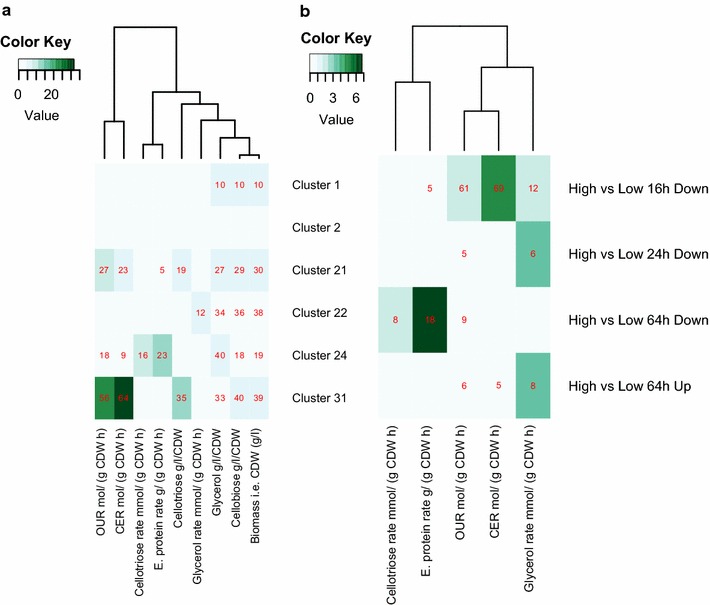
Overlaps of groups of genes. Overlap of groups of genes correlated significantly with a cultivation parameter (Table 3) (**a**) with genes in expression clusters and (** b**) with groups of differentially expressed genes. Heatmap coloring shows the negative log* p* value of overlap with a cut-off of two i.e. only overlaps with $p \le 0.01$ are shown.* Red cell* notes show the actual overlapping count of genes. In (**b**) 'High’ refers to strain producing protein well (Cel4dCt, LipPr4d and LipPr4dCt) and 'Low’ to strains producing less protein (Cel4d, CutCBHd and CutCBdCt). 'Down’ signifies the direction of regulation i.e. the group of genes is expressed at significantly lower level in ’High’ strains, than in 'Low’ strains. ’16 h’ specifies the time point i.e. sample taken at 16  h

In order to dissect the different gene expression patterns of these 1081 genes, we clustered them based on the rlog (regularized logarithm transformation) gene expression values from DESeq2 [40] into clusters of co-regulated genes with Bayesin hierarchical clustering [41]. All together we retrieved 32 clusters (Fig. 4 shows a subset, while all are shown in Additional file 1: Figure S4). We then carried out enrichment analysis for the clusters (Table 4 and Additional file 2: Table S4 for further details). Furthermore, we calculated the overlap of groups of genes correlated significantly with a cultivation parameter (Table 3) with genes in expression clusters (Fig. 5a) and with groups of significantly differentially expressed genes (Fig. 5b) and the overlap between clusters and significantly differentially expressed genes (Additional file 1: Figure S6).

**Fig. 6:**
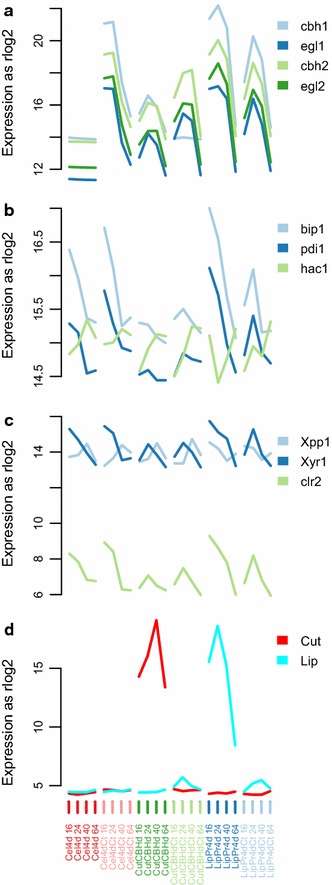
Expression levels of individual genes. On* y-axis* the average gene expression level expressed as rlog2 which is log2 like transformation of normalized counts calculated by DESeq2 [40]. On* x-axis* time points (16–64 h) from the cultivations of the six strains.** a** main cellulases of *T. reesei*.** b** Central genes of protein secretion.** c** Selected differentially expressed regulators.** d** Heterologous product genes cutinase (*Cut*) and lipase (*Lip*). Main cellulase signal in cellulase deletion strain **Cel4d** is a technical artefact created by the DESeq2 process as exemplified by its lack of variation in **Cel4d** samples

In **cluster 24** gene expression peaks at 16  h (Fig. 4d). These genes are significantly enriched in genes significantly correlated with extracellular protein rate (g/gCDW h) and cellotriose rate mmol/(gCDW h) (Fig. 5a). These genes tend to be at higher level in 16 and 24 h time points in strains producing high amounts protein than in low producing strains (Fig. 4d). The cluster is also enriched in genes that are at significantly lower level at high producing strains at 16 and 64  h than in low producing strains (Additional file 1: Figure S6). Like genes significantly correlated with glycerol rate mmol/gCDW h cluster 24 genes are enriched in genes related to protein transport and folding process (Table 4). However, these are not vesicle but, ER related (*cnx1* 73678, *prpA* 28928, PMR1 *120627*, ORP150 35465, SPC12 121948, SPC2 5066, FKBP 33895, *pdi1* 122415 and *bip1* 122920, see Fig. 6 for *pdi1* and *bip1*).

The induction of ER related secretion factors raises the question of whether a UPR response is present in the cultivations. We looked for evidence of *hac1* splicing in the RNAseq read alignment data and found none.

In cluster 24 there is also an enrichment of various unclassified metabolic enzymes, which include two cytochrome P450s, two short-chain dehydrogenase and a polyketide synthase genes i.e. possibly related to secondary metabolism. More specifically there are five amino acid metabolism related genes including CHA1 53091 EC: 4.3.1.17 (Fig. 9) and ARG1 82619 EC: 6.3.4.5. The nucleosome related genes include 3 GCN5-related N-acetyltransferases and a BTB/POZ domain protein. The carbohydrate metabolism genes include mainly glycoside hydrolases such as two mannosidases, two galactosidases and three glucosidases. Finally, cluster 24 contains *ace3* 77513 [15], homologue of *Neurospora crassaclr2* [15, 23] 26163 and two other IPR001138: Fungal transcriptional regulatory protein -family proteins (108381 and 70351 [15]).

**Cluster 28** genes peak at 24  h in high producing strains. These genes are not enriched in any set of genes correlating significantly with some cultivation parameter (Fig. 5). They are enriched in genes that are at significantly lower level at high producing strains at 16  h than in low producing strains and at higher level in 24  h in high strains (Additional file 1: Figure S6). Various transporters are significantly enriched in this set of genes in particular major facilitator family, but also amino acid permeases, for example GAP1 121139. There is also an enrichment of various unclassified metabolic enzymes that might have role in secondary metabolism, but which also include 3 taurine oxidoreductases. More specifically there are amino acid metabolism related genes including SER1 121345 EC:2.6.1.52 , putative cysteine synthase 76018 EC: 2.5.1.27 (Fig. 9), sulfinoalanine decarboxylase 121664 EC: 4.1.1.29 and LYS21 123471 EC: 2.3.3.14.

**Cluster 22** genes peaks at 40  h (Fig. 4). These genes are significantly enriched in genes significantly correlated with glycerol rate *mmol*/*gCDWh* (Fig. 5a). They are also enriched in genes that are at significantly lower level at high producing strains at 16  h than in low producing strains and at higher level at 64  h in high strains (Additional file 1: Figure S6). Lipid related genes are significantly enriched among these genes. They include for example 3 putatively secreted triglyceride lipases. Also glycoside hydrolases are enriched in cluster 22 but unlike glycoside hydrolases in cluster 24 these glycoside hydrolases are related to fungal cell wall. They include a chitinase, 2 alpha-1,2-mannosidases, a alpha-1,6-mannanase and 2 endo-beta-glucanases. Glycosyl transferases possibly related to fungal cell membrane and wall are also enriched. Altogether 31 of the 65 genes in cluster 22 are predicted to be targeted to the secretion pathway.

Genes significantly correlated with glycerol rate mmol/gCDW h are similarly regulated as genes of cluster 22. However, cluster 22 does not contain the vesicle related genes of protein transport reported above. This is because expression of these genes is not significantly different between high and low producing strains.

**Clusters 1, 21 and 31** represent the general growth stage dependent, biomass correlated, responses with genes of cluster 1 going up while the cultivation progresses and genes of cluster 21 and 31 going down (Fig. 4). cluster 31 mainly includes the protein translation process (Table 4). cluster 21 is enriched in amino acid metabolism related genes (Table 4; Fig. 9). These include PUT1 54564 EC: 1.5.5.2, CAR1 123738 EC: 3.5.3.1, PDA1 56726 EC: 1.2.4.4 and PDB1 122745 EC: 1.2.4.4, that are all involved in amino acid degradation and use as source of carbon or nitrogen. cluster 1 is enriched in copper transporters.

**Glycoside hydrolases** gene expression was found to be correlated (above) with cellotriose rate mmol/gCDW h (Fig. 2d) and extracellular protein rate g/gCDW h (Fig. 1f). Expression of the four main cellulases, along with other proteins of interest, is shown in Fig. 6. The amount of extracellular protein produced in strain **Cel4dCt**, with the four main cellulases deleted, is 65  % of the amount produced by its control strain **Cel4dCt** with intact main cellulases.

**Fig. 7:**
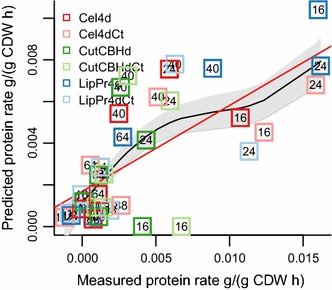
Correlation of predicted and measured specific protein production rate. Each number signifies the sampling time (h) during cultivations of a strain. Strains are specified with* colored boxes*. The* red line* is a linear regression model and the* black line* a generalized additive model, surrounded by a* grey region* of one standard deviation

In order to understand what proteins are produced instead of the main cellulases we inspected gene expression regulation of CAZY and related genes known to be highly produced based on proteomics [6] (Additional file 1: Figure S7). Genes *egl4*, *xyn2*, *egl3xyn1*, *bxl1*, *cel74a* and *glr1* were found to be at higher level in the deletion strain **Cel4d** than its control strain **Cel4dCt**.

#### DNA motif discovery from promoter regions of gene expression clusters

In order to find regulatory factors for the gene expression responses detected by analysis of significantly changing genes, we analyzed promoter sequences of genes grouped by the gene expression clusters with FIRE [42]. Three different motifs were discovered (Additional file 1: Figure S8) 1: (G/T)ACGTCA(C/T), 2: (A/C/T)(A/T)TTAG(C/G)C(A/G/T) and 3: (A/G/T)TA(C/G)GC(A/T)A.

Motif 1 (ACGTCAT) is over represented in promoters of genes in gene expression cluster 5 (Additional file 1: Figure S4) and it has been found previously from *Fusarium graminearum* [43], *Saccharomyces cerevisiae* [44] and *Schizosaccharomyces pombe* [45] and it is involved in regulation of the environmental stress response. cluster 5’s genes peak in 40 h time point, but do not show a response in lipase strains (**LipPr4d** and **LipPr4dCt**).

Motif 2 (TTTAGCC) is typically found on the opposite strand (Additional file 1: Figure S7) i.e. its complement is GGCTAAAA which is the XlnR motif in *Aspergillus* species [46–48] and the motif of the orthologous *Xyr1* in *T. reesei* [20, 49, 50]. It is over represented in clusters 24 and 28 and under represented in cluster 5. It is found in 38  % of the 119 genes in cluster 24 and in 43  % of the 65 genes in cluster 28. In these two clusters it is found in 10 transporter genes, three amino acid metabolism genes (including ARG1 82619 EC: 6.3.4.5), in five glycoside hydrolases (including *xyn2* 123818), in three genes related to protein folding (ORP150 35465, FKBP 33895 and *pdi1* 122415) and two secondary metabolism related genes (including the nonribosomal peptide synthase gene 60458).

Motif 3 (TAGGCAA) is over represented in cluster 17 and under represented in cluster 5 and 31. Cluster 17 contains only 12 genes. These show a higher plateau at 24 and 40  h in high producing strains (**LipPr4d**, **LipPr4dCt** and **Cel4dCt**) in their gene expression (Additional file 1: Figure S4). The cluster contains 2 IPR003819: “Taurine catabolism dioxygenase TauD/TfdA”—family proteins that are related to sulphur metabolism.

#### Comparison to previous *T. reesei* transcriptomics work

In order to see whether the gene expression responses we detect are specific for this experiment or universally seen in *T. reesei* batch cultivations we looked for significant overlap of our gene expression clusters between gene regulation modules from [51]. These modules were predicted with Genomatica [52] with improved post processing. The data includes 105 gene expression microarray samples (each an average of three repeats) from [6, 12, 15] and unpublished data. Out of all the gene expression clusters, genes of clusters 5, 22, 28 and 31 were found to significantly ($p<0.00001$) overlap with genes of some specific module with 10, 39, 24 and 10 % of their genes, respectively, found in the most overlapping module.

**Table 5:** Genes with over 0.8 correlation to specific protein production rate in this publication and in [108]

JGIno	Class	Cluster	Extension	SGD best hit	Description	Location
41248	Carbohydrate esterase		Family 3		Candidate acetyl xylan esterase (EC.3.1.1.72)	
73005	Glycoside hydrolase		Family 79		Candidate beta-glucuronidase (EC.3.2.1.-, 3.2.1.31)	Secreted
69840	Metabolism		Fatty acid	SPS19	Peroxisomal 2,4-dienoyl-CoA reductase, auxiliary enzyme of fatty acid beta-oxidation	
23223	Metabolism		Amino acid	GLY1	Threonine aldolase, catalyzes the cleavage of l-allo-threonine and l-threonine to glycine. Involved in glycine biosynthesis	
107218	Metabolism		Carbohydrate		Carbohydrate kinase	
104071	Regulatory functions				Pannzer: Zn(2)-C6 fungal-type DNA-binding domain (IPR001138)	
75923	Regulatory functions		Transcription factor		Pannzer: Fungal specific transcription factor domain (IPR021858)	
123668	Regulatory functions		Histone modification protein		GCN5-related N-acetyltransferases	
60945	Transport		Major Facilitator Superfamily	ITR1	Myo-inositol transporter with strong similarity to the minor myo-inositol transporter Itr2p. Among top sophorose induced genes in [17]	
121608	Transport	24	Major Facilitator Superfamily	TNA1	High affinity nicotinic acid plasma membrane permease, responsible for uptake of low levels of nicotinic acid	
78833	Transport				Pannzer: Fucose permease. Among top induced transporters on cellulose and sophorose in [17]	
66370	RNA		Exonuclease			
67541	Transport		MFS, Sugar	MAL31	Maltose permease, high-affinity maltose transporter (alpha-glucoside transporter)	
70998	Transport		Amino acid permease	HNM1	Choline transporter (permease) that also controls the uptake of nitrogen mustard	
111495	Unknown					Secreted
104227	Unknown					Secreted
105444	Unknown					Secreted
103048	Unknown	24				

More specifically, we wanted to know if the gene expression responses in batch cultivation correlate with extracellular protein production rate (g/gCDW h) similarly as they correlate in a chemostat cultivations. To this end we compared the correlation of each gene’s expression to extracellular protein production rate (g/gCDW h) in this work and in [6] (Additional file 1: Figure S9). We found no over all correlation. However, for example *pdi1*’s correlation to protein production rate in this work is 0.69 and in [6] it is 0.8. The respective numbers for *bip1* are 0.66 and −0.31. The genes that have correlation of at least 0.8 in both works include two genes of cluster 24, transcription factors and transporters, but no secretion factors (Table 5).

### Flux balance analysis with a genome wide stoichiometric model

In order to understand how the variable protein production load reflects on intracellular metabolism, we carried out flux balance analysis [53, 54]. As a model we used a CoReCo [55] created *T. reesei* genome wide metabolic model with an experimentally defined biomass function (manuscript under preparation, Biomodels MODEL1604140000). The model was constrained by the measured growth rate and uptake and secretion rates determined from cultivation media metabolite accumulation data (Figs. 1, 2). For each time point of each cultivation we created a specific model to simulate reaction fluxes in those specific conditions. To estimate the performance of our modelling we predicted extracellular protein production rate in each sample point (Fig. 7). The linear correlation between predicted and measured protein production rate g/gCDW h is $r=0.51$ with a $p<0.0000006$.

**Fig. 8:**
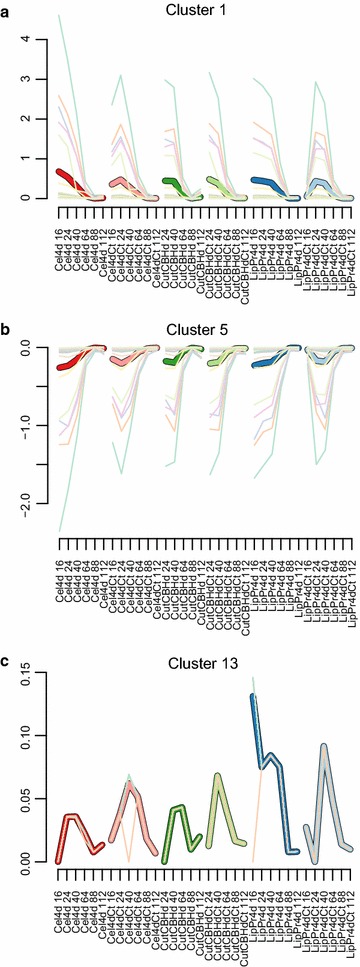
Selected flux clusters** a** Cluster 3;** b** Cluster 4;** c** Cluster 13. On* y-axis* flux. On *x-axis* time points (16–64 h) from the cultivations of the six strains.* Thick line* is cluster average and* thin lines* individual reactions

We then filtered the simulated flux distributions with flux variability analysis (FVA) [56] to remove undeterminable fluxes and combined the remaining fluxes into a single data set. In order to discover major trends in this data set we clustered reactions by their fluxes with Bayesin hierarchical clustering [41] (Fig. 8 shows a subset, while all are shown in Additional file 1: Figure S10) and carried out enrichment analysis for the clusters (Table 6).

Flux clusters 1, 2, 3, 4 and 5 follow closely the growth rate (1/h) with positive (1, 2 ,3 and 4) or negative (5) correlation. After the strain specific peak in growth rate at 16 or 24  h the flux of these reactions decreases. These clusters include reactions for major biosynthesis pathways for nucleotides, amino acids and fatty acids.

**Fig. 9:**
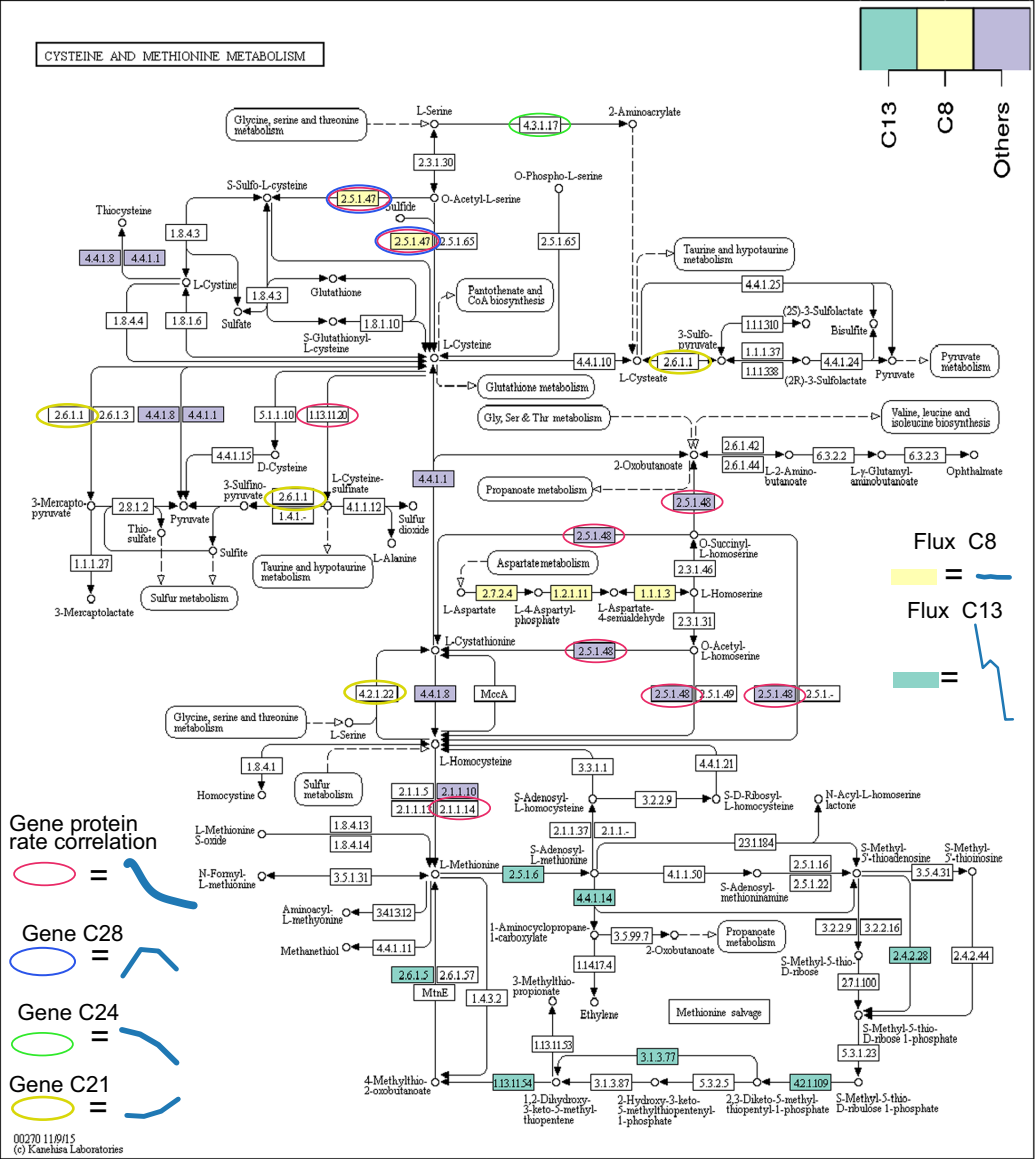
Cysteine and methionine metabolism. Enzymes which reactions belong to flux clusters are colored, for example C13 is flux cluster 13. Enzymes which genes are significantly correlated with protein production rate are encircled in* red*: 120176, EC:1.13.11.20, cysteine dioxygenase; 56350, EC: 2.5.1.47, cysteine synthase; 68036, EC:2.5.1.48, cystathionine gamma-synthase; 3823, EC: 2.1.1.14 methionine synthase. Enzyme(s) found in gene expression cluster 24 is encircled with* green*: 53091 EC: 4.3.1.17, in cluster 28 in* blue*: 76018 EC: 2.5.1.47 and in cluster 21 in* yellow*: 5233 EC: 2.6.1.1, 81089 EC: 4.2.1.22. For each gene expression and flux cluster the profile of strain **LipPr4d** shown. Pathway map from KEGG [107]

Flux cluster 13 displays a markedly different behavior in the high producing strains (**LipPr4d**, **LipPr4dCt** and **Cel4dCt**) in comparison to the low producing strains (**Cel4d**, **CutCBHd** and **CutCBHdCt**). It is enriched in reactions of cysteine and methionine metabolism. Genes responsible for cysteine and methionine metabolism enzymes also uniquely emerge from analysis of the gene expression data (Fig. 9). The reactions of cysteine and methionine metabolism in flux cluster 13 constitute the methionine salvage pathway [57]. This pathway recycles sulphur from 5′-methylthioadenosine and it shows a higher flux in high producing strains particularly in **LipPr4d**. In parallel, the gene expression of enzyme synthesizing cysteine (56350, EC: 2.5.1.47, cysteine synthase, gene expression cluster 28), the major sulphur containing metabolite, correlates significantly with the extracellular protein production rate. In contrast, the flux through this reaction does not follow the extracellular protein production rate nor the gene expression of its enzyme (Fig. 9).

## Discussion

In order to dissect the effect of variable protein production load to the secretory machinery and physiology of *T. reesei* in batch cultivations, sufficient variation in protein production is required. In our data set, a 1.7 times higher protein amount was produced by the highest producing strain **LipPr4d**, i.e. the lipase producer, as compared to the lowest producing strain **CutCBHd**. Together with the above presented results this clearly shows that sufficient variation was reached. The range of protein amount produced is typical for *T. reesei* cultures on minimal medium [58] and the specific protein production rate reaches levels reported for industrial protocols [16, 59]. As a carbon source we selected cellobiose. Cellulose or other more complex lignocellulosic material would have been likely to lead to stronger induction of protein production. However, in order to be able to quantify the carbon source uptake rate to enable stoichiometric modelling, we needed a defined carbon source.

A number of variables, such as growth phase, growth rate or the specific product proteins itself, can confound the effects of the load of protein production on transcriptome and physiology. We try to overcome these problems firstly by including variation of these variables in the data set and secondly by data analysis methods. All of our strains have different specific growth rates and produce a different protein mixture, some with heterologous product proteins and some with different sets of endogenous proteins. Hence, we are able, to a great extent, to control for these factors and detect responses that are not merely results of such confounding factors. On the data analysis side we use a combination of correlation, differential expression and clustering methods to overcome biases in data and shortcomings of individual methods. For example gene expression cluster 5 (Additional file 1: Figure S4) shows a peak of expression at 40  h in all strains except for the lipase (**LipPr4d** and **LipPr4dCt**) strains. The cluster is enriched in genes which are expressed at a significantly lower level in the high producing strains (**Cel4dCt**, **LipP4rd** and **LipPr4dCt**) than in the low producing strains (**Cel4d**, **CutCBHd** and **CutCBHdCt**) at 40  h (Additional file 1: Figure S6). However, the responses of the lipase production strain (**LipPr4dCt**) and cellulase deletion control strain (**Cel4dCt**) (i.e. the strains producing protein best) at 40h are quite opposite even though their protein production rate (Fig. 1f) and amount of protein produced at this time point are almost identical (Fig. 1b). Hence, expression of the genes in cluster 5 is not effected by the protein production load but by some other factors. Without including sufficient variation in the experimental design this response could be easily misinterpreted.

In order to calculate meaningful uptake and secretion rates, i.e. first order derivatives, from the concentration data of batch cultivations, the measurement data has to be either interpolated, smoothed or modeled. Typically this is done for example with higher order polynomials, smoothing splines [60] or a Monod model [61]. We propose to use heteroscedastic Gaussian processes [38] for this task. Gaussian processes infer a distribution of all interpolating functions that match the observation, instead of a single interpolant. The distribution mean characterises the most likely interpolation function, which are shown in Figs. 1 and 2 (left). We employ time-dependent, i.e. heteroscedastic, observation noises that can realistically model the differing observation variances at different measurement times. Finally, we derive the derivative distribution as a second Gaussian process [62], whose mean indicates the most likely uptake and secretion rates over time, which are shown on the right side column of Figs. 1 and 2. At the first and last time point of the time series these rate estimates might slightly suffer from lack of preceding and subsequent data, respectively.

We detected a cellotriose-like compound, glucose, glycerol and ethanol from the growth media. For the moment we can only speculate about the source of these compounds, but clearly, based on their dynamics, they are produced by *T. reesei* cells or by secreted enzymes. To our knowledge this is the first time that such metabolism has been reported for *T. reesei*. The cellotriose-like compound and glucose are likely to be produced by extracellular enzymes from the carbon source cellobiose. Indeed, cellotriose production from cellulose has been described for an extracellular extract of *Streptomyces* by Elwyn T. Reese [63]. Furthermore, it has been proposed that cellotriose and cellotetraose act as cellulase inducers in *Phanerochaete chrysosporium* [64] and that production of transglycosylation products from cellobiose is important for cellulase induction of *T. reesei* [9]. The formation of the cellotriose-like compound we detect, could be a side activity of CAZymes expressed at low basal level even in non-inducing conditions [65], which after detection by *T. reesei* enables full induction of the cellulytic system as has been previously suggested (for review see [8]). The fact that most of this cellotriose-like compound is produced by the cellulase deletion strain **Cel4d** (Fig. 2) suggests that it is made by an auxiliary enzyme, whose production is increased by cellulase deletion. Transport system for such small molecule inducers have been described [66, 67] and accordingly 5 out of our 18 top extracellular protein production rate correlated genes in continuous and batch conditions are transporters (Table 5). In addition the putative lactose and/or cellobiose permease *crt1* (3405) [13, 67] lies beside the homologue of *Neurospora crassaclr2* (26163) [15, 23] found in cluster 24 and having a FIRE predicted *Xyr1* element on its promoter region. The rlog2 expression values of *crt1* and *clr2* are correlated with a Pearson correlation of 0.94 in our experiment, hence they are likely to be commonly regulated also in general. Co-expression of clr2 and crt1 under cellulase inducing conditions have also been reported previously [15].

Glucose, like the cellotriose-like compound, can be expected to be a product of extracellular enzymes hydrolysing the carbon source cellobiose or the cellotriose-like product. However, why would it accumulate in conditions where carbon source starts to be consumed and there is ample living biomass? The answer could lie in the discrepancy between cellobiose consumption rate and growth rate (Figs. 1e, g, 3b). Although these two variables correlate strongly, the dynamic range of the growth rate is smaller and in particular the cellobiose uptake rate decelerates faster than the growth rate at 40–64 h. Hence, while the cells decelerate cellobiose uptake, the already secreted cellulotic system is not regulated and therefore residual glucose is temporarily accumulated.

Glycerol and ethanol are likely to be products of intracellular metabolism secreted to the growth medium. Glycerol is produced by *Saccharomyces cerevisiae* under anaerobic conditions, i.e. while it is producing ethanol, to compensate for cellular reactions that produce NADH and therefore to balance its redox state [68]. In addition, glycerol is produced as a response to a number of stresses in *S. cerevisiae* such as osmotic and oxidative stress [69, 70].

In our study, glycerol production was detected in all the cultures at the time when the biomass amount in the cultures was the highest (i.e. at 60  h), whereas ethanol was produced only in the cultures of the strain producing cutinase (**CutCBHd**) and its control strain (**CutCBHdCt**) at a time period preceding the glycerol production phase (i.e. from 16–24  h). It is possible that there would be transient oxygen limitation during the cultivation phase when biomass reaches the maximum level and starts to decline, i.e. at the same time when glycerol production was detected. However, due to the differences in the production time points, a direct link between glycerol and ethanol production in *T. reesei* is less plausible. At the time when glycerol production is detected, at 40–64  h, respectively, most protein production has already taken place. Possible stress conditions at that stage could be due to accumulation of vesicles and oxidative stress from protein folding [28, 71, 72].

The gene expression clusters whose expression correlates well with the rate of glycerol production are enriched in genes that encode proteins acting in the later steps of protein secretion after ER. These genes tend to be expressed at a higher level in the strains producing high amount of protein at 64  h as compared to the strains producing less protein (Fig. 5b). Hence, although there is no direct link between protein production and glycerol production, the 64h induction of these genes could be a response to accumulated protein secretion stress.

Although the ethanol production we detect is low and transient, ethanol production has been described for *T. reesei* also previously [73, 74]. We provide the first transcriptomics data set for such conditions which could be useful for development of a consolidated bioprocess of ethanol production.

We measured the concentrations of intracellular amino acids for time points 24, 40 and 64  h and found that the case-control strain pairs resemble each other, indicating that the strain history and other properties of the strains are more important explanatory factors for the intracellular amino acid content than the protein production load. Given these circumstances we did not try to use the intracellular amino acids for modelling, however most of the amino acids, especially vacuolar storage amino acids arginine and glutamine [75, 76], exhibit a clear downward trend over the cultivation time (Additional file 1: Figure S3). This apparent use of storage amino acids could explain why our flux balance analysis in many cases predicts lower protein production rates than are measured, in particular for early the time points at 16 and 24  h (Fig. 7). The cells could contain resources stored in the early phase of the cultivation which are not covered by our modelling, but which are in reality used in protein production.

It has been shown that protein production rates can be predicted for *Komagataella phaffii (Pichia pastoris)* based on online measurements of OUR and CER by a stoichiometric model [77]. Also in our data set such a relationship exists (Fig. 3f), but it breaks down in the samples where the protein production rate is the highest. In these sampling points the production rates are approximately 1000 times higher than in [77]. In general, our protein production rates are 10 to 100 times higher than those used in previous stoichiometric modelling of fungal protein production systems [32, 77–79]. Hence, the combination of *T. reesei* metabolic model and our cultivation data set provides a set up of modelling protein production in far more relevant conditions for industrial protein production. This could also indicate that at protein production rates typical for *T. reesei* yet undiscovered metabolic interactions exist.

It has been argued that the metabolic activity of cells, i.e. the flux through metabolic reactions, is controlled by the rate of carbon source uptake or by the rate of following key reactions [80–82]. As flux and transcript levels do not correlate in general in eukaryotes [83, 84] at least in the central carbon metabolism, what then controls transcript levels? In our experiment, based on the correlation of gene expression levels and cultivation variables (Table 2), the concentration of biomass and carbon source are the most important determinants of the transcriptome, effecting a similar set of genes. Although it has been shown that cell density has an effect on the trancriptome of *T. reesei*, the carbon source availability is a far more fundamental question for an organism and hence it is likely that the carbon source concentration is the main determinant of transcriptome. The fact that the carbon source uptake rate and carbon source concentration both exert control on different cellular levels highlights the importance of assessing both factors in order to understand the cell.

At the transcriptome level we find **two major responses in the secretion machinery** and **three major responses in amino acid metabolism**. Regarding the amino acid metabolism, the most prominent response is the general downward regulation of primary metabolism genes with declining carbon source concentration (mainly gene expression cluster 31, Fig. 4). Secondly, there is the induction of starvation related amino acid metabolism genes that have a negative correlation with the carbon source concentration (cluster 1). Thirdly, a special sub set of a amino acid metabolism genes is correlated with the protein production rate and differentially expressed between low and high producing strains (cluster 24, peak at 16  h and cluster 28 peak at 24  h) and putatively regulated by *Xyr1*. However, it is not obvious from our data why these individual genes are regulated.

The development of a stoichiometric genome wide metabolic model is an incremental project as exemplified by the progress from first published *S. cerevisiae* model [85] to the latest *S. cerevisiae* consensus model Yeast 7 [86]. Our *T. reesei* model was constructed without manual refinement though the CoReCo pipeline [55]. We have shown that the performance of models created using CoReCo is comparable to early *S. cerevisiae* models [55] in simulations. Our ability to predict protein production rates in this experiment with the *T. reesei* model shows its functionality and usefulness (Fig. 7). Overall the model predicted protein production rates of almost half the speed than were measured. Cellulases are heavily glycosylated proteins, a fact which our current model does not take this into account, but rather tries to produce all protein mass from amino acids. Amino acids are energetically very costly to produce for the cellular metabolism. Hence, the actual use of stored resources by the cells and omission of glycosylation could explain the prediction of lower than measured protein production rates.

The model predicts particularly high protein production rates at 40  h in comparison to the measured ones. As the transcriptome is mainly determined by the carbon source concentration (Table 3), which at 40  h has dropped 20–50  % from 16  h (Fig. 1c), this discrepancy is likely a result of transcriptional repression of main cellulases transcripts and/or the actual protein secretion machinery (Figs. 4, 6 clusters 24 and 28), yet again another factor not taken into account by our stoichiometric model.

The metabolism of cysteine and methionine (a precursor of cysteine) is not only highlighted by transcriptomics data, but also by our flux modelling (Fig. 9). In contrast to most metabolic reactions that correlate with the carbon source uptake rate as expected, the flux through the methionine salvage cycle exhibits a higher correlation with the protein production rate. In parallel, the gene expression for a cysteine synthase 76018 correlates with the protein production rate, but the flux through this reaction does not. Hence, it could be that cellulase protein production requires elevated cysteine and consequently methionine metabolisms and possibly flux to cysteine. Overall asparagine and cysteine are the two most over represented amino acids (43 and 37  % more, respectively) when comparing the relative amino acid contents of sequences of *T. reesei* proteins found to be secreted based on 2D-gel analysis [6] and other *T. reesei* proteins. More fundamentally, the capability to assimilate sulphur could be the limiting factor. A regulatory link between sulphur metabolism and cellulase expression has been shown earlier [87]. The induction of taurine catabolism genes at 24 and 40  h in high producing strains (**LipPr4d**, **LipPr4dCt** and **Cel4dCt**) also highlights the relevance of sulphur metabolism. In *T. reesei*’s natural environment taurine is a major source of sulphur [88]. Hence, the induction of these genes could be a natural, yet in industrial conditions futile, response to lack of sulphur.

The protein secretion machinery responds in our experiment by ER related genes that are correlated to protein production rate and are putatively controlled by *Xyr1* (Fig. 4 cluster 24) and by genes that are involved in the later stages of the secretory pathway, after ER and which are correlated to the glycerol production rate (Fig. 4, cluster 22). Such a regulation of the secretion machinery and amino acid metabolism by *Xyr1* has not been previously implicated based on expression data, although genome sequence analysis has proposed that *Xyr1* would regulate these and numerous other functional categories [50]. As no *hac1* splicing was detected, it seems that in these conditions mere *Xyr1* controlled induction of the secretion machinery is sufficient to cope with the protein production load.

We found that the expression of main cellulases followed the protein production rate (Fig. 6) and that their lack was compensated by expression of other cellulases. The induction of other cellulases implies that the repression under secretion stress (RESS) response was alleviated in the **Cel4d** strain [26].

In order to assess our results in the light of previous experiments we compared our analysis to an analysis of a large set of *T. reesei* microarray data [51] and found that genes involved in the major responses discussed above (gene expression clusters 22, 28 and 31, Fig. 4) are also co-regulated in the microarray data set. Furthermore, gene expression cluster 24 contains four genes (*clr2* 26163, *ace3* 77513, 108381, 70351) previously implicated as regulators of cellulase gene expression [15]. The overexpression of *ace3* 77513 and 108381 has been shown to have an effect on cellulase gene expression [15, 89, 90]. We have previously shown that although the level of paralogy in *T. reesei* genome is very low in general [91, 92], the genes of central carbon metabolism enzymes nevertheless have paralogous gene pairs with opposite regulation in response to protein production. Out of seven pairs of paralogues reported in [6] we detected five pairs with opposite regulation in response to protein production (LPD1a 67699, LPD1b 77373; LSC1a 22910, LSC1b 2223; LSC2a 103451, LSC2b 80881; PDB1a 76744, PDB1b 122745; TKL1-2a 120635, TKL1-2b 2211; TPI1a 68606, TPI1b 121789;) in this work. We also compared the correlation of gene expression to protein production rate between this experiment and [6] and found no similarity between the two studies. This is not surprising given the completely different physiological conditions in a chemostat [6] and a batch cultivation experiment. In this experiment we find that the carbon source concentration is the main determinant of transcriptome. However, in a chemostat the carbon source concentration is zero as all available carbon is immediately taken up. Therefore, in these batch experiments the growth rate appears as only a very minor determinant of the transcriptome while in a chemostat it emerges as dominant [6]. Nevertheless, central carbon metabolism paralogues and known key secretion pathway factors like *pdi1* (gene expression correlation to protein production rate is here 0.69 and 0.80 in [6] ), dpm2 (0.56 and 0.77, respectively) emerge from both experiments. Hence, genes correlating with the protein production rate in these completely different cultivation regimes could reveal novel key factors (Table 5).

## Conclusions

It has been argued that the flux of carbon source uptake can actively control metabolic activities of the cell. In our experiment the carbon source concentration appears as the main controller of the transcriptome. These parallel control systems would allow the cell to integrate information at different time scales: i.e. short-term responses (such as changes in metabolism in less than seconds) and long-term responses (such as transcriptional changes over hours). Their parallel existence also resolves the surprising lack of correlation between flux and transcriptome in eukaryotes.

Various approaches have been taken to improve protein production by filamentous fungi and *T. reesei* specifically. Manipulation of transcription factors has proven to be an efficient method to increase cellulase protein expression in many cases [15, 19]. Our modelling proposes that stoichiometry would allow production of more cellulases than measured, but the transcriptome becomes repressed due to depletion of the carbon source. Thus, the adjustment of metabolic control and the transcriptional regulation offers an interesting target for modification when aiming at improved protein production, e.g. by alleviating such a repression.

As a completely novel target for improvement of cellulase production our data analysis and modelling proposes processes of sulphur assimilation and cysteine metabolism. As amino acid metabolism is essential and tightly controlled at many levels detailed modelling will be required to select the correct targets and their manipulations.

## Methods

### Strains

The strains used in this study were all derivatives *T. reesei* VTT D-00775.The major cellulase genes were deleted by successive rounds of genetic modification of *T. reesei* VTT D-00775 $\Delta$*mus53* resulting in the strain *T. reesei* VTT D-00775 $\Delta$*mus53*$\Delta$*cbh1*$\Delta$*cbh2*$\Delta$*egl1*$\Delta$*egl2*. The cellulase deletion strain was assigned in this study as **Cel4d** and corresponding control strain as **Cel4dCt**.

*Coprinus cinerea* cutinase (CC1G_09668.1) was expressed from *cbh1* locus under *cbh1* promoter and terminator using the *T. reesei* VTT D-00775 $\Delta$*cbh1* as a host [93]. The cutinase producing strain was assigned as **CutCBHd** in this study and the control strain, *T. reesei* VTT D-00775 in which *cbh1* was replaced by an acetamidase marker gene, as **CutCBHdCt**.

*Dipodascus capitatus* lipase was expressed under *cbh1* promoter and terminator in *T. reesei* strain VTT D-00775 $\Delta$*mus53* deleted for four protease genes. The codon usage in the cDNA encoding the lipase was adapted to the codon bias of *T. reesei* genes and the native signal sequence (24 aa) was replaced with *cbh1* signal sequence of *T. reesei* and a N-terminal Strep tag was added in the expression construct. The lipase producing strain is assigned as **LipPr4d** in this study and the corresponding control strain as **LipPr4dCt**.

### Cultivation procedures

Fungal precultures for bioreactors were carried out as follows. 8 × 10$^7$ fungal spores were transferred to 400 ml of culture medium (20 g/l cellobiose, 7.6 g/l (NH$_{4}$)$_{2}$SO$_{4}$, 15.0 g/l KH$_{2}$PO$_{4}$, 2.4 mM MgSO$_{4}\cdot$7H$_{2}$O, 4.1 mM CaCl$_{2}\cdot$H$_{2}$O, 3.7 mg/l CoCl$_{2}$, 5 mg/l FeSO$_{4}\cdot$7H$_{2}$O, 1.4 mg/l ZnSO$_{4}\cdot$7H$_{2}$O, 1.6 mg/l MnSO$_{4}\cdot$7H$_{2}$O, pH adjusted to 5.2 with KOH) and cultivated in shake flasks on rotary shaker (250 rpm) at $28\,^{\circ }\mathrm {C}$ for 3 days. Sartorius Q plus bioreactors containing 900 ml of the medium (25 g/l cellobiose, 4.4 g/l (NH$_{4}$)$_{2}$SO$_{4}$, 15.0 g/l KH$_{2}$PO$_{4}$, 2.64 mM MgSO$_{4}\cdot$7H$_{2}$O, 4.5 mM CaCl$_{2}\cdot$H$_{2}$O, 4.1 mg/l CoCl$_{2}$, 5.5 mg/l FeSO$_{4}\cdot$7H$_{2}$O, 1.54 mg/l ZnSO$_{4}\cdot$7H$_{2}$O, 1.76 mg/l MnSO$_{4}\cdot$7H$_{2}$O) were inoculated with 100 ml of the preculture. Cultivation temperature was $28\,^{\circ }\mathrm {C}$. The pH was adjusted 4.8 ±0.1 by addition of KOH or H$_{3}$PO$_{4}$. The dissolved oxygen saturation level in the cultures was >30  %, agitation 500–1200 rpm with the tip speed of 1.1–2.7 m/s and total aeration flow 0.6 l/min. Samples of the cultures were withdrawn at 0, 16, 24, 40, 64, 88 and 112 h after inoculation of the bioreactors. The mycelial samples were separated from the culture supernatant by filtering through Whatman 3MM, frozen immediately in liquid nitrogen and stored at $-80\,^{\circ }\mathrm {C}$ for further analysis. Culture supernatant samples were stored at $-20\,^{\circ }\mathrm {C}$. For sugar analytics, 1.5 ml culture supernatant samples were acidified by the addition of 10 ${\upmu}$l of 97  % H$_{2}$SO$_{4}$ before storing.

Each strain was cultivated in triplicate and subsequent sampling and analyses of all the times points was also carried out from each of the three repeats.

### Sample preparation and analytics of the bioreactor cultures

Biomass dry weight in the cultures was measured by filtering and drying the mycelium samples at $105\,^{\circ }\mathrm {C}$ to a constant weight (24 h). Sugars, sugar acids and alcohols in the culture supernatant and medium were analysed using HPLC. Glycerol and cellotriose-like compounds were further confirmed with GC-MS and LC-MS, respectively, see Additional file 1: Figures S11, S12. Soluble protein secreted into the culture medium was measured using Bio-Rad Protein Assay kit. Enzyme activity against the substrate 4-methylumbelliferryl-β-d-lactoside (MUL) was measured as described [94].

Mycelial samples collected at the time points of 16, 24, 40 and 64  h from the cultures were subjected to transcriptome analysis. Frozen mycelium was ground under liquid nitrogen and total RNA was isolated with Trizol reagent according to the manufacturer’s instructions. RNA was subsequently purified using RNeasy Mini Kit (Qiagen, Hilden, Germany) and RNA concentration was measured using NanoDrop ND-1000 (NanoDrop Technologies Inc. Wilmington, DE, USA). Integrity of the isolated RNA was verified using an Agilent 2100 Bioanalyzer (Agilent Technologies, Palo Alto, CA, USA).

For determination of free amino acids in the fungal cells, mycelium samples ground under liquid nitrogen were resuspended in water. 500 μl aliquots (containing 10–15 mg dry weight/ml) were sonicated using a MSE 150 W sonicator (18 μm amplitude, eight cycles of 8 s sonication and 30 s cooling on ice between the sonication cycles). The samples were first diluted 1/10 and 1/100. Dissolved proteins were precipitated with sulfosalicylic acid (final concentration of 5  %, w/v) and the samples were centrifuged. 10 μl of the supernatant was mixed with 10 μl of internal standard solution (25 μM norvaline, Sigma-Aldrich, St. Luis, Missouri, USA) and 60 μl of AccQ·Tag Borate buffer (Waters, Milford, MA, USA). The mixture was vortexed for 30 s, 20 μl AccQ·Tag reagent (Waters, Milford, MA, USA) was added and sample mixture was instantly vortexed before incubation at $55\,^{\circ }\mathrm {C}$ for 10 min. Amino acid standards were derivatized as the samples. Amino Acid Standard Solution, Amino Acid Standards Physiological, Basics, l-isoleusine, glutamine and norvaline were all obtained from Sigma-Aldrich (St. Luis, Missouri, USA). Amino acid analysis was performed on an Acquity UPLC system, Waters (Milford, MA, USA) with diode array detector. Chromatography was performed using an Acquity Mass TRAKtm (2.1 × 100 mm, 1.7 μm) column, Waters (Milford, USA), kept at $43\,^{\circ }\mathrm {C}$. Injection volume was 1 μl. Separation was performed using gradient elution with 10  % (v/v) Amino Acid Analysis concentrate A in water (A) and Amino Acid Analysis eluent B (B) at a flow rate of 0.4 ml/min using a gradient elution program. Signal was detected at 260 nm (2.4 nm resolution, 20 points/s). Standards were derivatized as the samples. Ala, Arg, Asn, Asp, Cys, Glu, Gln, Gly, His, Ile, Leu, Lys, Met, Phe, Pro, Ser, Thr, Trp, Tyr, Val as well as several related compounds were quantified in the samples.

For the determination of total amino acid content of extracellular proteins, 100 μl samples of culture supernatant were pipetted to hydrolysis tubes (PierceTM Thermo ScientificTM) together with the internal standard solution (norvaline, Sigma-Aldrich (St. Luis, Missouri, USA)) and freeze dried. The samples were hydrolysed with 100 ${\upmu}$l of 6 N HCl for 24 h after which the hydrolysed samples were evaporated to dryness and reconstituted in 100 ${\upmu}$l of H2O. All the samples were further diluted 1/10 and 1/50. Derivatization was done with AccQ·Fluor reagent kit (Waters (Milford, MA, USA)). AccQ·Fluor reagent was reconstituted in acetonitrile (350 ${\upmu}$l), vortexed for 10 s, heated at $55\,^{\circ }\mathrm {C}$ and vortexed until dissolved. AccQ·Fluor Borate buffer (60 ${\upmu}$L) and H20 (10 ${\upmu}$l) were added to10 ${\upmu}$l of sample solution (non-diluted and dilutions 1/10 and 1/50) and finally, the AccQ · Fluor reagent (20 ${\upmu}$L) was added and the sample mixture was instantly vortexed for 60 seconds. Samples were preserved at $10\,^{\circ }\mathrm {C}$ before analysis. Amino Acid Standard Solution, Amino Acid Standards Physiological Basics, l-isoleusine and glutamine were obtained from (Sigma-Aldrich (St. Luis, Missouri, USA)). The standards were derivatized as the samples. UPLC analysis of the amino acids was performed on an Acquity UPLC system, Waters (Milford, MA, USA) with diode array detector. Chromatography was performed using an Acquity Mass TRAK tm (2.1 × 100 mm, 1.7 ${\upmu}$m) column, Waters (Milford, USA), kept at $43\,^{\circ }\mathrm {C}$. Injection volume was 1 ${\upmu}$L. Separation was performed using gradient elution with 10 % (v/v) Amino Acid Analysis concentrate A in water (A) and Amino Acid Analysis eluent B (B) at a flow rate of 0.4 ml/min using a gradient elution program. Mass TRAKTM Amino Acid Analysis concentrate A and eluent B were obtained from Waters (Milford, MA, USA). Signal was detected at 260 nm (2.4 nm resolution, 20 points/second). Standards were derivatized as the samples. His, Ser, Arg Gly, Asp, Glu, Thr, Ala, Pro, Lys, Tyr, Met, Val, Ile, Leu and Phe were quantified in the samples. In acid hydrolysis, Asn is converted to Asp and Gln to Glu. Therefore, quantifications of Asn and Gln as well as acid labile Trp and Cys and Met that was oxidised, were not obtained by the method.

### Cultivation data analysis

From the concentration measurements of various compounds their rates were modeled as heteroscedastic Gaussian processes [38] using the ’nsgp’ R package. The approach results in probabilistic interpolation models for the concentration and allows deriving the first order derivatives, i.e. the uptake and secretion rates, in analytical form resulting in accurate derivative estimates [62]. The distribution means were extracted as the most likely concentration and rate curves over time. The fit of the models to the data is shown in Additional file 1: Figure S13.

### RNA sequencing and data analysis

RNA library preparation and sequencing was carried out by Source BioSciences (Nottingham, UK). In brief, TruSeq stranded pair-end library was prepared for each of the 72 samples and sequenced with Illumina HiSeq 2000 for 100 bp for both pair ends. The data has been submitted to NCBI SRA as BioProject PRJNA293671. Read data was trimmed with SKEWER [95] and quality controlled with FastQC (http://www.bioinformatics.babraham.ac.uk/projects/fastqc/). Reads were aligned with to *T. reesei* genome version 2.0 [91] retrieved with GFF annotations from EnsemblFungi [96] and reads counted with R package GenomicFeatures [97]. Quality of repeats was assessed with sample wise Principal Component Analysis (PCA) of FPKMs. Differential expression analysis of read counts was carried out with DESeq2 [40] and differentially expressed genes clustered with Bayesin hierarchical clustering [41]. Gene annotations were retrieved from [6] and *T. reesei* genome version 2.0 site (http://www.genome.jgi.doe.gov/Trire2/Trire2.home.html). Gene set enrichments were calculated with GOstats [98] for GO annotations using a custom built *T. reesei* genome annotation AnnotationDBI package [99] and with hypergeometric test for other annotations. Correlations between cultivation parameters and gene expression were calculated as Spearman rank-sum correlations and the false discovery rate was estimated from the Q-value [100] using the R package ’qvalue’. All gene list comparisons were done with R package GeneOverlap [101].

Gene names in capitals are derived from the *S. cerevisiae* according to Saccharomyces Genome Database [102] while names in italics are from other fungal species as specified. Numbers after gene names or descriptions refer to *T. reesei* genome version 2.0 gene identifiers.

### Flux balance analysis

**Table 6:** Enrichment of metabolic pathway is flux clusters

Pathway ID	Pathway name	Cluster	Count of ECs	*p* value
LYSINE-AMINOAD-PWY	Lysine biosynthesis IV	1	4	0.0000582
PWY-3081	Lysine biosynthesis V	1	4	0.0000582
ARO-PWY	Chorismate biosynthesis I	2	6	0.0004460
HISTSYN-PWY	Histidine biosynthesis	2	7	0.0007221
PWY-6163	Chorismate biosynthesis from 3-dehydroquinate	2	4	0.0062252
PWY-6123	Inosine-5-phosphate biosynthesis I IMP biosynthesis I PWY-6124 inosine-5-phosphate biosynthesis II	2	3	0.0226875
PWY-6124	Inosine-5’-phosphate biosynthesis II	2	3	0.0226875
TRPSYN-PWY	Tryptophan biosynthesis	2	3	0.0226875
rn00400	Phenylalanine, tyrosine and tryptophan biosynthesis	2	11	0.0428314
PWY-6519	7-keto-8-aminopelargonate biosynthesis I	5	7	0.00000008
PWY-6282	Palmitoleate biosynthesis I	5	4	0.0005743
FASYN-ELONG-PWY	Fatty acid elongation-saturated	5	3	0.0012710
PWY0-862	(5Z)-dodec-5-enoate biosynthesis	5	3	0.0012710
PWYG-321	Mycolate biosynthesis	5	3	0.0012710
PWY-5989	Stearate biosynthesis II (bacteria and plants)	5	3	0.0047122
PWY-5971	Palmitate biosynthesis II (bacteria and plants)	5	3	0.0109189
PWY-5994	Palmitate biosynthesis	5	3	0.0109189
GO:0004312	Fatty acid synthase activity	5	3	0.0328365
HOMOSERSYN-PWY	Homoserine biosynthesis	8	3	0.0093657
SUCSYN-PWY	Sucrose biosynthesis I	8	3	0.0318091
PWY-5901	2,3-dihydroxybenzoate biosynthesis	9	3	0.0000052
rn01053	Biosynthesis of siderophore group nonribosomal peptides	9	3	0.0000052
rn00364	Fluorobenzoate degradation	12	3	0.0007013
rn00361	Chlorocyclohexane and chlorobenzene degradation	12	3	0.0034159
rn00627	Aminobenzoate degradation	12	3	0.0034159
rn00270	Cysteine and methionine metabolism	13	7	0.004274
ARGSYNBSUB-PWY	Arginine biosynthesis II (acetyl cycle)	14	4	0.0000001
GLUTORN-PWY	Ornithine biosynthesis	14	3	0.0000052
PWY-5154	Arginine biosynthesis III	14	3	0.0000208
rn00330	Arginine and proline metabolism	14	4	0.0190572

All stoichiometric modeling was carried out with the R package Sybil [103]. A single FBA model was constructed for each time point of each strain. FBA was constrained with the modeled rates of carbon source uptake and growth. The carbon sources cellotriose, cellobiose and glucose were combined into one glucose uptake reaction and glycerol modeled as taken up separately. The lower and upper bound for each of these three constraints (growth rate, glucose uptake and glycerol uptake) was relaxed by ±5  % from the actual modeled rate to allow sufficient space for the solver to find solutions. Extracellular protein production was modeled as a single reaction as in [104] and used as objective. For determining the equation describing protein production from its amino acid precursors, the measured amount of total protein and the ratio of the measured amino acids were used. For the amino acids without measurement data the ratio was estimated based on the codon frequency in transcripts encoding secreted proteins in RNA sequencing data (Additional file 2: Table S6). To transform the extracellular protein rate of (g/gCDW h) to mmol/(gCDW h) a molecular weight of 56545 Da was used. This is the average of protein sequence based molecular weights of proteins found to be secreted based on 2D-gel analysis [6]. All flux distributions from FBA were retrieved with “minimization of absolute total flux”-step in Sybil [103] to get realistic flux distributions. The flux distributions were subsequently filtered with FVA (flux variability analysis).

In order to filter out fluxes that could not be reliably determined with the given constraints, only those reactions whose flux did not vary more than five flux units in any condition in FVA were kept. Combined fluxes from all models were clustered with Bayesin hierarchical clustering [41] and reactions of clusters mapped by EC to pre-defined pathways in KEGG [105] and Metacyc [106] databases. Enrichment of EC cluster members on predefined pathways was quantified with the hypergeometric test.

## Additional files

10.1186/s13068-016-0547-5 Supplementary Figures. A PDF file containing all the supplementary figures referred to in the text.
10.1186/s13068-016-0547-5 Supplementary Tables. An XLS file containing all the supplementary tables referred to in the text.

## Abbreviations

CWD: cell dry weight
FBA: flux balance analysis
CER: specific CO_2_ exchange rate
OUR: specific O_2_ consumption rate
FPKM: fragments per kilobase of exon per million fragments
FDR: false discovery rate
FVA: flux variability analysis

## References

[1] Cherry J, Fidantsef A (2003). Directed evolution of industrial enzymes: an update. Curr Opin Biotechnol.

[2] Pakula T, Salonen K, Uusitalo J, Penttilä M (2005). The effect of specific growth rate on protein synthesis and secretion in the filamentous fungus Trichoderma reesei. Microbiology.

[3] Castillo FJ, Blanch HW, Wilke CR (1984). Lactase production in continuous culture by Trichoderma reesei Rut-C30. Biotechnol Lett.

[4] Schafner DW, Toledo RT (1992). Cellulase production in continuous culture by Trichoderma reesei on xylose-based media. Biotechnol Bioeng.

[5] Chaudhuri BK, Sahai V (1994). Comparison of growth and maintenance parameters for cellulase biosynthesis by Trichoderma reesei-C5 with some published data. Enzym Microb Technol.

[6] Arvas M, Pakula T, Smit B, Rautio J, Koivistoinen H, Jouhten P, Lindfors E, Wiebe M, Penttilä M, Saloheimo M (2011). Correlation of gene expression and protein production rate—a system wide study. BMC Genomics.

[7] Aro N, Pakula T, Penttilä M (2005). Transcriptional regulation of plant cell wall degradation by filamentous fungi. FEMS Microbiol Rev.

[8] Amore A, Giacobbe S, Faraco V (2013). Regulation of cellulase and hemicellulase gene expression in fungi. Curr Genomics.

[9] Shida Y, Yamaguchi K, Nitta M, Nakamura A, Takahashi M, Kidokoro SI, Mori K, Tashiro K, Kuhara S, Matsuzawa T, Yaoi K, Sakamoto Y, Tanaka N, Morikawa Y, Ogasawara W (2015). The impact of a single-nucleotide mutation of bgl2 on cellulase induction in a Trichoderma reesei mutant. Biotechnol Biofuels.

[10] Ilmen M, Saloheimo A, Onnela M-L, Penttilä ME (1997). Regulation of cellulase gene expression in the filamentous fungus Trichoderma reesei. Appl Environ Microbiol.

[11] Iyayi CB, Bruchmann E-E, Kubicek CP (1989). Induction of cellulase formation in Trichoderma reesei by cellobiono-1,5-lacton. Arch Microbiol.

[12] Häkkinen M, Arvas M, Oja M, Aro N, Penttilä M, Saloheimo M, Pakula TM (2012). Re-annotation of the CAZy genes of Trichoderma reesei and transcription in the presence of lignocellulosic substrates. Microb Cell Fact.

[13] Ivanova C, Bååth JA, Seiboth B, Kubicek CP (2013). Systems analysis of lactose metabolism in Trichoderma reesei identifies a lactose permease that is essential for cellulase induction. PLoS One.

[14] Ries L, Pullan ST, Delmas S, Malla S, Blythe MJ, Archer DB (2013). Genome-wide transcriptional response of Trichoderma reesei to lignocellulose using RNA sequencing and comparison with Aspergillus niger. BMC Genomics.

[15] Häkkinen M, Valkonen MJ, Westerholm-Parvinen A, Aro N, Arvas M, Vitikainen M, Penttilä M, Saloheimo M, Pakula TM (2014). Screening of candidate regulators for cellulase and hemicellulase production in Trichoderma reesei and identification of a factor essential for cellulase production. Biotechnol Biofuels.

[16] Poggi-Parodi D, Bidard F, Pirayre A, Portnoy T, Blugeon C, Seiboth B, Kubicek CP, Crom SL, Margeot A (2014). Kinetic transcriptome analysis reveals an essentially intact induction system in a cellulase hyper-producer Trichoderma reesei strain. Biotechnol Biofuels.

[17] dos Santos Castro L, Pedersoli W, Antoniê AC, Steindorff A, Silva-Rocha R, Martinez-Rossi NM, Rossi A, Brown N, Goldman GH, Faç VM, Persinoti GF, Silva R (2014). Comparative metabolism of cellulose sophorose and glucose in Trichoderma reesei using high-throughput genomic and proteomic analyses. Biotechnol Biofuels.

[18] Häkkinen M, Sivasiddarthan D, Aro N, Saloheimo M, Pakula TM (2015). The effects of extracellular pH and of the transcriptional regulator PACI on the transcriptome of Trichoderma reesei. Microb Cell Fact.

[19] Ilmé M, Thrane C, Penttilä M (1996). The glucose repressor genecre1 of Trichoderma: isolation and expression of a full-length and a truncated mutant form. Mol Gen Genet.

[20] Rauscher R, Wurleitner E, Wacenovsky C, Aro N, Stricker AR, Zeilinger S, Kubicek CP, Penttilä M, Mach RL (2006). Transcriptional regulation of xyn1 encoding xylanase I, in Hypocrea jecorina. Eukaryot Cell.

[21] Saloheimo A (2000). Isolation of the ace1 Gene Encoding a Cys2-His2 Transcription Factor Involved in Regulation of Activity of the Cellulase Promoter cbh1 of Trichoderma reesei. J Biol Chem.

[22] Aro N, Saloheimo A, Ilmen M, Penttila M (2001). ACEII a novel transcriptional activator involved in regulation of cellulase and xylanase genes of Trichoderma reesei. J Biol Chem.

[23] Coradetti ST, Craig JP, Xiong Y, Shock T, Tian C, Glass NL (2012). Conserved and essential transcription factors for cellulase gene expression in ascomycete fungi. Proc Natl Acad Sci USA.

[24] Seiboth B, Karimi R, Phatale P, Linke R, Hartl L, Sauer D, Smith K, Baker S, Freitag M, Kubicek C (2012). The putative protein methyltransferase LAE1 controls cellulase gene expression in Trichoderma reesei. Mol Microbiol.

[25] Saloheimo M, Valkonen M, Penttilä M (2003). Activation mechanisms of the HACI-mediated unfolded protein response in filamentous fungi. Mol Microbiol.

[26] Pakula TM, Laxell M, Huuskonen A, Uusitalo J, Saloheimo M, Penttila M (2003). The effects of drugs inhibiting protein secretion in the filamentous fungus Trichoderma reesei: evidence for down-regulation of genes that encode secreted proteins in the stressed cells. J Biol Chem.

[27] Saloheimo M, Lund M, Penttilä M (1999). The protein disulphide isomerase gene of the fungus Trichoderma reesei is induced by endoplasmic reticulum stress and regulated by the carbon source. Mol Gen Genet.

[28] Arvas M, Pakula T, Lanthaler K, Saloheimo M, Valkonen M, Suortti T, Robson G, Penttilä M (2006). Common features and interesting differences in transcriptional responses to secretion stress in the fungi Trichoderma reesei and Saccharomyces cerevisiae. BMC Genomics.

[29] Saloheimo M, Pakula TM (2011). The cargo and the transport system: secreted proteins and protein secretion in Trichoderma reesei (Hypocrea jecorina). Microbiology.

[30] Simeonidis E, Price ND (2015). Genome-scale modeling for metabolic engineering. J Ind Microbiol Biotechnol.

[31] Klein T, Niklas J, Heinzle E (2015). Engineering the supply chain for protein production/secretion in yeasts and mammalian cells. J Ind Microbiol Biotechnol.

[32] Nocon J, Steiger MG, Pfeffer M, Sohn SB, Kim TY, Maurer M, Rußmayer H, Pflügl S, Ask M, Haberhauer-Troyer C, Ortmayr K, Hann S, Koellensperger G, Gasser B, Lee SY, Mattanovich D (2014). Model based engineering of Pichia pastoris central metabolism enhances recombinant protein production. Metab Eng.

[33] Gritzali M, Brown RD. The cellulase system of Trichoderma. In: advances in chemistry. Washington: American Chemical Society (ACS); 1979. pp. 237–260. http://www.dx.doi.org/10.1021/ba-1979-0181.ch012. Accessed 19 May 2016.

[34] Herpoël-Gimbert I, Margeot A, Dolla A, Jan G, Mollé D, Lignon S, Mathis H, Sigoillot J-C, Monot F, Asther M (2008). Comparative secretome analyses of two Trichoderma reesei RUT-C30 and CL847 hypersecretory strains. Biotechnol Biofuels.

[35] Adav SS, Chao LT, Sze SK (2013). Protein abundance in multiplexed samples (PAMUS) for quantitation of Trichoderma reesei secretome. J Proteomics.

[36] Nummi M, Niku-Paavola ML, Lappalainen A, Enari TM, Raunio V (1983). Cellobiohydrolase from Trichoderma reesei. Biochem J.

[37] Steiger MG, Vitikainen M, Uskonen P, Brunner K, Adam G, Pakula T, Penttila M, Saloheimo M, Mach RL, Mach-Aigner AR (2010). Transformation system for Hypocrea jecorina (Trichoderma reesei) that favors homologous integration and employs reusable bidirectionally selectable markers. Appl Environ Microbiol.

[38] Heinonen M, Guipaud O, Milliat F, Buard V, Micheau B, Tarlet G, Benderitter M, Zehraoui F, Alche-Buc F (2015). Detecting time periods of differential gene expression using Gaussian processes: an application to endothelial cells exposed to radiotherapy dose fraction. Bioinformatics.

[39] Leinonen R, Sugawara H, Shumway M (2010). The sequence read archive. Nucleic Acids Res.

[40] Love M, Huber W, Anders S (2014). Moderated estimation of fold change and dispersion for RNA-seq data with DESeq2. Genome Biol.

[41] Savage RS, Heller K, Xu Y, Ghahramani Z, Truman WM, Grant M, Denby KJ, Wild DL (2009). R/BHC: fast Bayesian hierarchical clustering for microarray data. BMC Bioinformatics.

[42] Elemento O, Slonim N, Tavazoie S (2007). A universal framework for regulatory element discovery across all genomes and data types. Mol Cell.

[43] Kumar L, Breakspear A, Kistler C, Ma L-J, Xie X (2010). Systematic discovery of regulatory motifs in Fusarium graminearum by comparing four Fusarium genomes. BMC Genomics.

[44] Gasch AP, Spellman PT, Kao CM, Carmel-Harel O, Eisen MB, Storz G, Botstein D, Brown PO (2000). Genomic expression programs in the response of yeast cells to environmental changes. Mol Biol Cell.

[45] Proft M (2001). Regulation of the Sko1 transcriptional repressor by the Hog1 MAP kinase in response to osmotic stress. EMBO J.

[46] Andersen MR, Vongsangnak W, Panagiotou G, Salazar MP, Lehmann L, Nielsen J (2008). A trispecies Aspergillus microarray: comparative transcriptomics of three Aspergillus species. Proc Natl Acad Sci.

[47] van Peij NNME, Visser J, de Graaff LH (1998). Isolation and analysis of xln R encoding a transcriptional activator co-ordinating xylanolytic expression in Aspergillus niger. Mol Microbiol.

[48] Marui J, Tanaka A, Mimura S, de Graaff LH, Visser J, Kitamoto N, Kato M, Kobayashi T, Tsukagoshi N (2002). A transcriptional activator AoXlnR controls the expression of genes encoding xylanolytic enzymes in Aspergillus oryzae. Fungal Genet Biol.

[49] Furukawa T, Shida Y, Kitagami N, Mori K, Kato M, Kobayashi T, Okada H, Ogasawara W, Morikawa Y (2009). Identification of specific binding sites for XYR1 a transcriptional activator of cellulolytic and xylanolytic genes in Trichoderma reesei. Fungal Genet Biol.

[50] dos Silva-Rocha R, Santos Castro L, Antoniê ACC, Guazzaroni ME, Persinoti GF, Silva RN (2014). Deciphering the Cis-regulatory elements for XYR1 and CRE1 regulators in Trichoderma reesei. PLoS One.

[51] Vinko O. Inferring Trichoderma reesei gene regulatory network. Bachelor Thesis. https://www.aaltodoc.aalto.fi/handle/123456789/11809. Accessed 19 May 2016.

[52] Segal E, Taskar B, Gasch A, Friedman N, Koller D (2001). Rich probabilistic models for gene expression. Bioinformatics.

[53] Varma A, Palsson BO (1994). Metabolic flux balancing: basic concepts scientific and practical use. Bio/Technology.

[54] Orth J, Thiele I, Palsson B (2010). What is flux balance analysis?. Nat Biotechnol.

[55] Pitkänen E, Jouhten P, Hou J, Syed MF, Blomberg P, Kludas J, Oja M, Holm L, Penttilä M, Rousu J, Arvas M (2014). Comparative genome-scale reconstruction of gapless metabolic networks for present and ancestral species. PLoS Comput Biol.

[56] Mahadevan R, Schilling C (2003). The effects of alternate optimal solutions in constraint-based genome-scale metabolic models. Metab Eng.

[57] Albers E (2009). Metabolic characteristics and importance of the universal methionine salvage pathway recycling methionine from 5’-methylthioadenosine. IUBMB Life.

[58] Collen A, Saloheimo M, Bailey M, Penttilä M, Pakula TM (2005). Protein production and induction of the unfolded protein response inTrichoderma reesei strain Rut-C30 and its transformant expressing endoglucanase I with a hydrophobic tag. Biotechnol Bioeng.

[59] Jourdier E, Poughon L, Larroche C, Monot F, Chaabane F (2012). A new stoichiometric miniaturization strategy for screening of industrial microbial strains: application to cellulase hyper-producing Trichoderma reesei strains. Microb Cell Fact.

[60] Friedman J, Hastie T, Tibshirani R (2009). The elements of statistical learning.

[61] Koch AL, Koch A, Robinson JA, Milliken GA (1998). The monod model and its alternatives. Mathematical modeling in microbial ecology.

[62] Solak E, Murray-Smith R, Leithead W, Leith D, Rasmussen C (2003). Derivative observations in Gaussian process models of dynamic systems. Appear Adv Neural Inf Process Syst.

[63] Reese ET, Smakula E, Perlin AS (1959). Enzymic production of cellotriose from cellulose. Arch Biochem Biophys.

[64] Suzuki H, Igarashi K, Samejima M (2010). Cellotriose and cellotetraose as inducers of the genes encoding cellobiohydrolases in the basidiomycete phanerochaete chrysosporium. Appl Environ Microbiol.

[65] Escobar-Vera J (1997). Cellulase induction in Trichoderma reesei by cellulose requires its own basal expression. J Biol Chem.

[66] Kubicek CP, Messner R, Gruber F, Mandels M, Kubicek-Pranz EM (1993). Triggering of cellulase biosynthesis by cellulose in Trichoderma reesei. Involvement of a constitutive, sophorose-inducible, glucose-inhibited beta-diglucoside permease. J Biol Chem.

[67] Zhang W, Kou Y, Xu J, Cao Y, Zhao G, Shao J, Wang H, Wang Z, Bao X, Chen G, Liu W (2013). Two major facilitator superfamily sugar transporters from Trichoderma reesei and their roles in induction of cellulase biosynthesis. J Biol Chem.

[68] Vandijken J, Scheffers W (1986). Redox balances in the metabolism of sugars by yeasts. FEMS Microbiol Lett.

[69] Hohmann S (2002). Osmotic stress signaling and osmoadaptation in yeasts. Microbiol Mol Biol Rev.

[70] Petelenz-Kurdziel E, Kuehn C, Nordlander B, Klein D, Hong K-K, Jacobson T, Dahl P, Schaber J, Nielsen J, Hohmann S, Klipp E (2013). Quantitative analysis of glycerol accumulation glycolysis and growth under hyper osmotic stress. PLoS Comput Biol.

[71] Harding HP, Zhang Y, Zeng H, Novoa I, Lu PD, Calfon M, Sadri N, Yun C, Popko B, Paules R, Stojdl DF, Bell JC, Hettmann T, Leiden JM, Ron D (2003). An integrated stress response regulates amino acid metabolism and resistance to oxidative stress. Mol Cell.

[72] Tyo KE, Liu Z, Petranovic D, Nielsen J (2012). Imbalance of heterologous protein folding and disulfide bond formation rates yields runaway oxidative stress. BMC Biol.

[73] Xu Q, Singh A, Himmel ME (2009). Perspectives and new directions for the production of bioethanol using consolidated bioprocessing of lignocellulose. Curr Opin Biotechnol.

[74] Huang J, Chen D, Wei Y, Wang Q, Li Z, Chen Y, Huang R (2014). Direct ethanol production from lignocellulosic sugars and sugarcane bagasse by a recombinant Trichoderma reesei strain HJ48. Sci World J.

[75] Sato T, Ohsumi Y, Anraku Y (1984). Substrate specificities of active transport systems for amino acids in vacuolar-membrane vesicles of Saccharomyces cerevisiae. Evidence of seven independent proton/amino acid antiport systems. J Biol Chem.

[76] Cramer C, Vaughn L, Davis R (1980). Basic amino acids and inorganic polyphosphates in Neurospora crassa: independent regulation of vacuolar pools. J Bacteriol.

[77] Tortajada M, Llaneras F, Ramó D, Picó J (2012). Estimation of recombinant protein production in Pichia pastoris based on a constraint-based model. J Process Control.

[78] Driouch H, Melzer G, Wittmann C (2012). Integration of in vivo and in silico metabolic fluxes for improvement of recombinant protein production. Metab Eng.

[79] Jordà J, Jouhten P, Cá E, Maaheimo H, Albiol J, Ferrer P (2012). Metabolic flux profiling of recombinant protein secreting Pichia pastoris growing on glucose:methanol mixtures. Microb Cell Fact.

[80] Huberts DHEW, Niebel B, Heinemann M (2011). A flux-sensing mechanism could regulate the switch between respiration and fermentation. FEMS Yeast Res.

[81] Gerosa L, Sauer U (2011). Regulation and control of metabolic fluxes in microbes. Curr Opin Biotechnol.

[82] Kochanowski K, Volkmer B, Gerosa L, van Rijsewijk BRH, Schmidt A, Heinemann M (2013). Functioning of a metabolic flux sensor in Escherichia coli. Proc Natl Acad Sci.

[83] Machado D, Herrgård M (2014). Systematic evaluation of methods for integration of transcriptomic data into constraint-based models of metabolism. PLoS Comput Biol.

[84] Schwender J, König C, Klapperstück M, Heinzel N, Munz E, Hebbelmann I, Hay JO, Denolf P, Bodt SD, Redestig H, Caestecker E, Jakob PM, Borisjuk L, Rolletschek H (2014). Transcript abundance on its own cannot be used to infer fluxes in central metabolism. Front Plant Sci.

[85] Forster J (2003). Genome-scale reconstruction of the Saccharomyces cerevisiae metabolic network. Genome Res.

[86] Aung HW, Henry SA, Walker LP (2013). Revising the representation of fatty acid glycerolipid and glycerophospholipid metabolism in the consensus model of yeast metabolism. Ind Biotechnol.

[87] Gremel G, Dorrer M, Schmoll M (2008). Sulphur metabolism and cellulase gene expression are connected processes in the filamentous fungus Hypocrea jecorina (anamorph Trichoderma reesei). BMC Microbiol.

[88] Schmoll M, Dattenböck C, Carreras-Villaseñor N, Mendoza-Mendoza A, Tisch D, Alemán M, Baker S, Brown C, Cervantes-Badillo M, Cetz-Chel J, Cristobal-Mondragon G, Delaye L, Esquivel-Naranjo E, Frischmann A, Gallardo-Negrete JJ, García-Esquivel M, Gomez-Rodriguez E, Greenwood D, Hernández-Oñate M, Kruszewska J, Lawry R, Mora-Montes H, Muñoz-Centeno T, Nieto-Jacobo M, Nogueira LG, Olmedo-Monfil V, Osorio-Concepcion M, Piłsyk S, Pomraning K, Rodriguez-Iglesias A, Rosales-Saavedra M, Sánchez-Arreguín J, Seidl-Seiboth V, Stewart A, Uresti-Rivera E, Wang C, Wang T, Zeilinger S, Casas-Flores S, Herrera-Estrella A (2016). The genomes of three uneven siblings: footprints of the lifestyles of three Trichoderma species. Microbiol Mol Biol Rev.

[89] PakulaT, Saloheimo M, HÄKKINEN M, Westerholm-Parvinen A, Penttilä M, Vitikainen M. Method for protein production in filamentous fungi. Google Patents. EP Patent App. EP20,110,726,858. 2013. https://google.com/patents/EP2576792A2?cl=en. Accessed 19 May 2016.

[90] Pakula T, Saloheimo M, HÄKKINEN M, Westerholm-Parvinen A, Penttilä M, Vitikainen M. Improved production of proteins in filamentous fungi. Google Patents. EP Patent App. EP20,110,726,860. 2013. http://www.google.com/patents/EP2576794A2?cl=en. Accessed 19 May 2016.

[91] Martinez D, Berka RM, Henrissat B, Saloheimo M, Arvas M, Baker SE, Chapman J, Chertkov O, Coutinho PM, Cullen D, Danchin EGJ, Grigoriev IV, Harris P, Jackson M, Kubicek CP, Han CS, Ho I, Larrondo LF, de Leon AL, Magnuson JK, Merino S, Misra M, Nelson B, Putnam N, Robbertse B, Salamov AA, Schmoll M, Terry A, Thayer N, Westerholm-Parvinen A, Schoch CL, Yao J, Barabote R, Nelson MA, Detter C, Bruce D, Kuske CR, Xie G, Richardson P, Rokhsar DS, Lucas SM, Rubin EM, Dunn-Coleman N, Ward M, Brettin TS (2008). Genome sequencing and analysis of the biomass-degrading fungus Trichoderma reesei (syn. Hypocrea jecorina). Nat Biotechnol.

[92] Arvas M, Kivioja T, Mitchell A, Saloheimo M, Ussery D, Penttilä M, Oliver S (2007). Comparison of protein coding gene contents of the fungal phyla Pezizomycotina and Saccharomycotina. BMC Genomics.

[93] Kontkanen H, Westerholm-Parvinen A, Saloheimo M, Bailey M, Rättö M, Mattila I, Mohsina M, Kalkkinen N, Nakari-Setälä T, Buchert J (2009). Novel coprinopsis cinerea polyesterase that hydrolyzes cutin and suberin. Appl Environ Microbiol.

[94] Bailey MJ, Tätiharju J (2003). Efficient cellulase production by Trichoderma reesei in continuous cultivation on lactose medium with a computer-controlled feeding strategy. Appl Microbiol Biotechnol.

[95] Jiang H, Lei R, Ding S-W, Zhu S (2014). Skewer: a fast and accurate adapter trimmer for next-generation sequencing paired-end reads. BMC Bioinformatics.

[96] Kersey PJ, Allen JE, Armean I, Boddu S, Bolt BJ, Carvalho-Silva D, Christensen M, Davis P, Falin LJ, Grabmueller C, Humphrey J, Kerhornou A, Khobova J, Aranganathan NK, Langridge N, Lowy E, McDowall MD, Maheswari U, Nuhn M, Ong CK, Overduin B, Paulini M, Pedro H, Perry E, Spudich G, Tapanari E, Walts B, Williams G, Tello-Ruiz M, Stein J, Wei S, Ware D, Bolser DM, Howe KL, Kulesha E, Lawson D, Maslen G, Staines DM (2015). Ensembl genomes 2016: more genomes more complexity. Nucleic Acids Res.

[97] Lawrence M, Huber W, Pagè H, Aboyoun P, Carlson M, Gentleman R, Morgan MT, Carey VJ (2013). Software for computing and annotating genomic ranges. PLoS Comput Biol.

[98] Falcon S, Gentleman R (2006). Using GOstats to test gene lists for GO term association. Bioinformatics.

[99] Pages H, Carlson M, FalconS, Li N. AnnotationDbi: annotation database interface. R package version 1.28.2.

[100] Storey JD, Taylor JE, Siegmund D (2004). Strong control conservative point estimation and simultaneous conservative consistency of false discovery rates: a unified approach. J R Stat Soc Ser B Stat Methodol.

[101] Shen L, Sinai M. GeneOverlap: test and visualize gene overlaps. R package version 1.2.0. 2013. http://www.shenlab-sinai.github.io/shenlab-sinai/. Accessed 19 May 2016.

[102] Weng S (2003). Saccharomyces genome database (SGD) provides biochemical and structural information for budding yeast proteins. Nucleic Acids Res.

[103] Gelius-Dietrich G, Desouki A, Fritzemeier C, Lercher MJ (2013). Sybil—efficient constraint-based modelling in R. BMC Syst Biol.

[104] Caspeta L, Shoaie S, Agren R, Nookaew I, Nielsen J (2012). Genome-scale metabolic reconstructions of Pichia stipitis and Pichia pastoris and in-silico evaluation of their potentials. BMC Syst Biol.

[105] Kanehisa M, Goto S, Sato Y, Kawashima M, Furumichi M, Tanabe M (2013). Data information, knowledge and principle: back to metabolism in KEGG. Nucleic Acids Res.

[106] Caspi R, Billington R, Ferrer L, Foerster H, Fulcher CA, Keseler IM, Kothari A, Krummenacker M, Latendresse M, Mueller LA, Ong Q, Paley S, Subhraveti P, Weaver DS, Karp PD (2015). The MetaCyc database of metabolic pathways and enzymes and the BioCyc collection of pathway/genome databases. Nucleic Acids Res.

[107] Kanehisa M, Goto S (2000). Kegg: kyoto encyclopedia of genes and genomes. Nucleic Acids Res.

[108] Arvas M, Pakula T, Smit B, Rautio J, Koivistoinen H, Jouhten P, Lindfors E, Wiebe M, Penttilä M, Saloheimo M (2011). Correlation of gene expression and protein production rate—a system wide study. BMC Genomics.

