# Supplementary material for: Genome wide analysis of protein production load in Trichoderma reesei
Source: Biotechnol Biofuels. 2016 Jun 28;9:132. doi: 10.1186/s13068-016-0547-5 (PMC4924338; doi:10.1186/s13068-016-0547-5)

Supplementary figures for “Genome wide analysis of protein production load in Trichoderma reesei”

Contents

1. Lipase production 2

2. Ethanol production 2

3. Intracellular free amino acid concentrations 3

4. Gene expression clusters 13

5. Correlations of genes and cultivation parameters 15

6. Overlap of gene expression clusters and lists of significantly changing genes 17

7. CAZY and related genes known to be highly produced based on proteomics 18

8. FIRE analysis for detecting shared promoter motifs in gene expression clusters 20

9. Comparison of gene’s correlation to specific protein production rate in this publication and in Arvas 2011 “Correlation of gene expression and protein production rate - a system wide study” 22

10. Flux clusters 23

11. Glycerol identification 24

12. Cellotriose identification 25

13. Fits of heteroscedastic Gaussian processes 26

    A. Biomass i.e. CDW (g/l) 26

    B. Extracellular protein (g/l) 27

    C. Cellobiose (g/l) 27

    D. MUL (nkat/l) 28

    E. Cellotriose (g/l) 28

    F. Glucose (g/l) 29

    G. Glycerol (g/l) 29

1. **Lipase production**

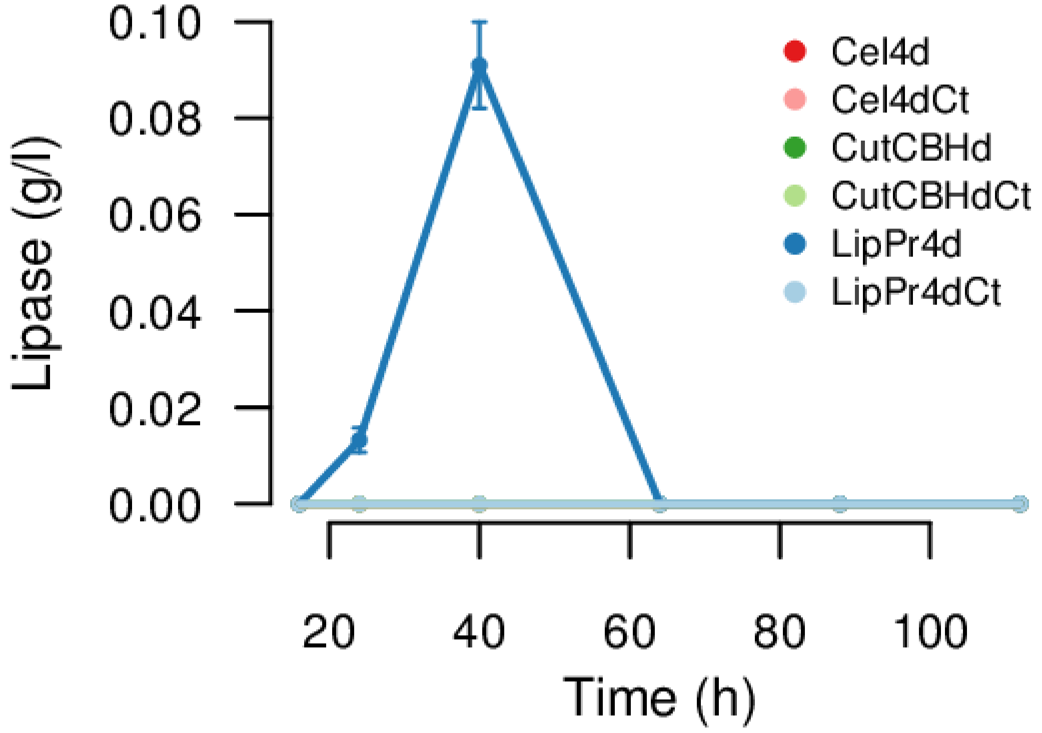

2. **Ethanol production**

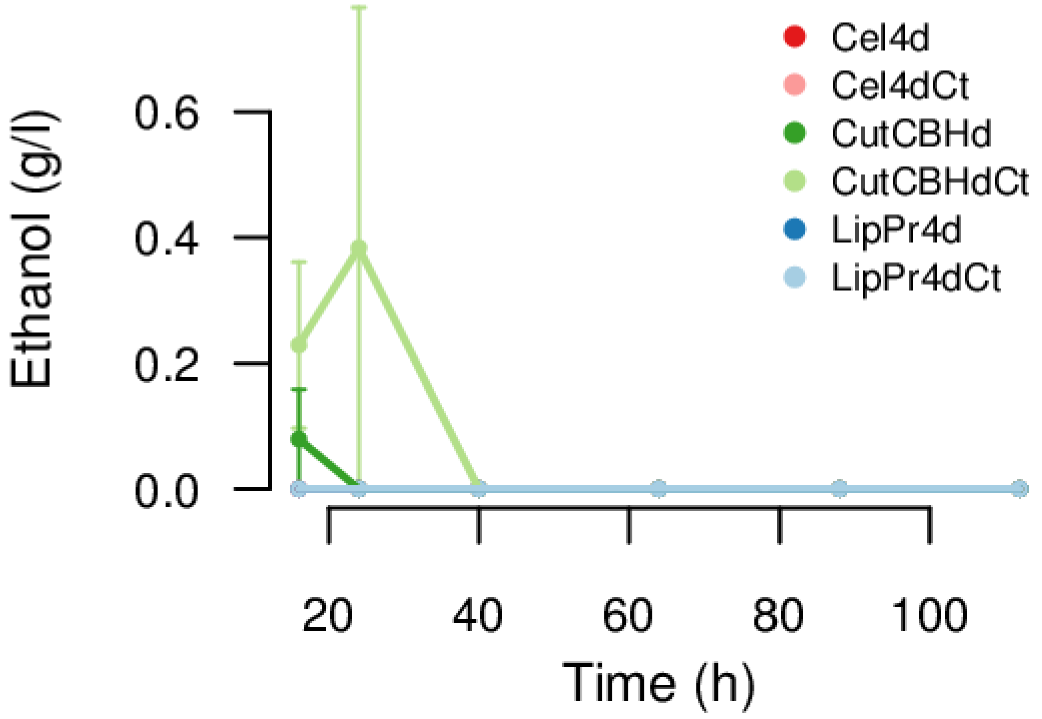

3. Intracellular free amino acid concentrations

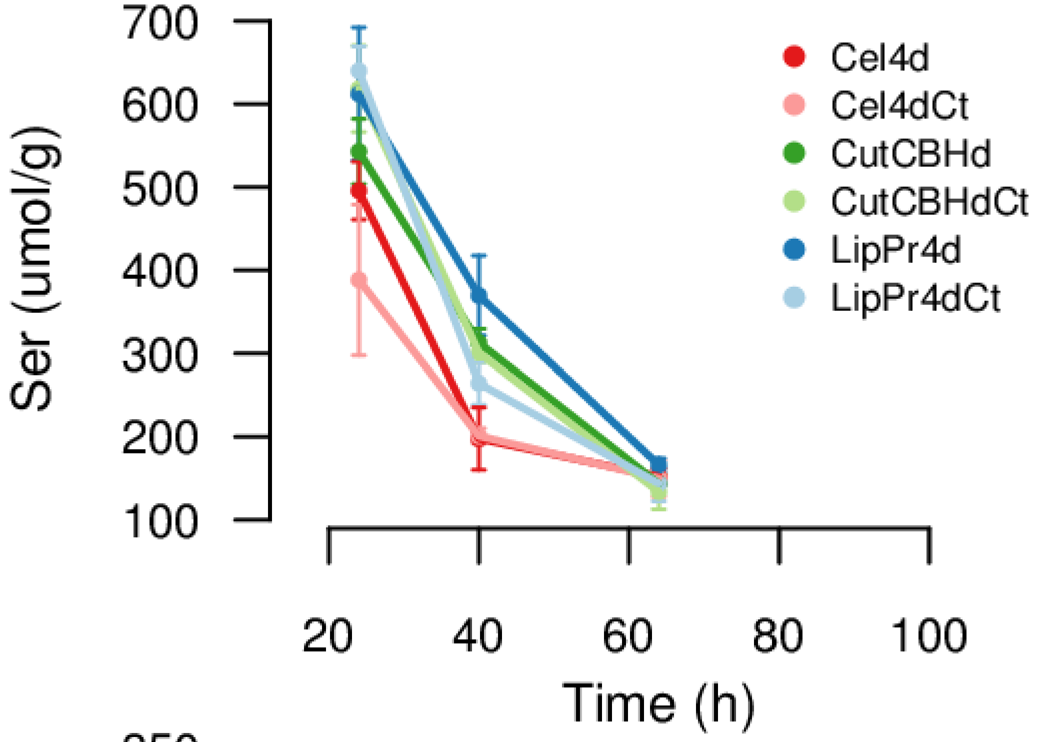

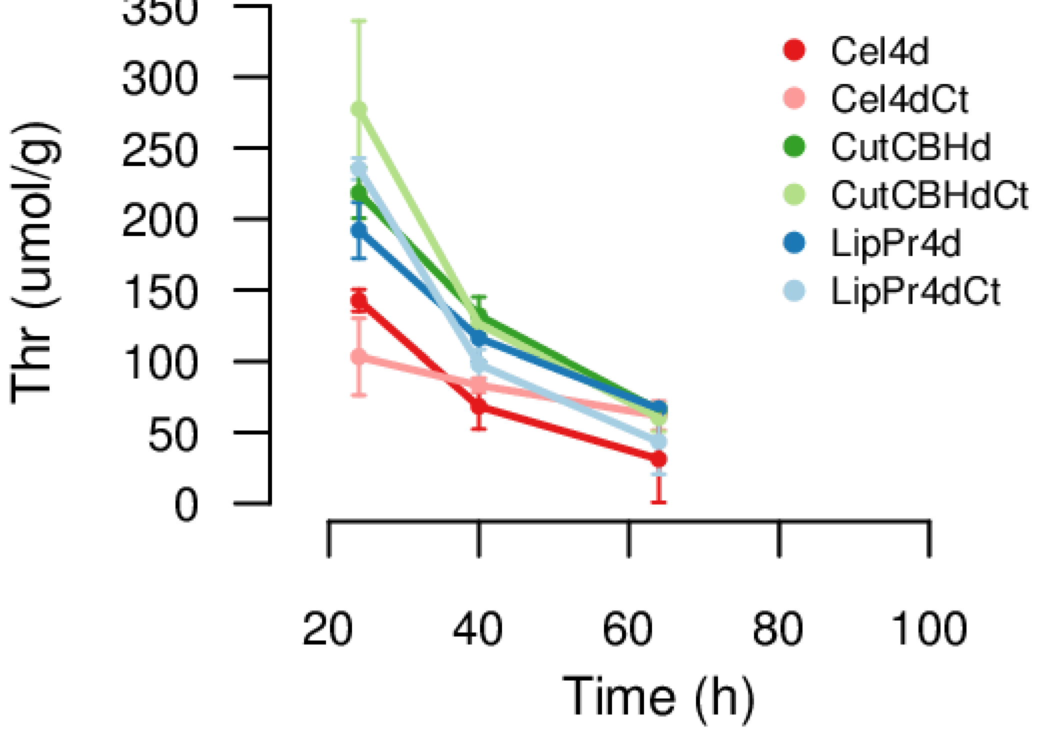

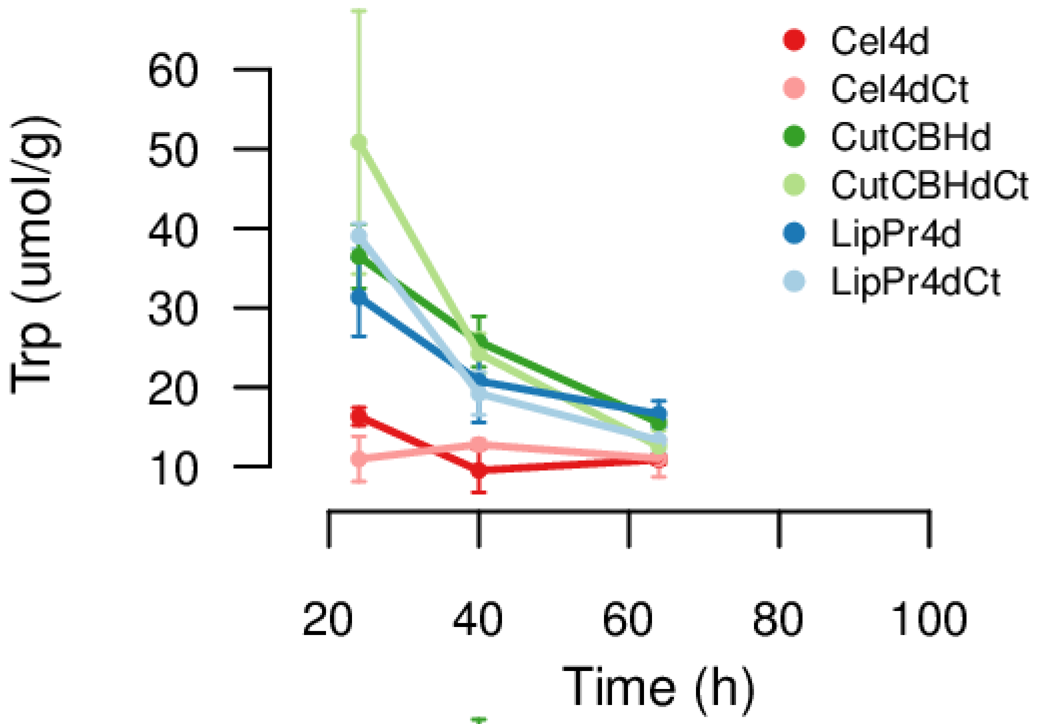

Trp (umol/g)
60
50
40
30
20
10
20
40
60
80
100
Time (h)
Cel4d
Cel4dCt
CutCBHd
CutCBHdCt
LipPr4d
LipPr4dCt

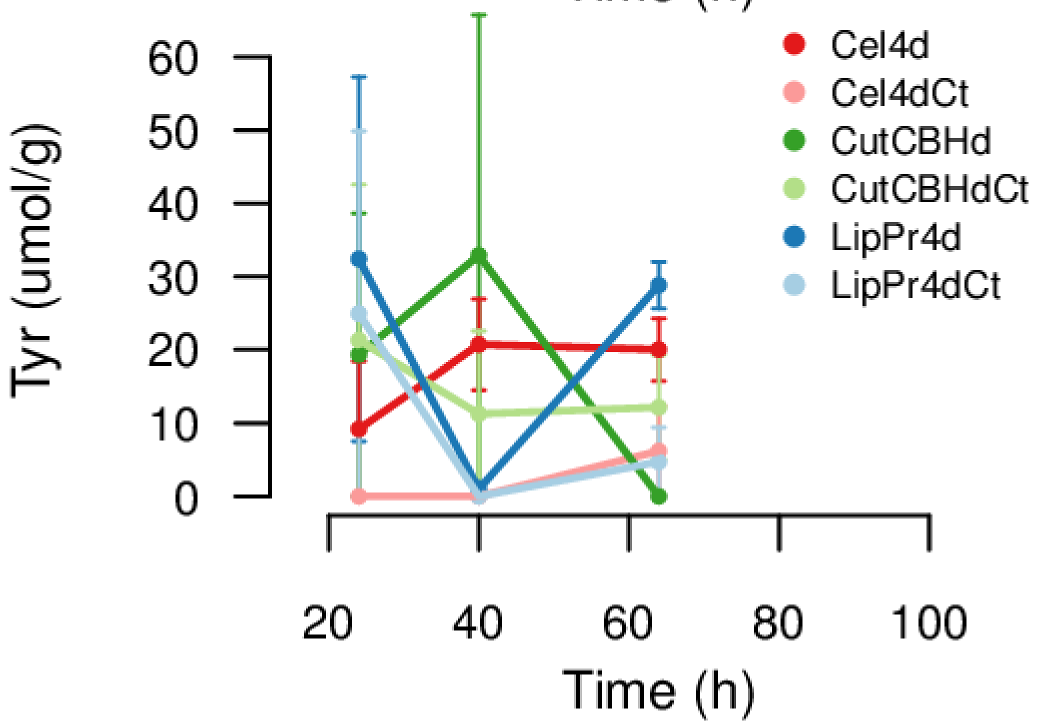

Tyr (umol/g)
60
50
40
30
20
10
0
20
40
60
80
100
Time (h)
Cel4d
Cel4dCt
CutCBHd
CutCBHdCt
LipPr4d
LipPr4dCt

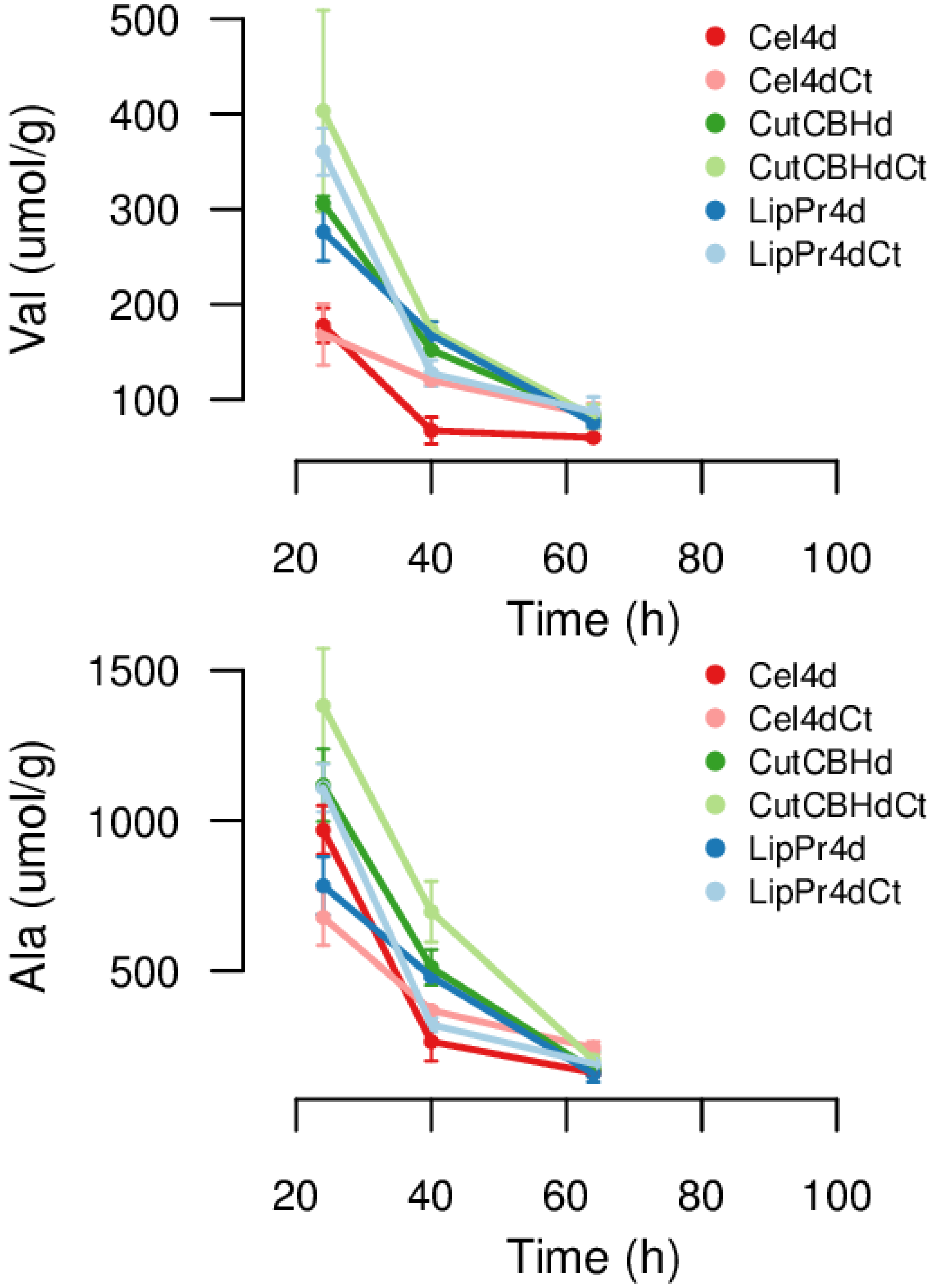

Val (umol/g)
500
400
300
200
100
20
40
60
80
100
Time (h)
Cel4d
Cel4dCt
CutCBHd
CutCBHdCt
LipPr4d
LipPr4dCt
Ala (umol/g)
1500
1000
500
20
40
60
80
100
Time (h)
Cel4d
Cel4dCt
CutCBHd
CutCBHdCt
LipPr4d
LipPr4dCt

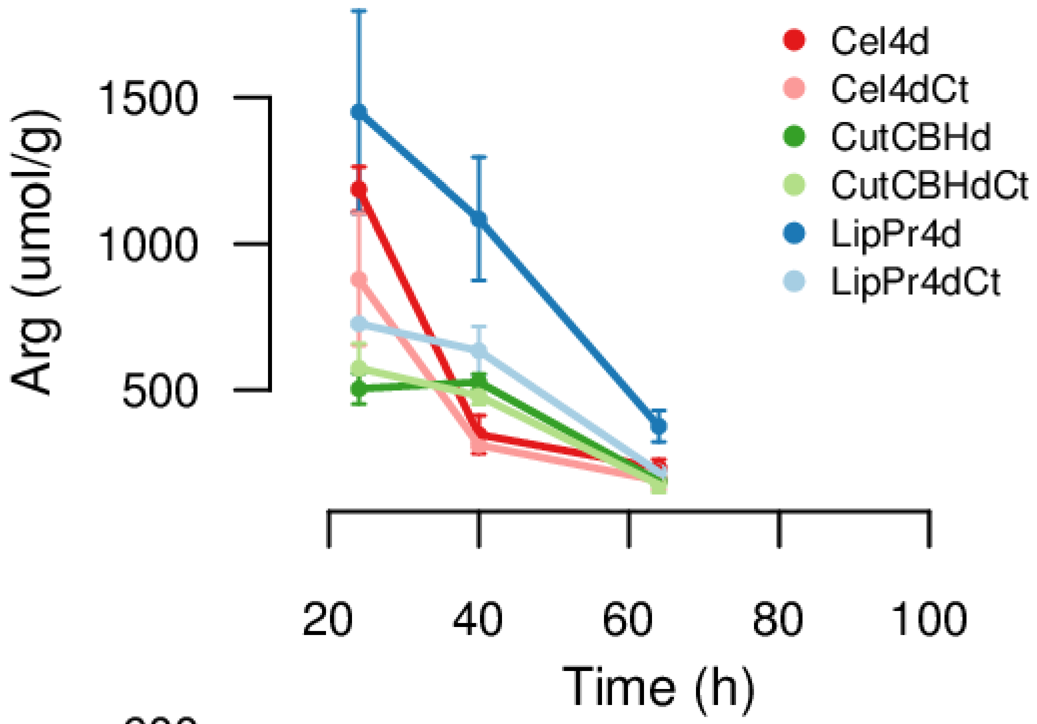
Arg (umol/g)
1500
1000
500
20
40
60
80
100
Time (h)
Cel4d
Cel4dCt
CutCBHd
CutCBHdCt
LipPr4d
LipPr4dCt

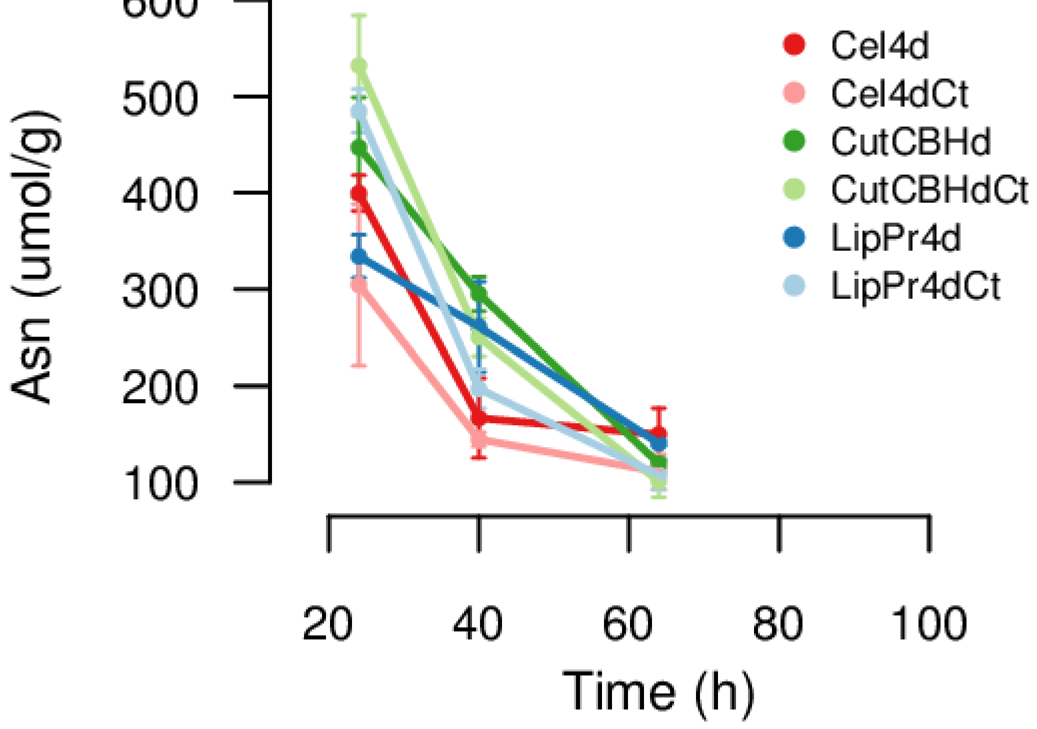
Asn (umol/g)
600
500
400
300
200
100
20
40
60
80
100
Time (h)
Cel4d
Cel4dCt
CutCBHd
CutCBHdCt
LipPr4d
LipPr4dCt

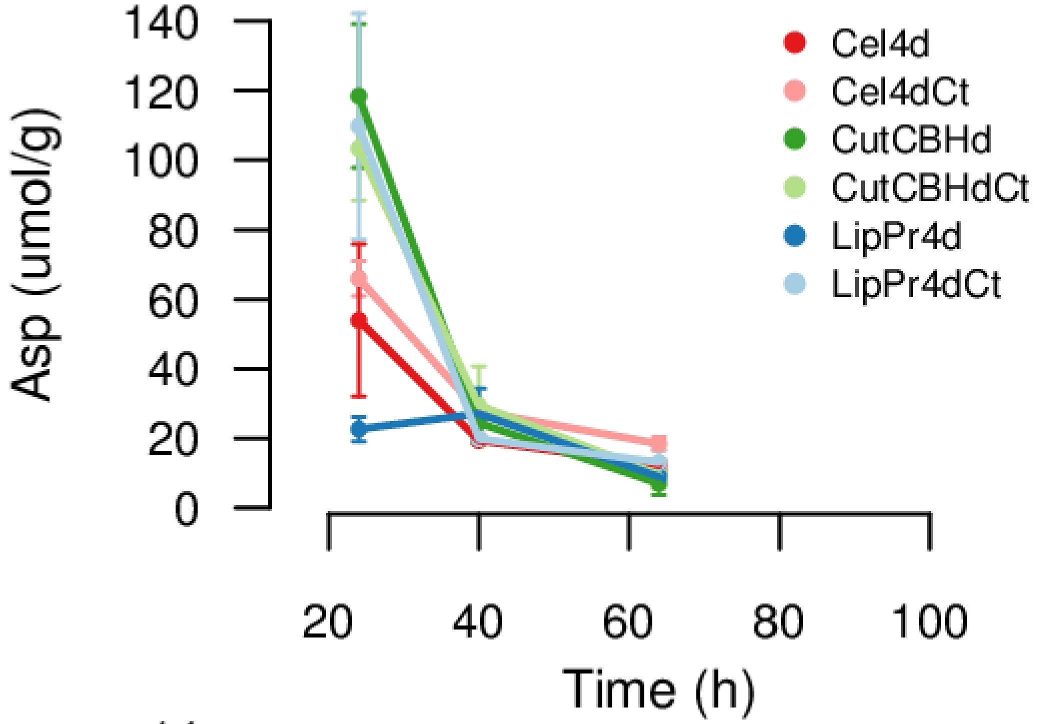
Asp (umol/g)
Time (h)
Cel4d
Cel4dCt
CutCBHd
CutCBHdCt
LipPr4d
LipPr4dCt

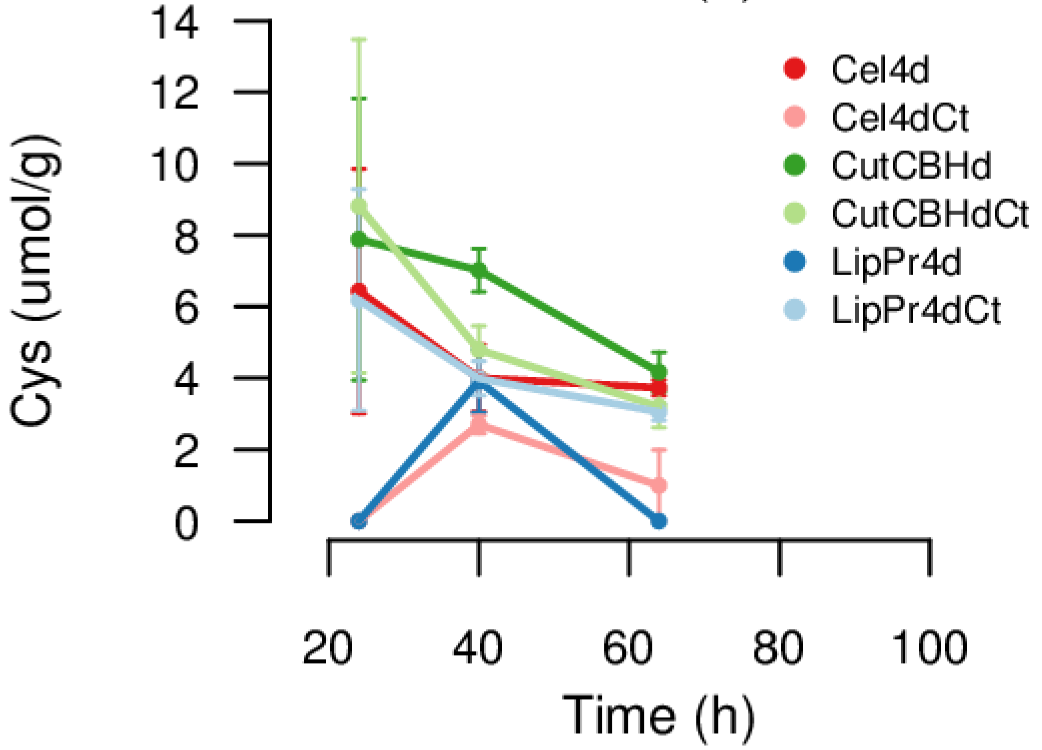
Cys (umol/g)
Time (h)
Cel4d
Cel4dCt
CutCBHd
CutCBHdCt
LipPr4d
LipPr4dCt

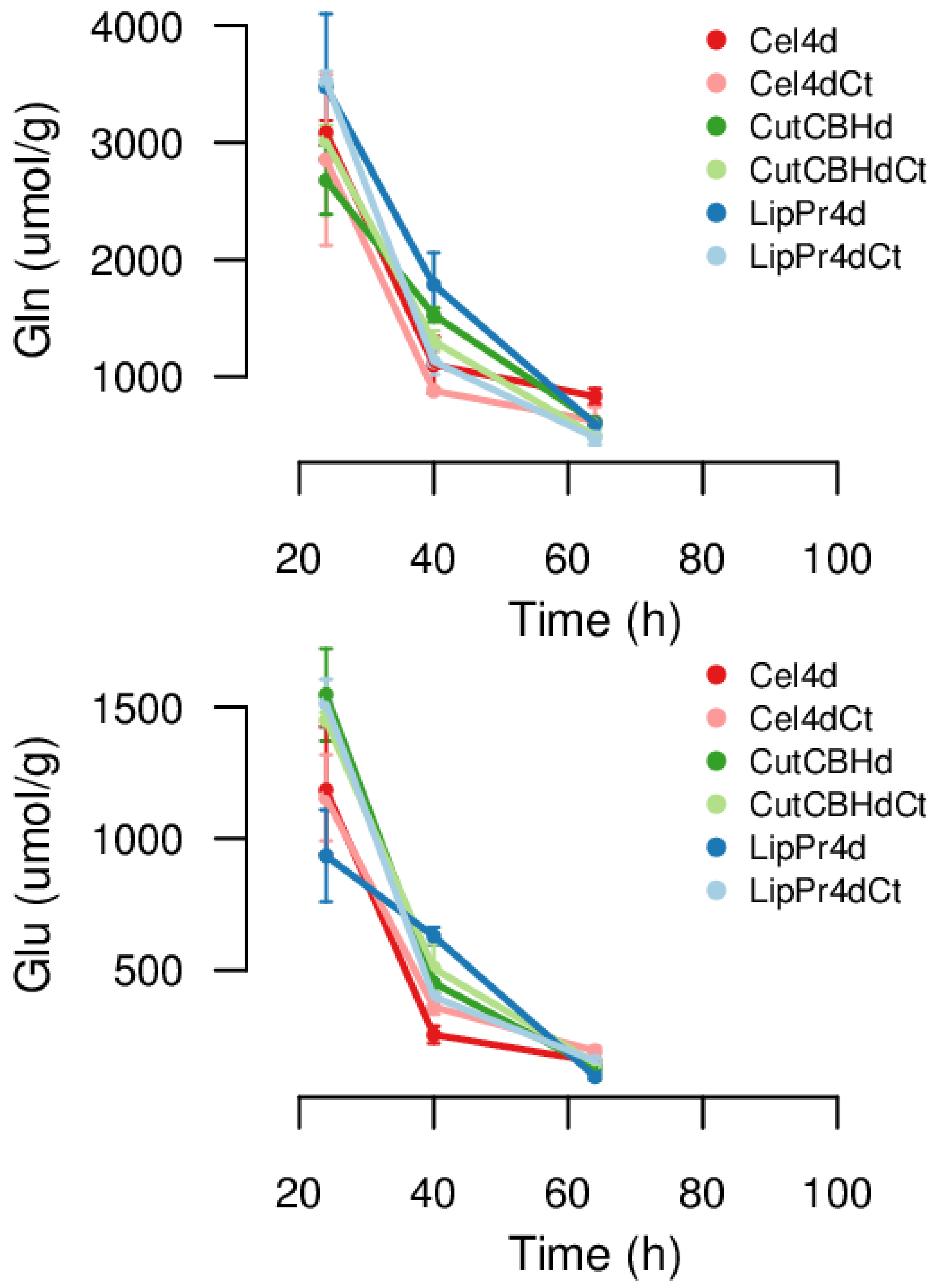
Gln (umol/g)
4000
3000
2000
1000
20
40
60
80
100
Time (h)
Cel4d
Cel4dCt
CutCBHd
CutCBHdCt
LipPr4d
LipPr4dCt
Glu (umol/g)
1500
1000
500
20
40
60
80
100
Time (h)
Cel4d
Cel4dCt
CutCBHd
CutCBHdCt
LipPr4d
LipPr4dCt

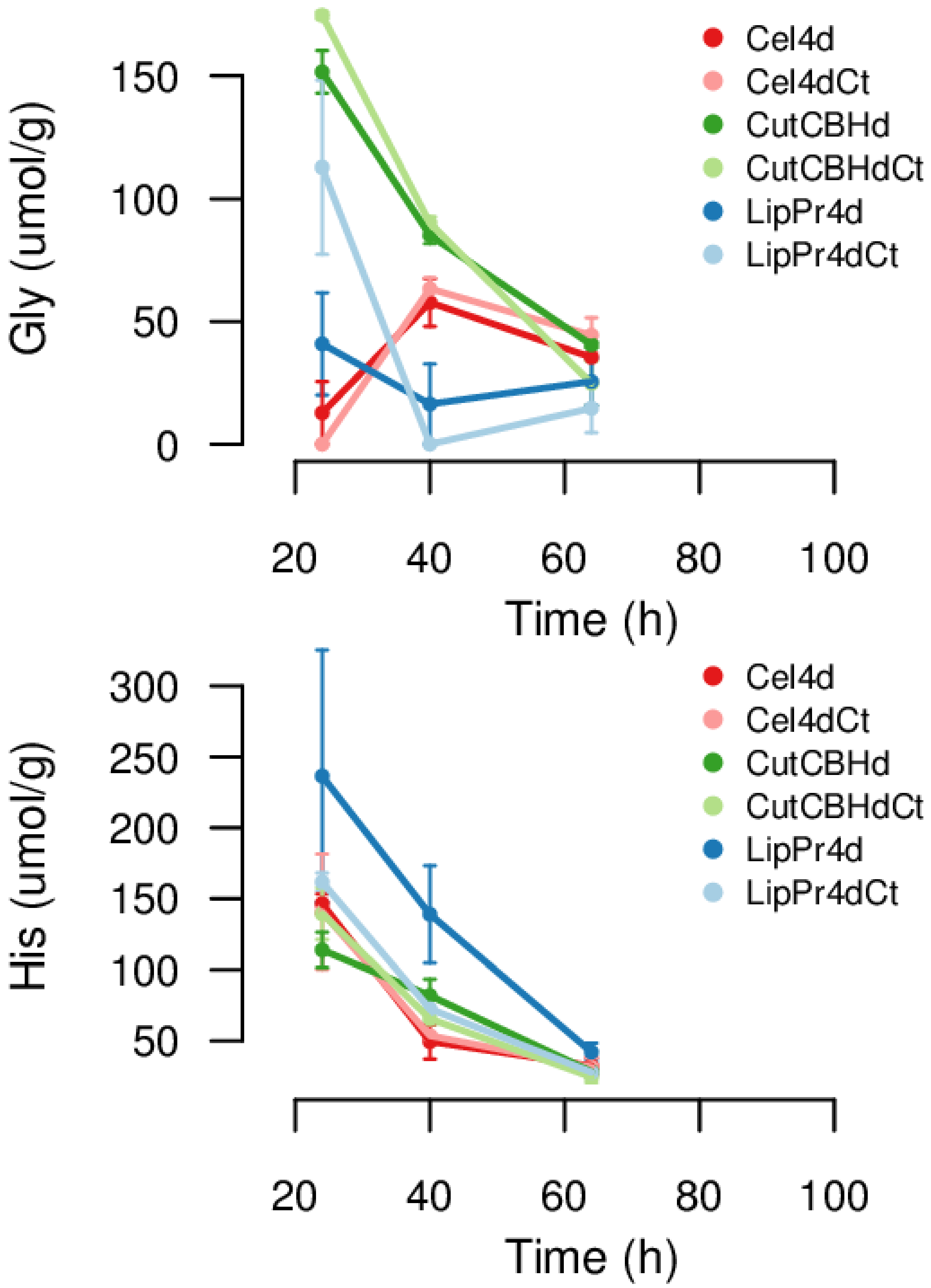
Gly (umol/g)
150
100
50
0
20
40
60
80
100
Time (h)
Cel4d
Cel4dCt
CutCBHd
CutCBHdCt
LipPr4d
LipPr4dCt
His (umol/g)
300
250
200
150
100
50
20
40
60
80
100
Time (h)
Cel4d
Cel4dCt
CutCBHd
CutCBHdCt
LipPr4d
LipPr4dCt

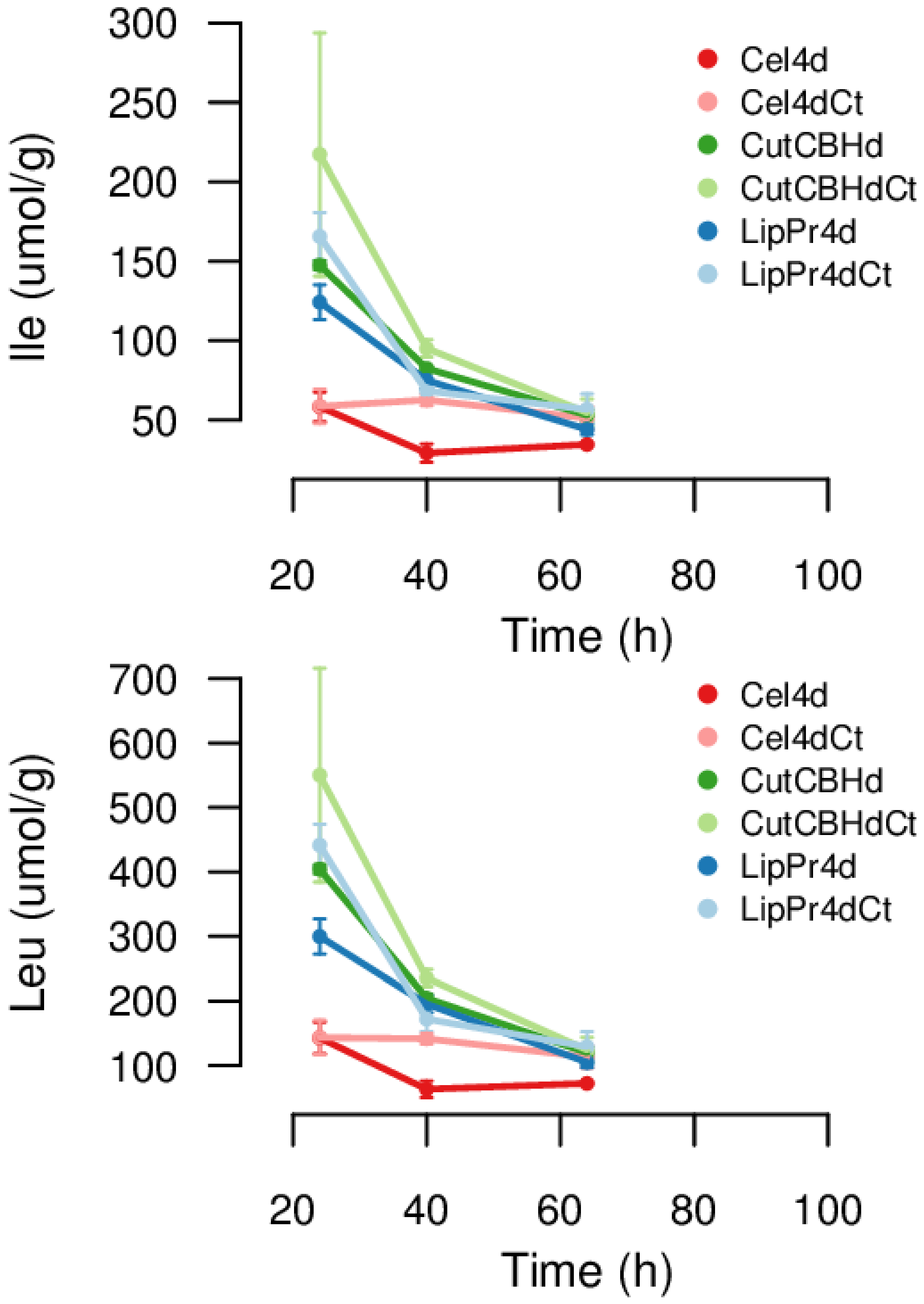
Ile (umol/g)
300
250
200
150
100
50
20
40
60
80
100
Time (h)
Cel4d
Cel4dCt
CutCBHd
CutCBHdCt
LipPr4d
LipPr4dCt
Leu (umol/g)
700
600
500
400
300
200
100
20
40
60
80
100
Time (h)
Cel4d
Cel4dCt
CutCBHd
CutCBHdCt
LipPr4d
LipPr4dCt

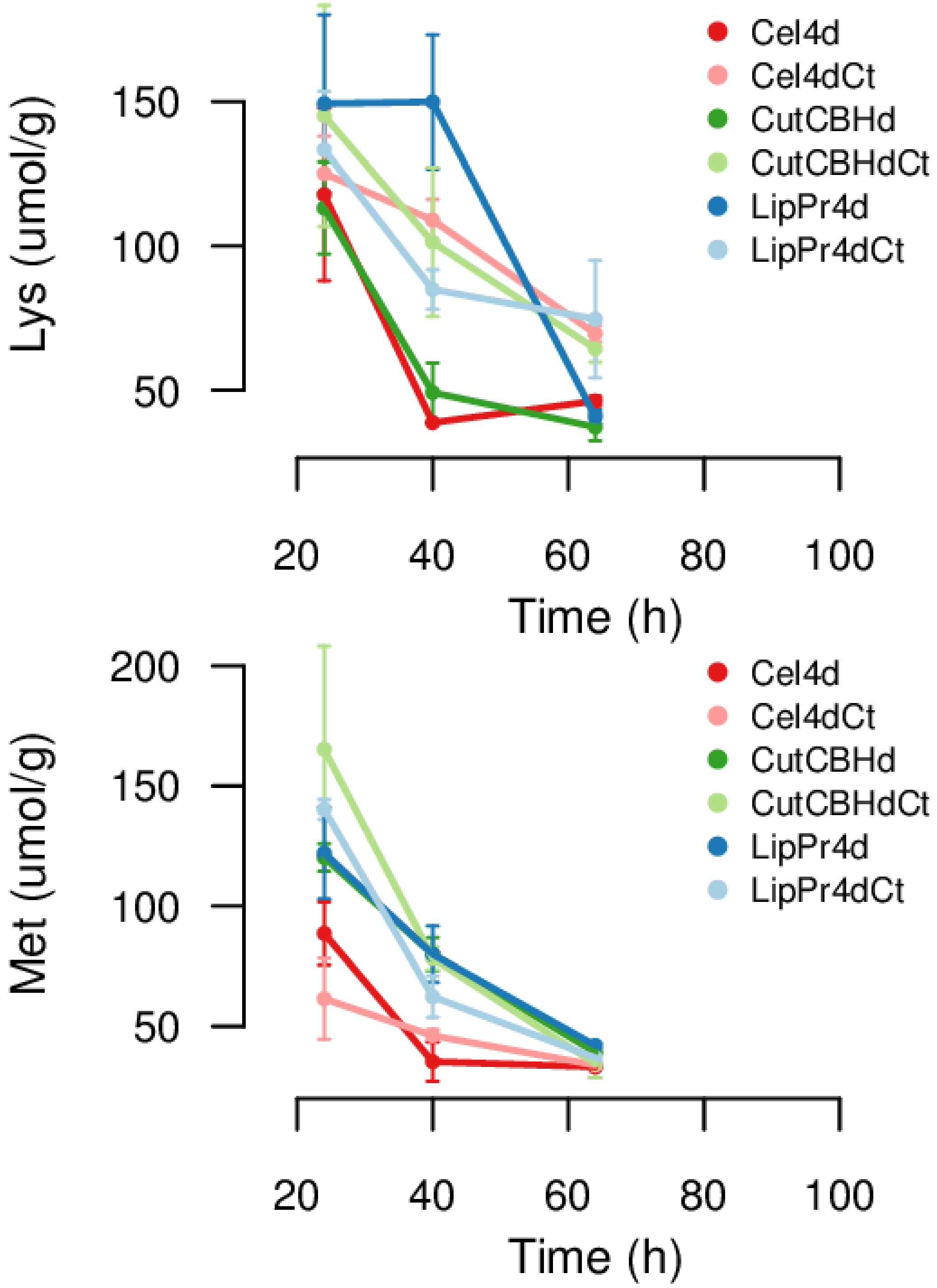
Lys (umol/g)
150
100
50
20
40
60
80
100
Time (h)
Cel4d
Cel4dCt
CutCBHd
CutCBHdCt
LipPr4d
LipPr4dCt
Met (umol/g)
200
150
100
50
20
40
60
80
100
Time (h)
Cel4d
Cel4dCt
CutCBHd
CutCBHdCt
LipPr4d
LipPr4dCt

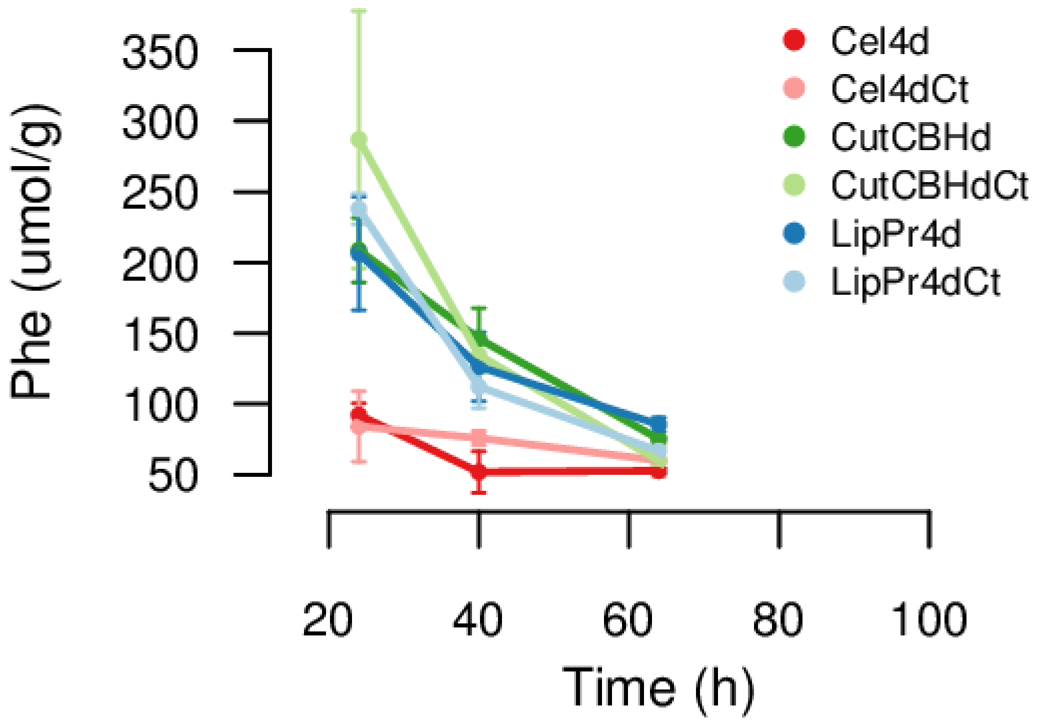
Phe (umol/g)
350
300
250
200
150
100
50
20
40
60
80
100
Time (h)
Cel4d
Cel4dCt
CutCBHd
CutCBHdCt
LipPr4d
LipPr4dCt

4. Gene expression clusters

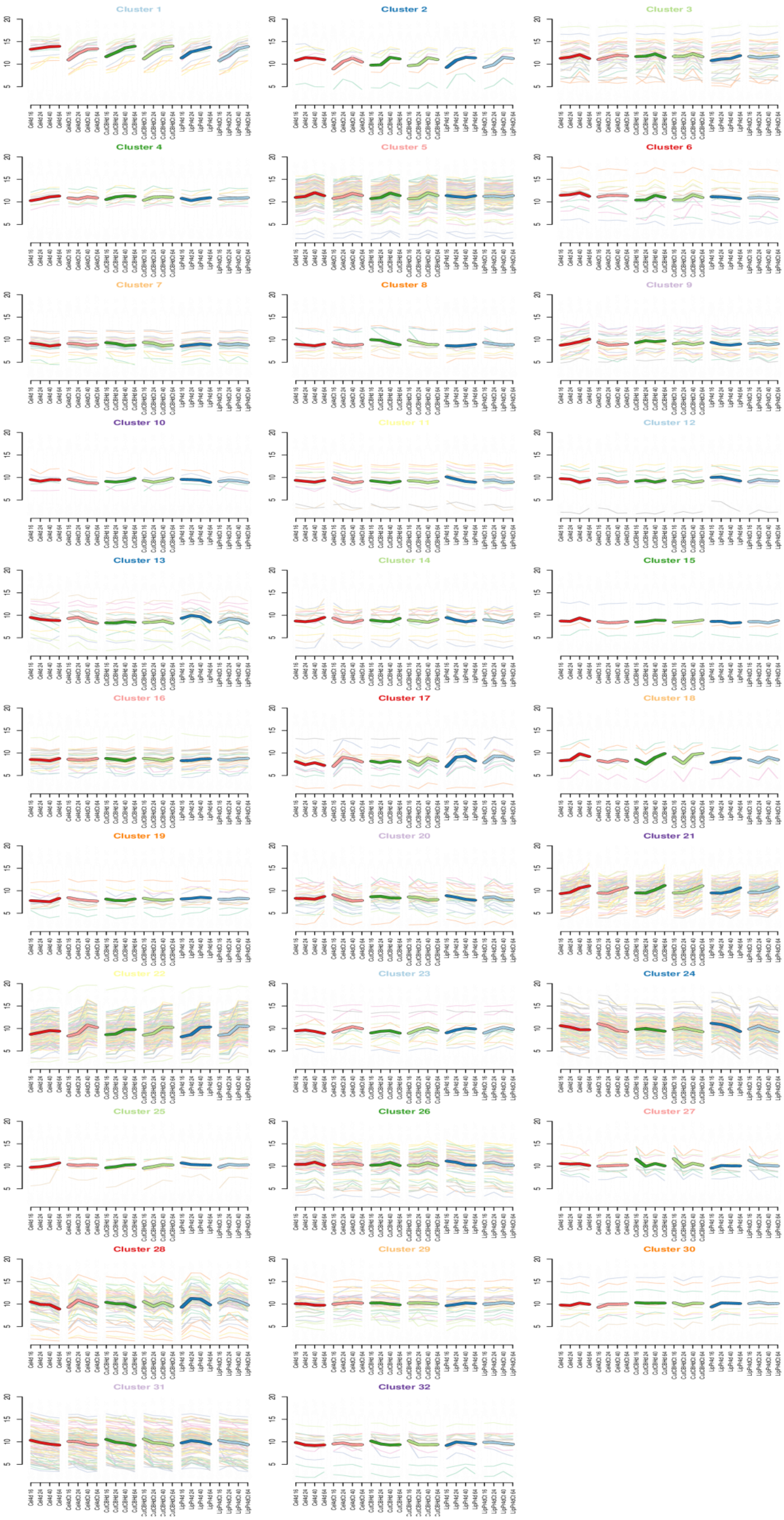
Cluster 1
Cluster 2
Cluster 3
Cluster 4
Cluster 5
Cluster 6
Cluster 7
Cluster 8
Cluster 9
Cluster 10
Cluster 11
Cluster 12
Cluster 13
Cluster 14
Cluster 15
Cluster 16
Cluster 17
Cluster 18
Cluster 19
Cluster 20
Cluster 21
Cluster 22
Cluster 23
Cluster 24
Cluster 25
Cluster 26
Cluster 27
Cluster 28
Cluster 29
Cluster 30
Cluster 31
Cluster 32

5. Correlations of genes and cultivation parameters

20 genes with lowest correlation above the FDR cut-off of 0.00005. Y-axes cultivation parameter, X-axes gene expression (rlog2). Title, the un-adjusted p-value of correlation and the correlation.

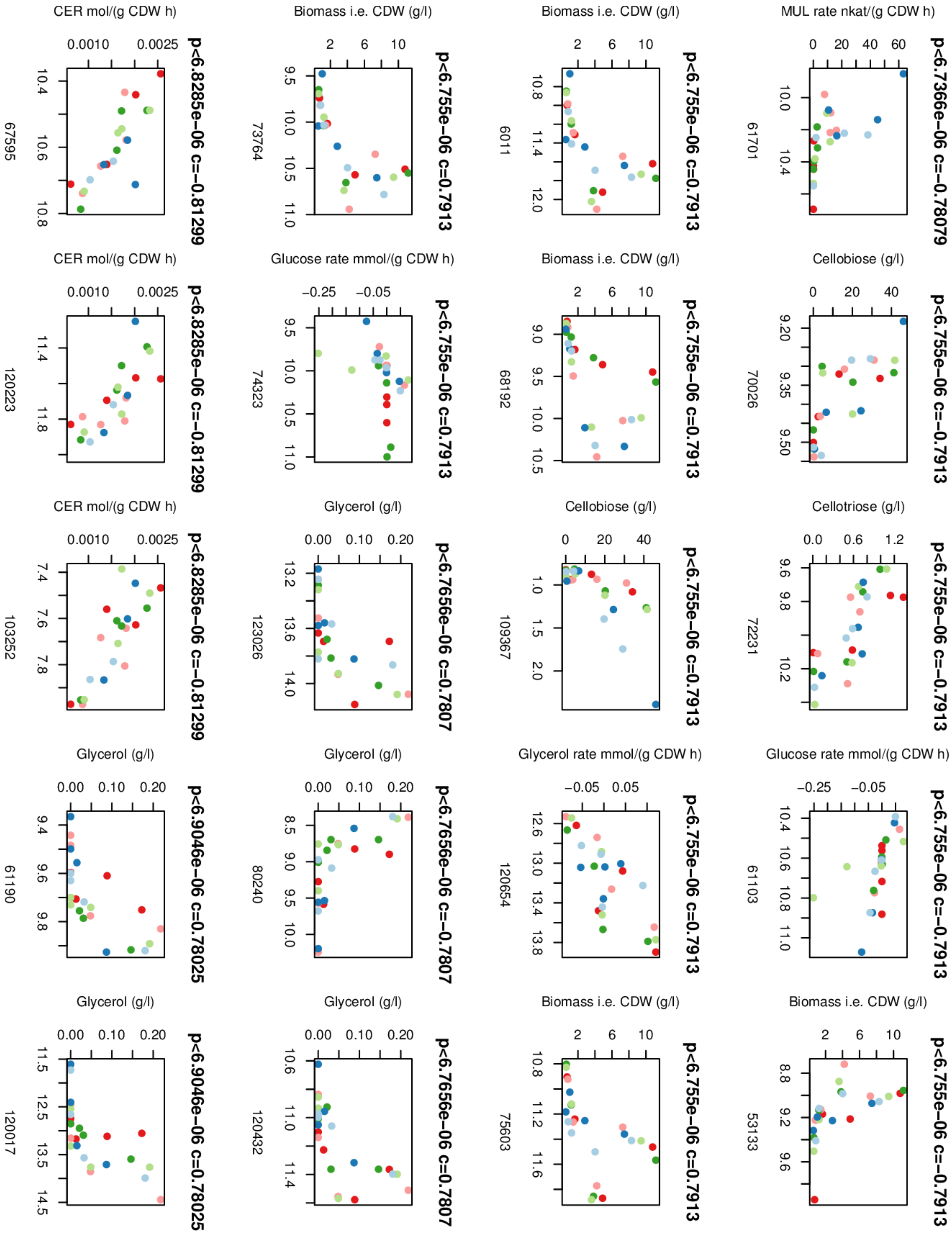
CER mol/(g CDW h)
p<6.8285e−06 c=−0.81299
67595
Biomass i.e. CDW (g/l)
p<6.755e−06 c=0.7913
73764
Biomass i.e. CDW (g/l)
p<6.755e−06 c=0.7913
6011
MUL rate nkat/(g CDW h)
p<6.7366e−06 c=−0.78079
61701
CER mol/(g CDW h)
p<6.8285e−06 c=−0.81299
120223
Glucose rate mmol/(g CDW h)
p<6.755e−06 c=0.7913
74323
Biomass i.e. CDW (g/l)
p<6.755e−06 c=0.7913
68192
Cellobiose (g/l)
p<6.755e−06 c=−0.7913
70026
CER mol/(g CDW h)
p<6.8285e−06 c=−0.81299
103252
Glycerol (g/l)
p<6.7656e−06 c=0.7807
123026
Cellobiose (g/l)
p<6.755e−06 c=0.7913
109367
Cellotriose (g/l)
p<6.755e−06 c=0.7913
72231
Glycerol (g/l)
p<6.9046e−06 c=0.78025
61190
Glycerol (g/l)
p<6.7656e−06 c=−0.7807
80240
Glycerol rate mmol/(g CDW h)
p<6.755e−06 c=0.7913
120654
Glucose rate mmol/(g CDW h)
p<6.755e−06 c=−0.7913
61103
Glycerol (g/l)
p<6.9046e−06 c=0.78025
120017
Glycerol (g/l)
p<6.7656e−06 c=0.7807
120432
Biomass i.e. CDW (g/l)
p<6.755e−06 c=0.7913
75603
Biomass i.e. CDW (g/l)
p<6.755e−06 c=0.7913
53133

6. Overlap of gene expression clusters and lists of significantly changing genes

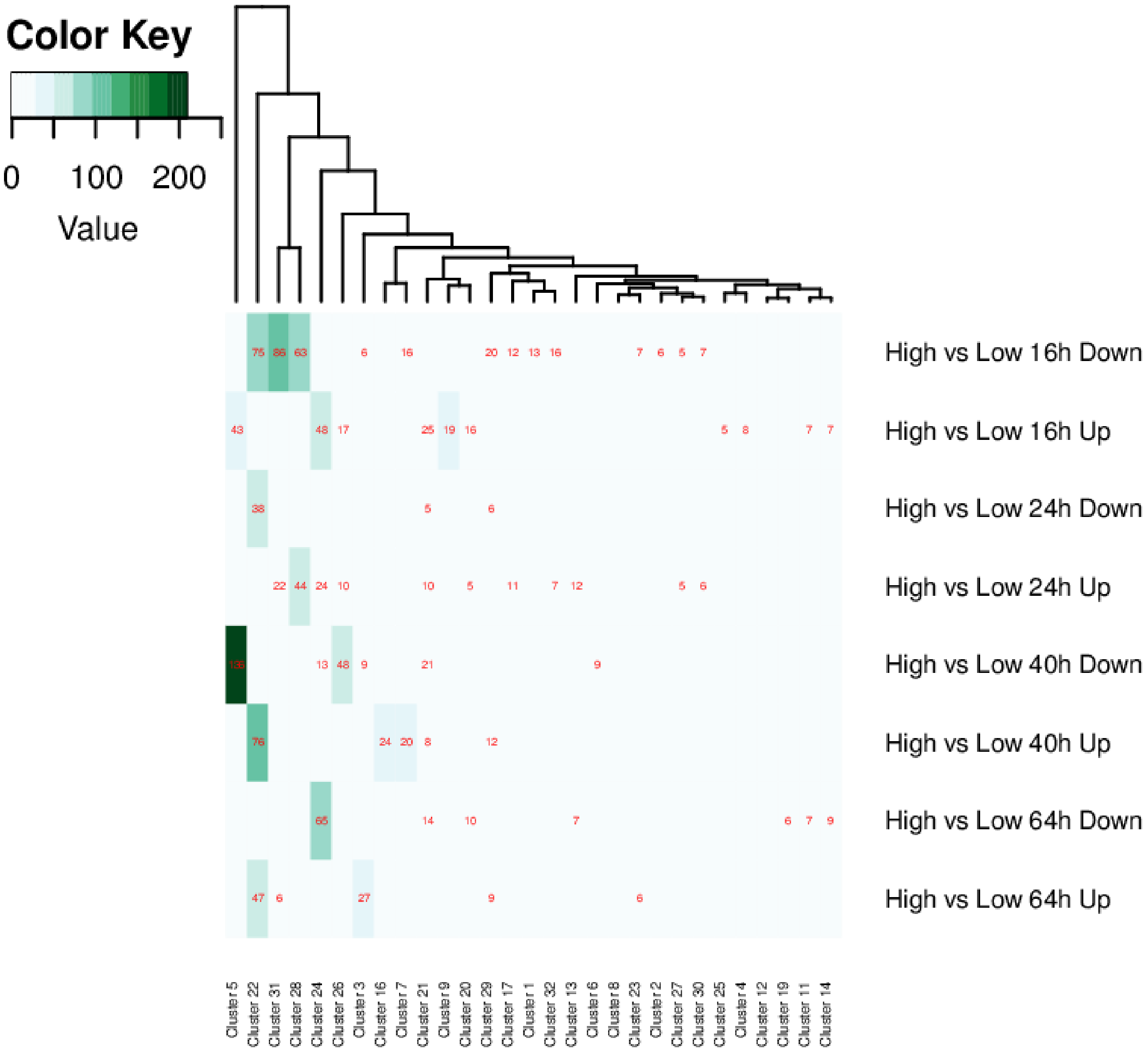

7. CAZY and related genes known to be highly produced based on proteomics

Gene expression as rlog2 value.

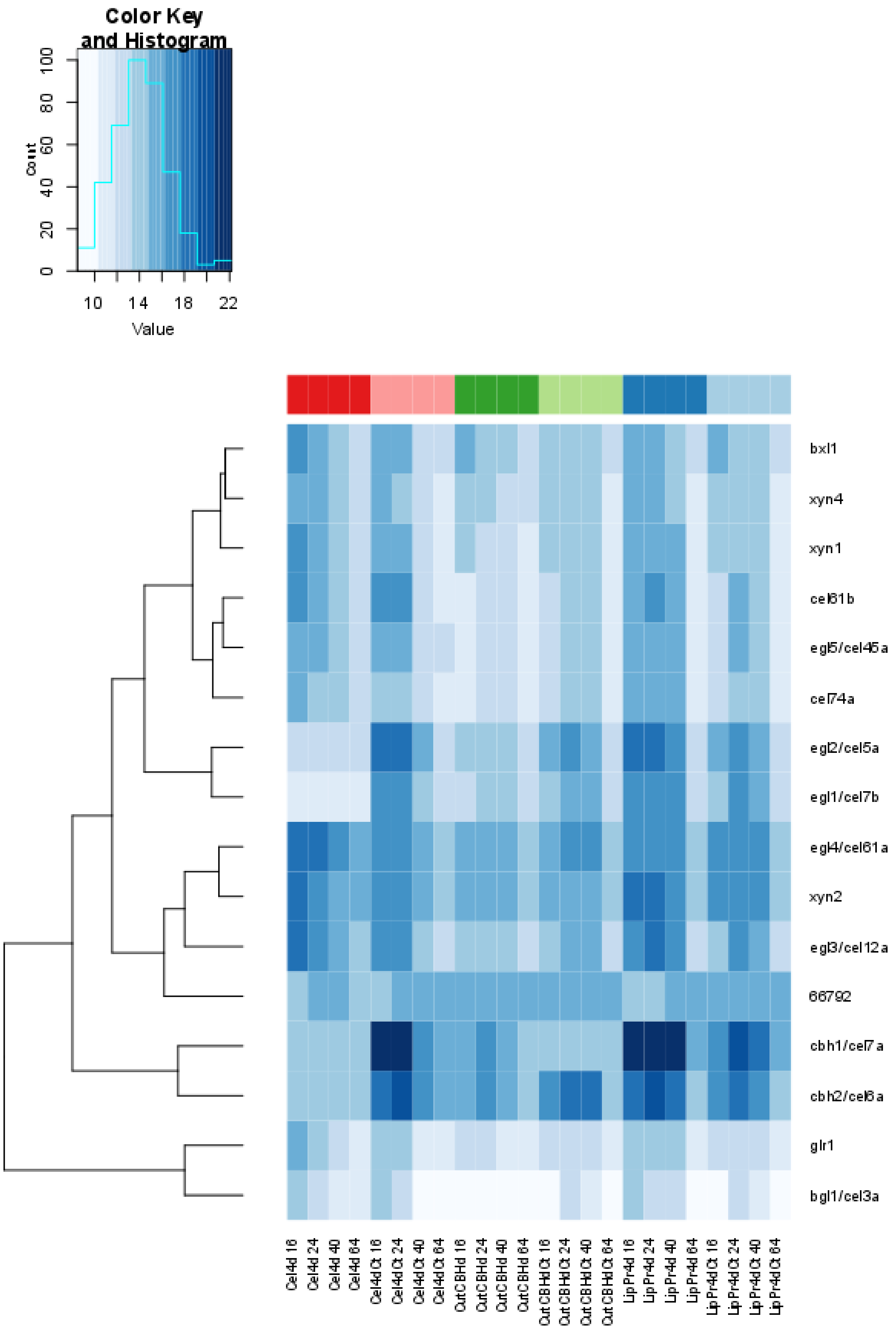

8. FIRE analysis for detecting shared promoter motifs in gene expression clusters

Motif 1:

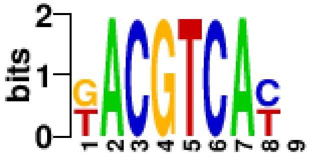

Motif 2:

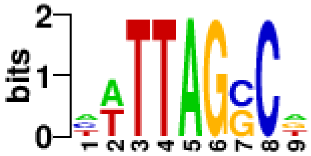

Motif 3:

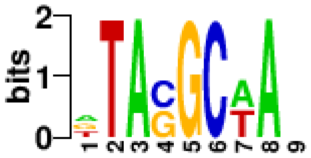

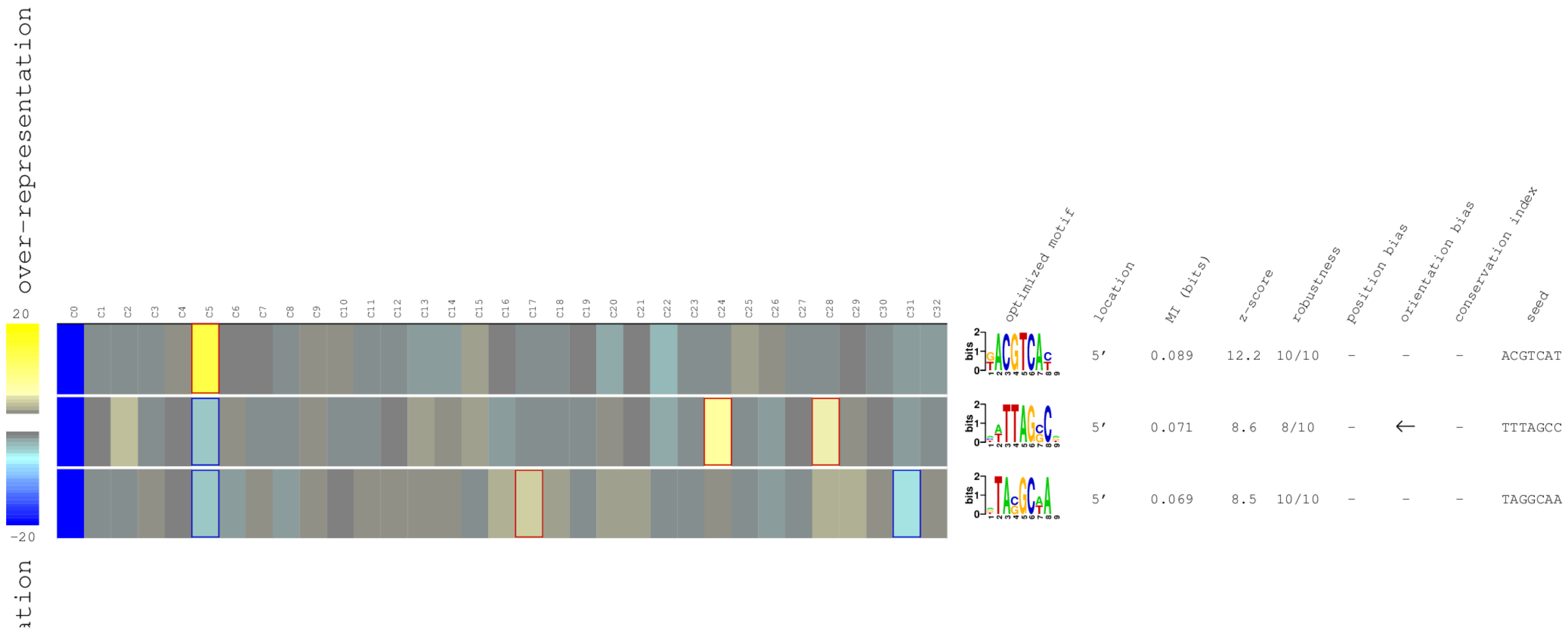

over-representation
20
-20
C0 C1 C2 C3 C4 C5 C6 C7 C8 C9 C10 C11 C12 C13 C14 C15 C16 C17 C18 C19 C20 C21 C22 C23 C24 C25 C26 C27 C28 C29 C30 C31 C32
optimized motif
location
MI (bits)
z-score
robustness
position bias
orientation bias
conservation index
seed
5′ 0.089 12.2 10/10 – – – ACGTCAT
5′ 0.071 8.6 8/10 – ← – TTTAGCC
5′ 0.069 8.5 10/10 – – – TAGGCAA

9. Comparison of gene's correlation to specific protein production rate in this publication and in Arvas 2011 "Correlation of gene expression and protein production rate - a system wide study"

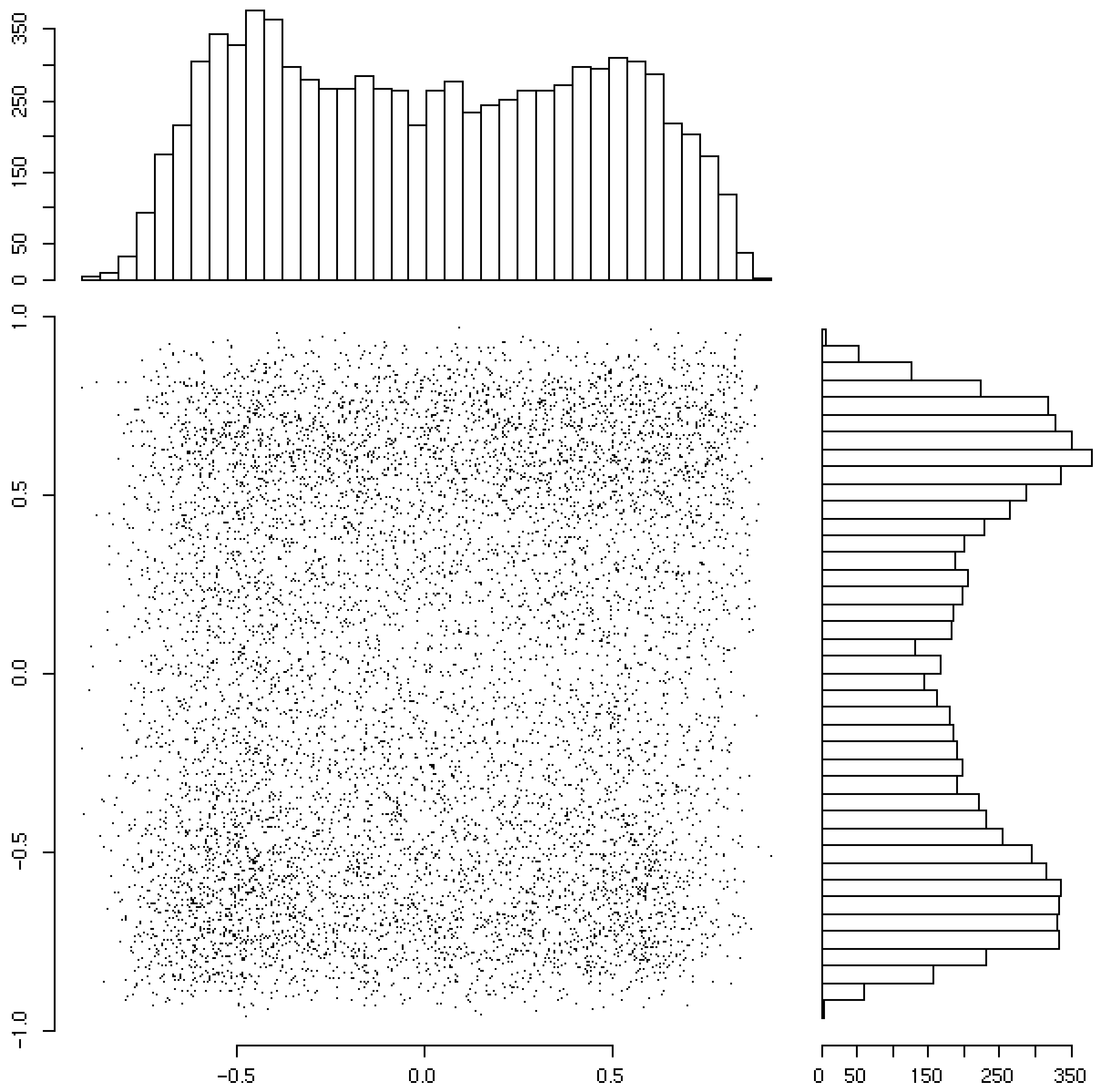

On y-axes the gene expression's correlation to specific protein production rate in this publication. On x-axes gene expression's correlation to specific protein production rate in Arvas 2011. Each dot is a single gene.

10. Flux clusters

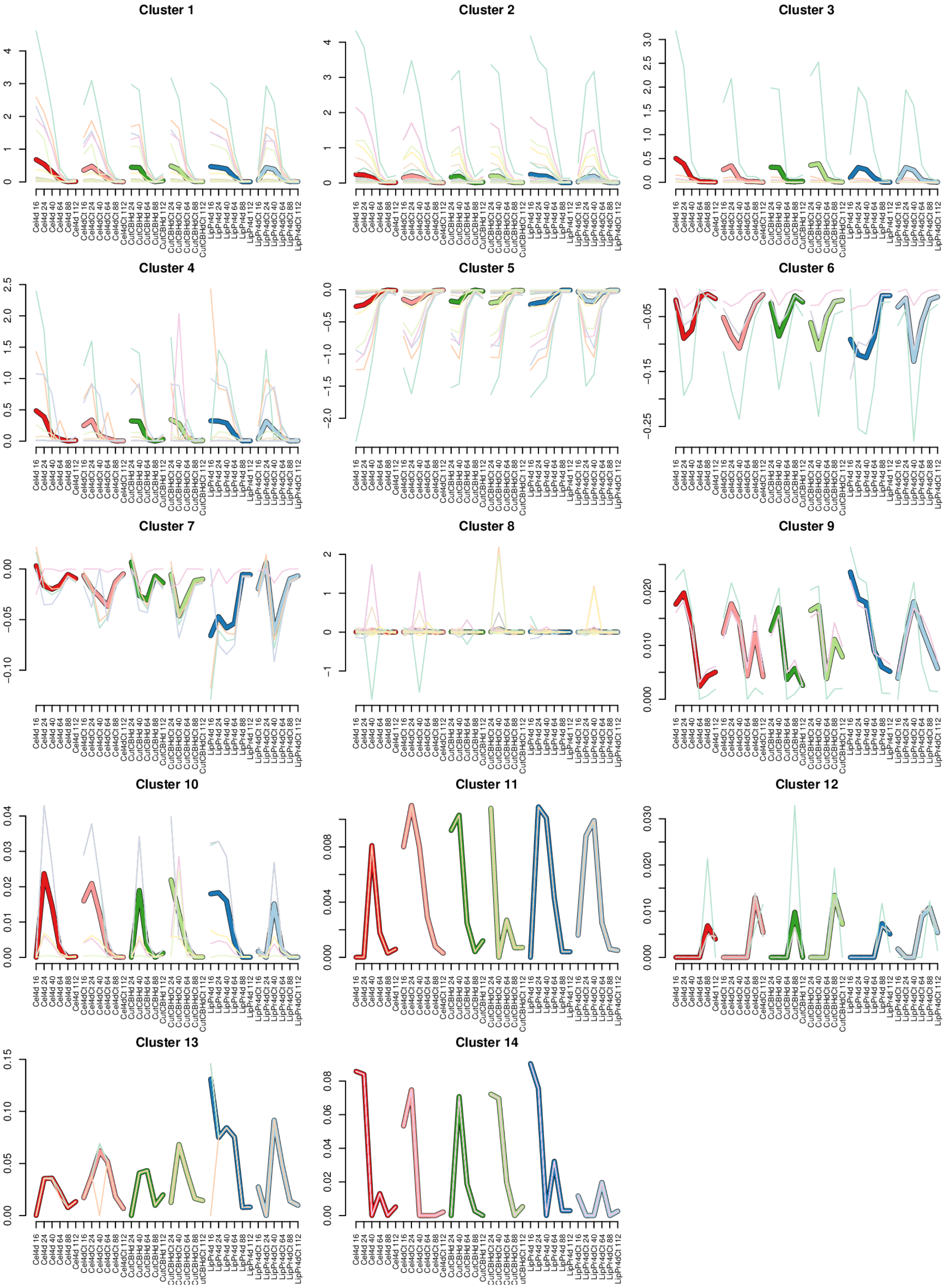

11. Glycerol identification

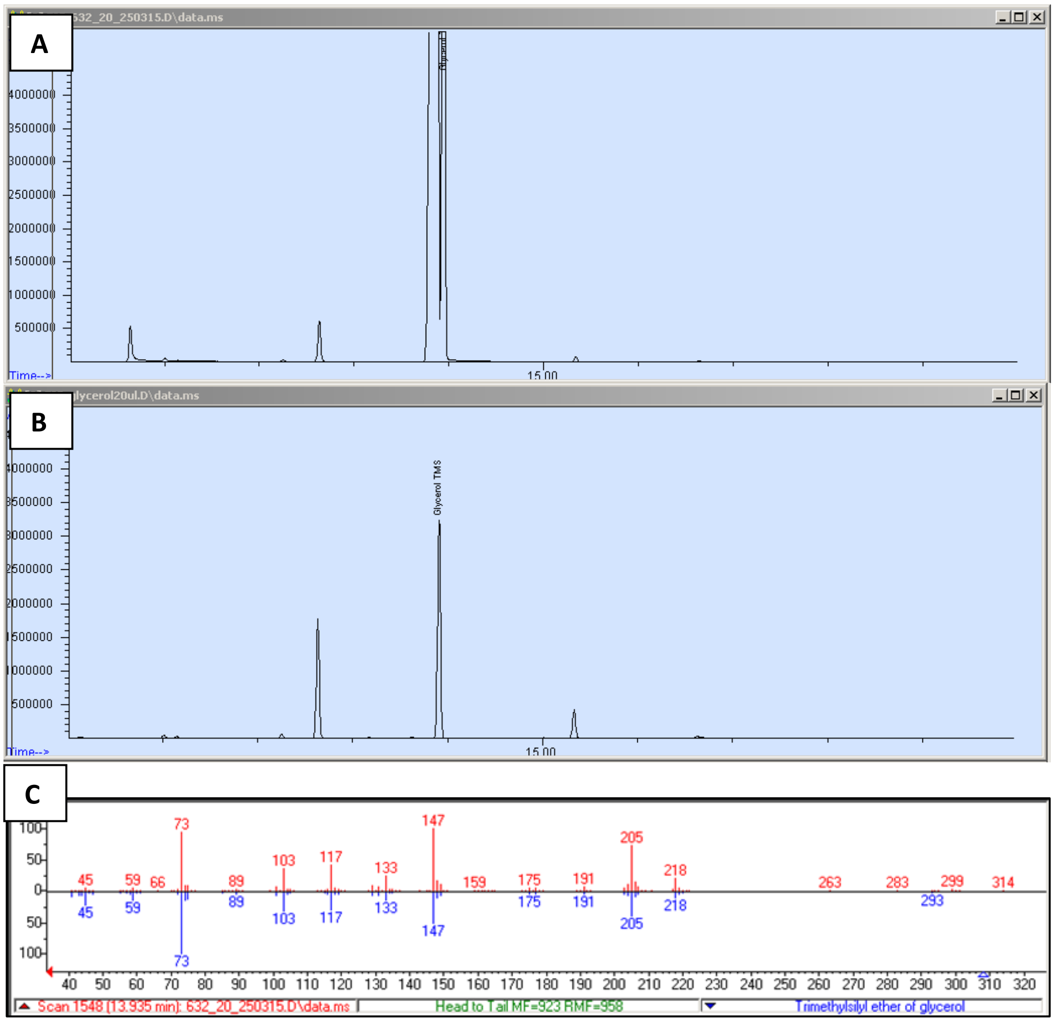

The glycerol determination was confirmed by gas Chromatography–mass spectrometry (GC/MS) The dried growth media samples (A) and glycerol reference compound (B) were derivatized with MSTFA containing 1% of TMCS. Retention time of the peak in the sample had matching retention time and mass spectra with glycerol reference compound NIST MS library search produced a good match (923) with glycerol reference spectra (C).

12. Cellotriose identification

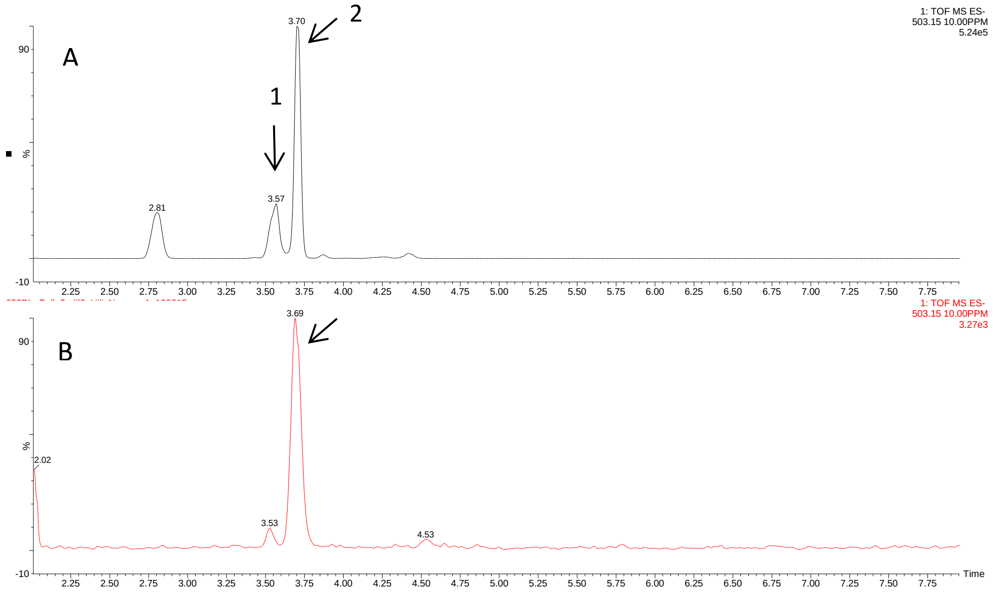

Extracted ion chromatograms, m/z 503.15 showing a growth media sample (A) and cellotriose reference compound (B). Mass spectra of the peaks 1 and 2 at retention times 3.57 and 3.70 min respectively, in the sample chromatogram were similar as the spectra of cellotriose reference compound. Based on accurate mass of $[M\text{-}H]^-$ and $[M+Cl]^-$ the compounds 1 and 2 were similar trisaccharides but the precise isomer cannot be assign based on mass spectra. The samples were analysed using ultra high performance-hydrophilic interaction liquid chromatography-mass spectrometry (UHP-HILIC-MS). The instrument was Waters Acquity Ultra Performance LC™ (UPLC) combined with Synapt G2-S mass spectrometer and Acquity UPLC™ BEH Amide (2.1 × 100 mm with 1.7 µm particles) column was used.

13. Fits of heteroscedastic Gaussian processes

In the figures the dots present actual measurement data, the solid red line is the distribution mean, coloured region indicates the 95% interval, while the dashed line includes also the observational noise model.

A. Biomass i.e. CDW (g/l)

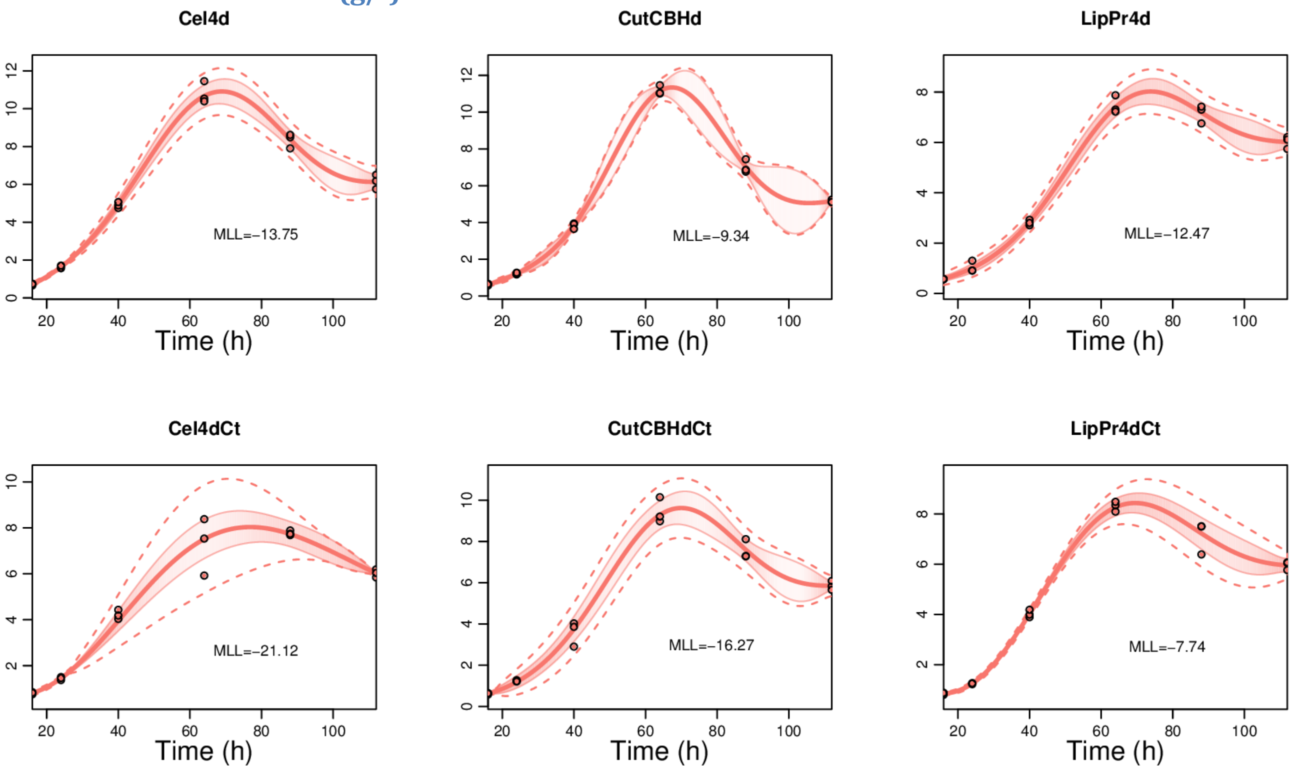

B. Extracellular protein (g/l)

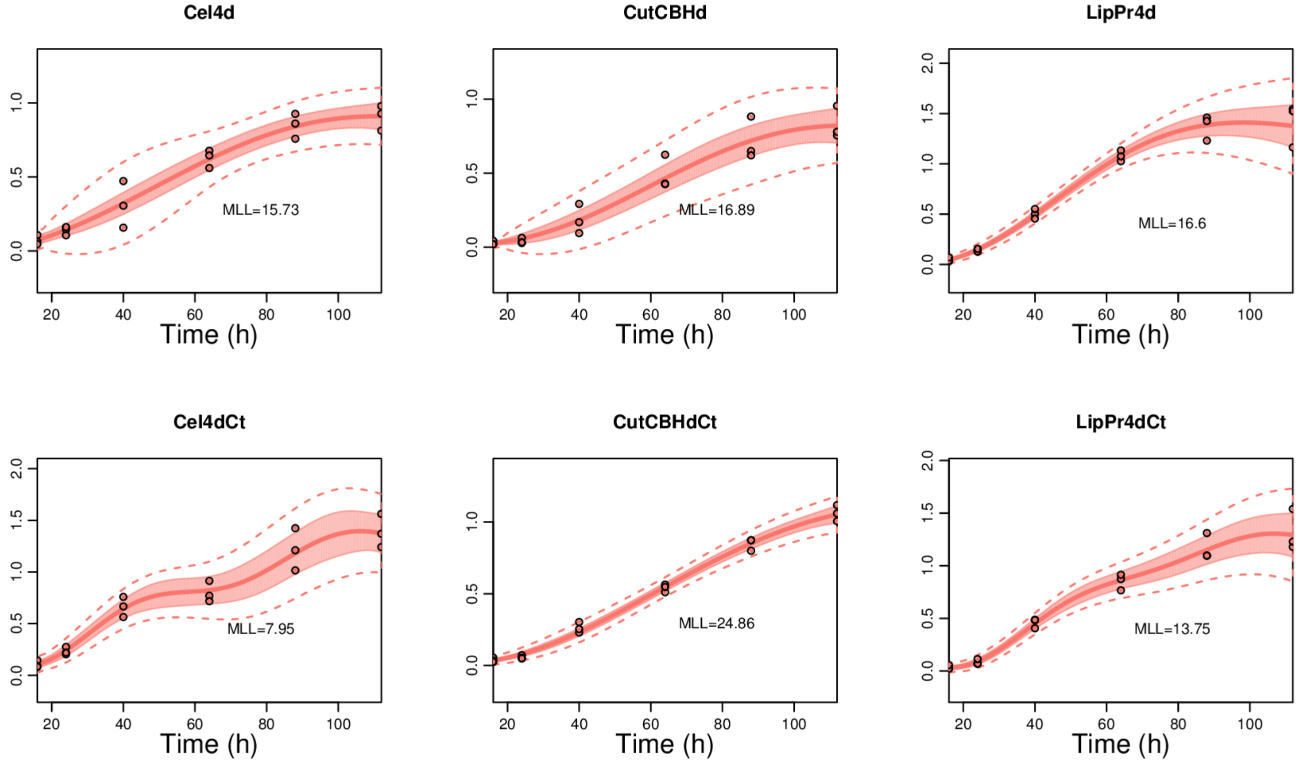

C. Cellobiose (g/l)

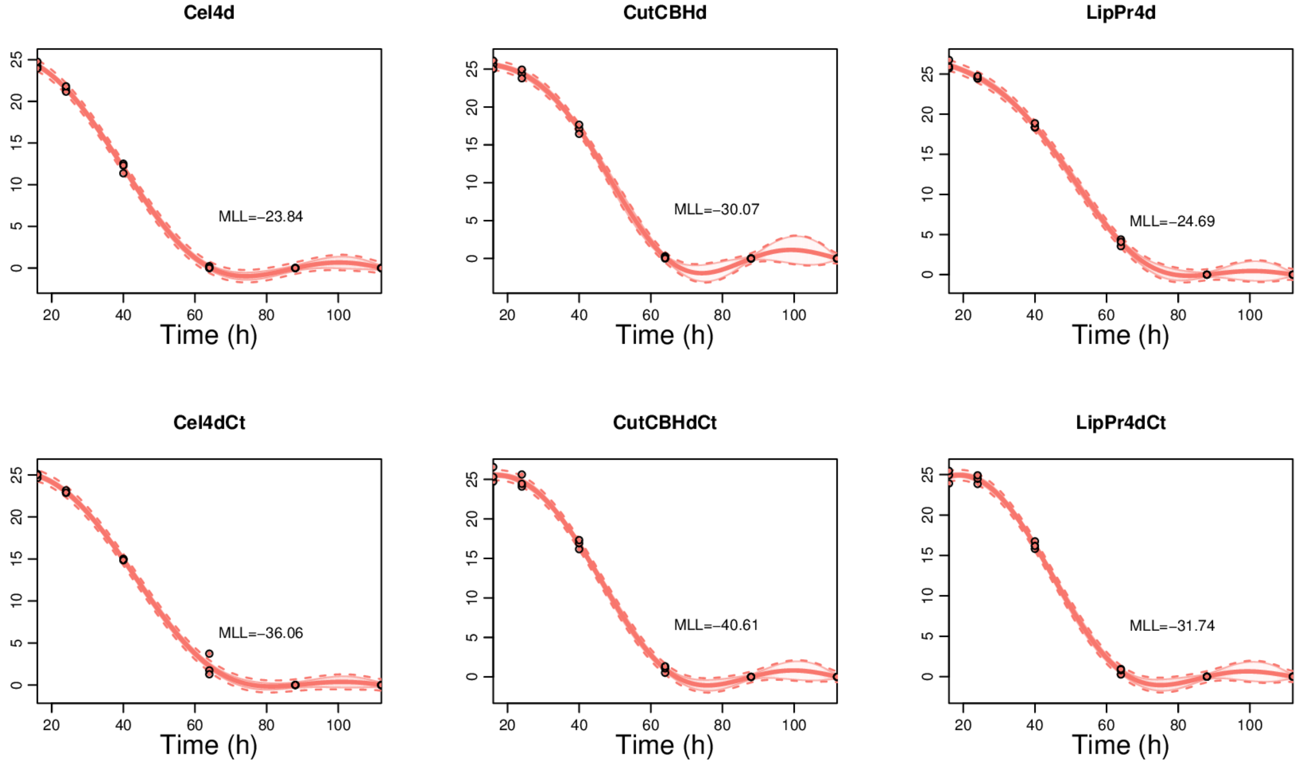

D. MUL (nkat/l)

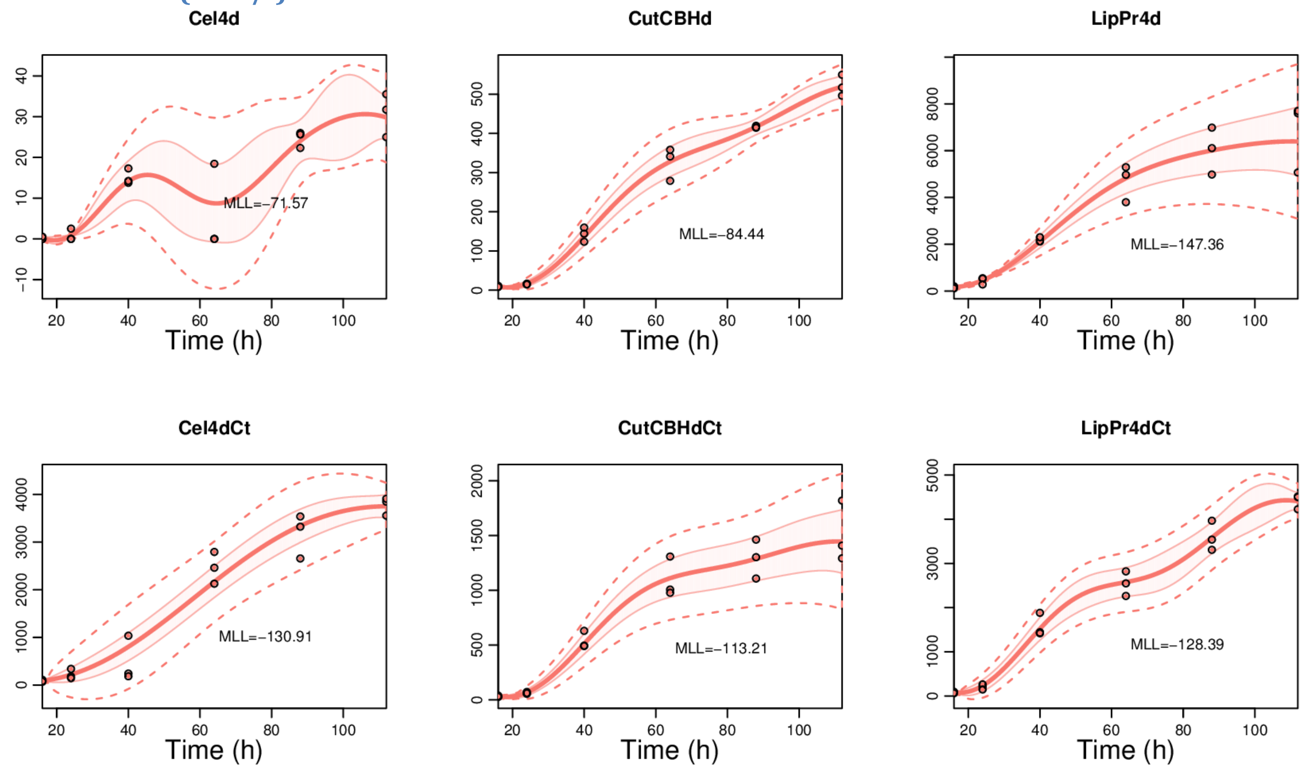

E. Cellotriose (g/l)

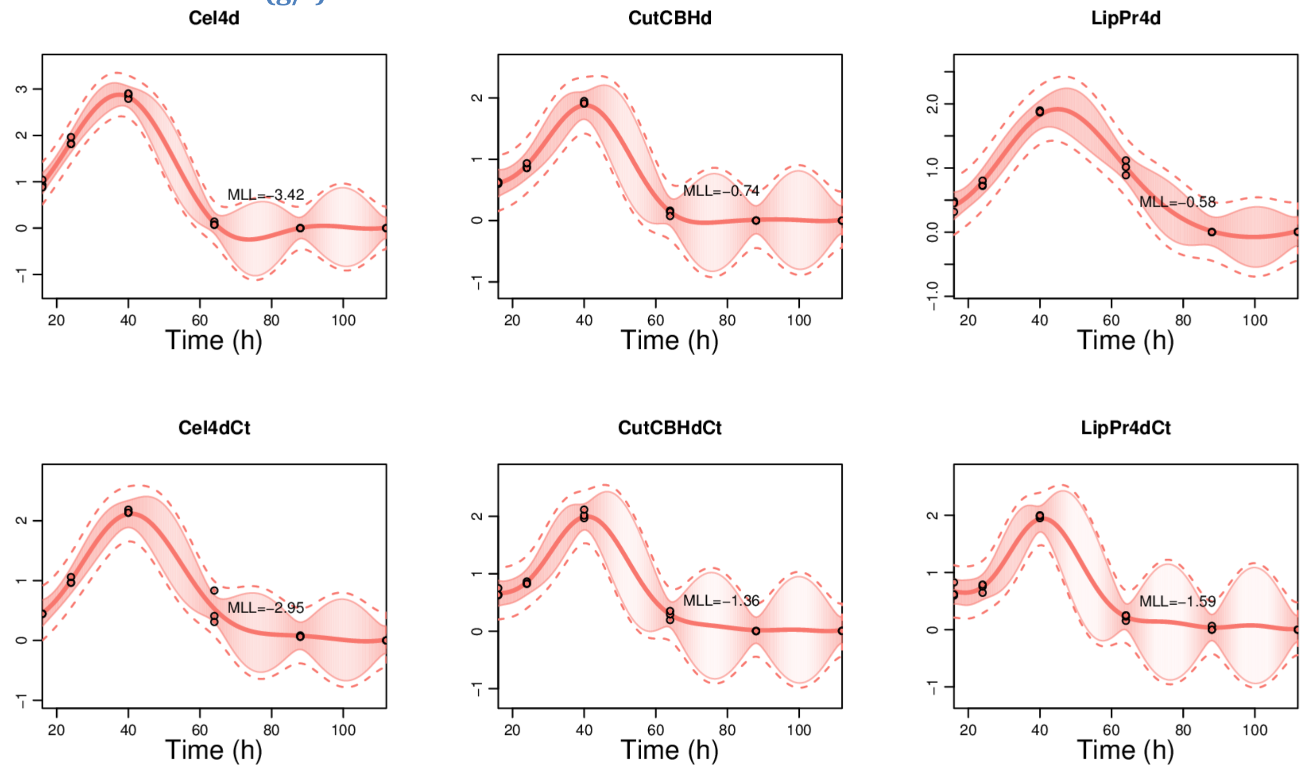

F. Glucose (g/l)

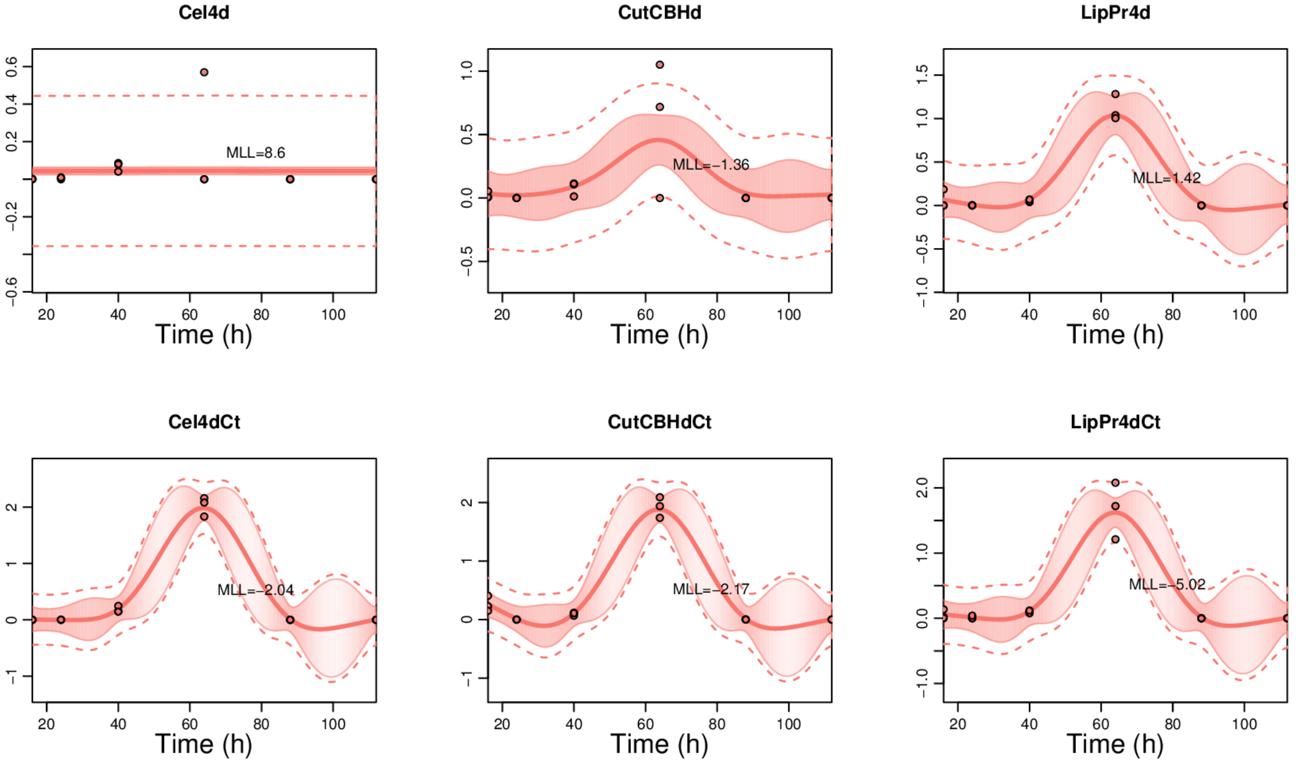

G. Glycerol (g/l)

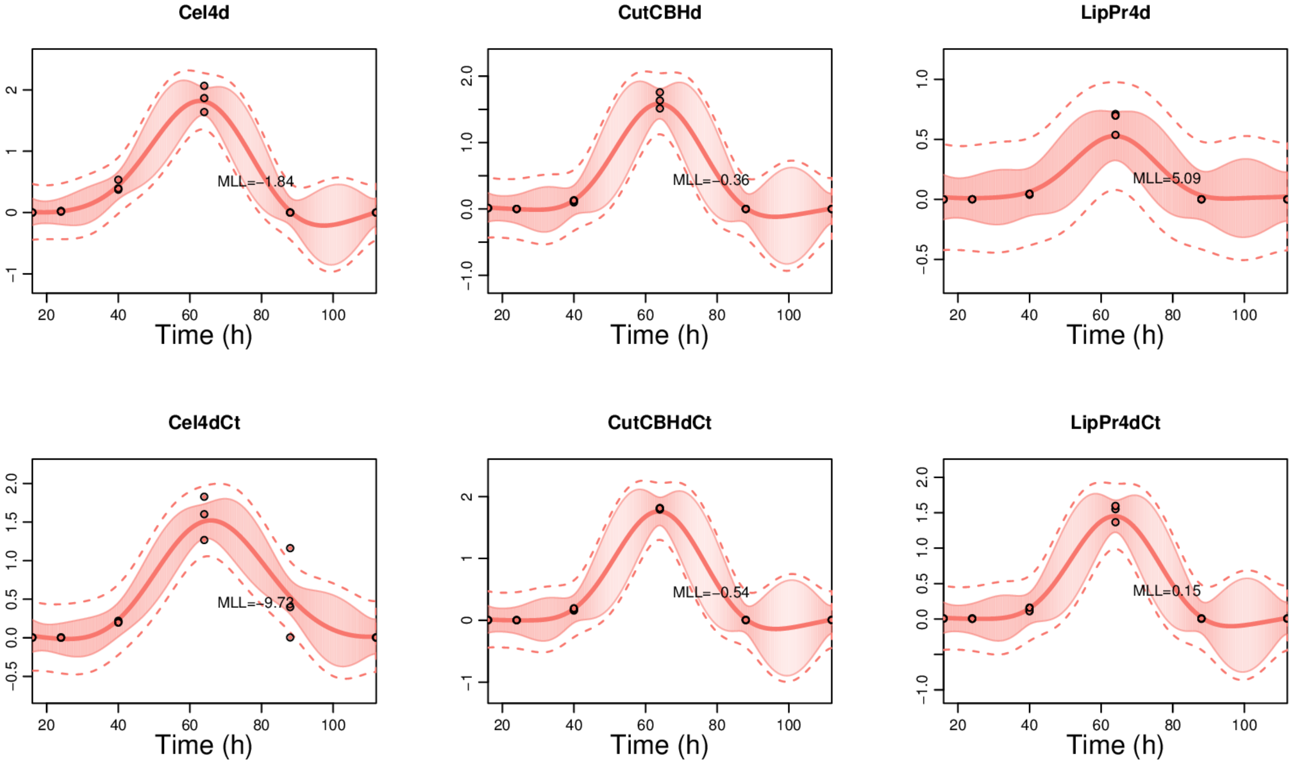

Supplement: Supplementary file 1 — 10.1186/s13068-016-0547-5 Supplementary Figures. A PDF file containing all the supplementary figures referred to in the text. [file 13068_2016_547_MOESM1_ESM.pdf]
